# Fruit bats in flight: a look into the movements of the ecologically important *Eidolon helvum* in Tanzania

**DOI:** 10.1186/s42522-020-00020-9

**Published:** 2020-08-05

**Authors:** Nistara Randhawa, Brian H. Bird, Elizabeth VanWormer, Zikankuba Sijali, Christopher Kilonzo, Alphonce Msigwa, Abel B. Ekiri, Aziza Samson, Jonathan H. Epstein, David J. Wolking, Woutrina A. Smith, Beatriz Martínez-López, Rudovick Kazwala, Jonna A. K. Mazet

**Affiliations:** 1 Karen C. Drayer Wildlife Health Center, One Health Institute, School of Veterinary Medicine, University of California, 1089 Veterinary Drive, Davis, CA USA; 2 School of Veterinary Medicine and Biomedical Sciences, School of Natural Resources, University of Nebraska, Lincoln, NE USA; 3 Department of Veterinary Medicine and Public Health, College of Veterinary Medicine and Biomedical Sciences, Sokoine University of Agriculture, Morogoro, Tanzania; 4 Tanzania National Park, Burigi-Chato National Park, Karagwe-Kagera, Tanzania; 5 School of Veterinary Medicine, University of Surrey, Guildford, UK; 6 EcoHealth Alliance, New York, NY USA; 7 Center for Animal Disease Modeling and Surveillance, School of Veterinary Medicine, University of California, Davis, CA USA

**Keywords:** Bats, *Eidolon helvum*, Tracking, Movements, Urban areas, Protected areas

## Abstract

**Background:**

Many ecologically important plants are pollinated or have their seeds dispersed by fruit bats, including the widely distributed African straw-colored fruit bats (*Eidolon helvum*). Their ability to fly long distances makes them essential for connecting plant populations across fragmented landscapes. While bats have been implicated as a reservoir of infectious diseases, their role in disease transmission to humans is not well understood. In this pilot study, we tracked *E. helvum* to shed light on their movement patterns in Tanzania and possible contact with other species.

**Methods:**

Tracking devices were deployed on 25 bats captured in the Morogoro Municipal and Kilombero District area near the Udzungwa Mountains of Tanzania. Nightly flight patterns, areas corresponding to foraging bouts and feeding roosts, and new day roosts were determined from bat movement data and characterized according to their proximity to urban built-up and protected areas. Sites for additional environmental surveillance using camera traps were identified via tracking data to determine species coming in contact with fruits discarded by bats.

**Results:**

Tracking data revealed variability between individual bat movements and a fidelity to foraging areas. Bats were tracked from one to six nights, with a mean cumulative nightly flight distance of 26.14 km (min: 0.33, max: 97.57) based on data from high-resolution GPS tags. While the majority of their foraging locations were in or near urban areas, bats also foraged in protected areas, of which the Udzungwa Mountains National Park was the most frequented. Camera traps in fruit orchards frequented by tracked bats showed the presence of multiple species of wildlife, with vervet monkeys (*Chlorocebus pygerythrus*) observed as directly handling and eating fruit discarded by bats.

**Conclusions:**

Because we observed multiple interactions of animals with fruits discarded by bats, specifically with vervet monkeys, the possibility of disease spillover risk exists via this indirect pathway. With flight distances of up to 97 km, however, the role of *E. helvum* in the seed dispersal of plants across both protected and urban built-up areas in Tanzania may be even more important, especially by helping connect increasingly fragmented landscapes during this Anthropocene epoch.

## Background

The chiropteran order is diverse with over a thousand species of bats occupying different ecological niches and providing multiple ecosystem services. Fruit bats in particular are important pollinators and seed dispersers for many plants of ecological and economic importance [1–5]. The ability of fruit bats to traverse great distances for foraging or during migration helps connect plant populations across fragmented landscapes to maintain gene flow [6–10]. Maintaining this connectivity is crucial due to threats of on-going fragmentation and degradation of forest ecosystems, leading to impeded movement of plant materials and animals among habitat patches [11–13]. In addition, hunting and habitat loss are leading to the extinction of many seed dispersers throughout the tropics, including birds, mammals, and reptiles, affecting not only their numbers, but also the plants they disperse [14, 15]. The importance of fruit bats and the ecosystem services they provide is therefore magnified when landscape fragmentation and declines in bird and mammal species facilitating seed dispersal are taken into consideration [15].

*Eidolon helvum*, the straw-colored fruit bat, is an important long distance seed disperser in tropical Africa by virtue of the diversity of plants upon which it forages and pollinates and its ability to retain seeds in its gastrointestinal tract for long periods of time and disperse them over distances greater than 70 km [16–18]. Straw-colored fruit bats are gregarious, spending daytime rest periods in large social groups often exceeding 1000 to 5000 animals at centralized roosting locations. The strong flight capabilities of *E. helvum* allow the species to take advantage of distant resources during nighttime feeding forays and return to these day roosts daily over extended periods of time, rather than frequently moving colony roost locations nomadically in search of nutritional resources [17]. With colony numbers that can reach a few thousand to several million bats [17, 19–22], their sheer numbers also enhance the extent of the ecosystem services they provide. Numbers of *E. helvum* bats in colonies vary across the year, likely in response to food availability [9, 21]. Movement patterns have also been found to change seasonally [17]. *Eidolon helvum* bats have been observed to migrate over 2000 km across many different habitat types including Zambezian woodland, forest-savanna mosaic, and lowland and riverine forests [23].

Bats are also transmission hosts of zoonotic viruses that can cause severe diseases in humans, such as Marburg virus, ebolaviruses, Nipah virus, Hendra virus, other paramyxoviruses, and coronaviruses, including Severe acute respiratory syndrome-like coronaviruses (SARS-like CoVs) and Middle East respiratory syndrome coronavirus (MERS-CoV) [24–36]. The suitability of bats as hosts for diverse viruses is likely associated with their evolutionary age, genetic diversity, broad geographical distribution, and social, biological, and immunological features [37–39]. With over 1000 recognized species, bats are the second largest order of mammals, after rodents, whose origins can be traced back to 50 million years ago [40, 41]. As social animals often living together in large numbers in close physical proximity, their colonies can facilitate the circulation and transmission of viruses and even promote virus amplification during bats’ breeding seasons [42]. Bats are unique in that they are the only mammals capable of powered flight, and it is hypothesized that the immune and metabolic changes associated with flight facilitate the large diversity of zoonotic viruses in bats as a result of altered host-virus interactions [43, 44]. Despite the implication of bats as a source of disease outbreaks in humans, the role of bats in disease emergence is not well understood; with not enough known about bat biology and insufficient measures having being put in place with respect to their conservation [45].

Previous studies have looked at genetic and epidemiological connectivities among *E. helvum* populations in Africa, as well as their role as seed dispersers in Tanzania [4, 46, 47]. While Tanzania has at least two documented *E. helvum* colonies in Dar es Salaam and Morogoro [22, 48], little is known about the movements of bats in these colonies. We aimed to understand the movement and foraging behavior of *E. helvum* in Tanzania, using Global Positioning System (GPS) loggers, and to investigate how this behavior relates to the connectivity of landscapes and the bats’ interactions with other species, including humans. Our objectives were to explore the fine-scale movement ecology of *E. helvum* in Tanzania and to also use this information to inform environmental surveillance of indirect contact of bats with other species. We hope that the insights gained from this work will contribute to the existing body of knowledge on *E. helvum* and be helpful for both the conservation of fruit bats and for informing on the reduction of bat-human interaction and pathogen transmission risk.

## Methods

### Study sites and animals

We tracked the movements of straw-colored fruit bats (*E. helvum*) living in two colonies in Morogoro Region, Tanzania in November 2016. The first site (−6.8233, 37.6662) comprised a bat colony in the Morogoro Municipal area, with a population of 315,866 people [49], while the second site (−7.6621, 36.9873) was in Kilombero District, by the Illovo Sugar Company near the Udzungwa Mountains National Park. The Kilombero site was close to sugarcane plantations headquartered in nearby Kidatu, which has a population of 32,589 people [49]. Both colony locations were amid human dwellings and a school. While a formal estimate of the population of bats at these colony sites was not carried out, they were roughly estimated to be at least several thousand in number, and the Morogoro colony’s roost size was previously estimated at 10,000 [22]. The bats were captured with synthetic mist nets suspended between two metal or wooden poles placed near tree roosts, carefully removed from the nets, and temporarily restrained in a closed cotton bag. Handling, sedation and reversal (dexmedetomidine/atipamezole), and release were performed in accordance with the University of California, Davis, Institutional Animal Care and Use Committee (IACUC) (protocol number: 19300). Only adult male and female bats not observed to be pregnant were considered for GPS-logger attachment, in order to ensure that the weight of the GPS-loggers did not exceed 5–10% of bat body mass [50, 51]. All work in this study was conducted with approvals and permissions from the Tanzania Wildlife Research Institute (2016–290-NA-2011-29).

#### GPS logger attachment

We used two types of GPS loggers: 15 g solar (e-obs, Munich, Germany) and 3.5 g PinPoint Argos satellite tags (Lotek Wireless Inc.), and aimed to attach GPS loggers to at least eight bats at each study site to collect sufficient movement data for analyses. This minimum number was determined based on the percentages of successfully tracked *E. helvum* bats in previous studies (for tags attached to bats via adhesives) [16, 17, 23]. Tags were attached to bats by clipping the hair between their shoulder blades, applying Skin-Tac-H Adhesive wipe (Torbot Group Inc.) on the clipped area, and then affixing tags with cyanoacrylate glue [51]. To compare longevity of tag application, we attached one Argos satellite tag using a collar tied with a degradable suture, Vicryl® (polyglactin 910), so that while the collar might stay on longer than loggers attached with adhesive glue, it would eventually drop-off with no long-term adverse effects on bat health [16].

#### GPS logger setup

The solar e-obs tags collected acceleration-informed GPS fixes between 17:00–07:00, with acceleration data recorded upon 3-axes: X = left-right, Y = backward-forward, and Z = up-down. We programmed tags to collect acceleration data all day at intervals of 60 s at a byte count of 1135 (16.67 Hz). GPS fixes were collected at intervals of 45 min during GPS on-times, unless the bat was moving at a speed of 50 cm/s or more, in which case collection intervals changed to every 30 s. Loggers started collecting data immediately after bats were released with affixed loggers. To download the GPS and associated acceleration data, we walked at least once each day through the bat colonies with the e-obs base station connected to a directional high-gain antenna. The base station automatically downloaded the data when it connected with a logger. We programmed the Argos satellite tags at different intervals, starting with every 1.5 h in the beginning of the study, to every 30 min, as our study progressed, in order to balance tag battery life with the number of discrete GPS fixes recorded and with the days the tag remained attached to the bat. Data from the Argos satellite tags were downloaded remotely from the Argos website (http://www.argos-system.org/).

### Analyses of movement data

#### Estimation of foraging areas/feeding roost locations and new day roosts

**Fig. 1:**
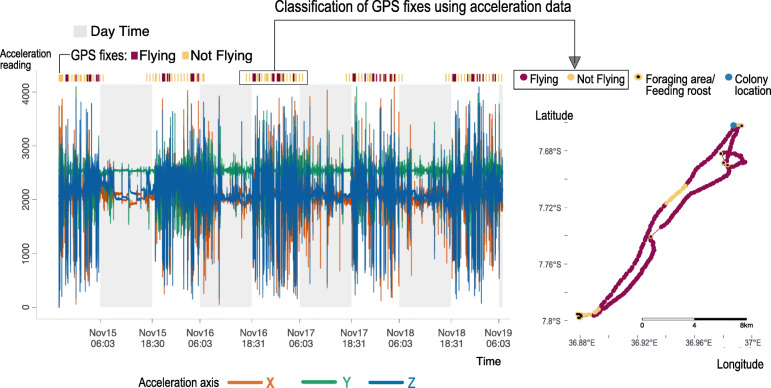
Left: Classification of GPS fixes based on temporally associated acceleration bursts along Y- (backward-forward) and Z- (up-down) axes. Right: The GPS fixes corresponding to a bat’s third foraging night, classified as ‘Flying’ and ‘Not Flying’, and further subsetted into foraging areas/feeding roost locations

The GPS points collected from e-obs tags were categorized as ‘flying’ or ‘not-flying’ based on the variances of their temporally corresponding acceleration bursts [16]. Previous studies have differentiated acceleration bursts with higher variances in their Y- (backward-forward) and Z- (up-down) axes, corresponding to ‘flying’ behavior, from those with lower variances (‘not-flying’ behavior) by k-means clustering [16]. We compared the classification of GPS points into ‘flying’ and ‘not-flying’ behavior using XZ, YZ, and XYZ axes, and observed no significant difference between them (Additional file 1: Figure S1 and Table S1). Using the GPS classification based on the YZ axes (Additional file 2: Figure S2), we further subsetted sequential ‘not-flying’ GPS fixes within a radius corresponding to the distance a bat could travel at its average speed (determined by averaging the ground speeds recorded for each GPS fix) and with a time interval greater than or equal to 30 s. These subsetted GPS fixes were considered to comprise both foraging bouts (i.e. moving within a food tree) as well as night feeding roosts (Fig. 1). Points falling in the vicinity of the original colony where bats were tagged were excluded from classification as foraging areas/feeding roosts or new day roosts. A day roost location was one where a bat was recorded as present between sunrise and sunset.

The mean and range of cumulative distance flown each night as well as each hour was determined. In addition, the maximum straight-line distance between day roosts and the most distant foraging sites from the colony location was determined. The utilization distribution of each bat was calculated and mapped using classic kernel density estimation (KDE) with vertices set to 50 and 95% location probabilities respectively, using the adehabitatHR R package [52].

#### Distance of foraging areas/feeding roost locations from urban built-up and protected areas

We measured the distance of each foraging area/feeding roost location to its nearest urban built-up and protected area (national park or reserve) using GDAL v2.4.1 (https://gdal.org) and GRASS GIS v7.0.5 (https://grass.osgeo.org) [53]. For determining urban built-up areas, we used the Global Urban Footprint (GUF), which is high resolution human settlement/urban footprint data derived from TerraSar-X satellite imagery [54, 55]. Originally at a resolution of 12 X 12 m, we aggregated urban built-up areas into grids of 100 X 100 m such that any larger grid containing at least one smaller grid classified as built-up was assigned as urban [17]. In order to determine the distances of foraging locations from protected areas in Tanzania, we used data on game reserves, nature reserves, forest reserves, and national parks from the World Database of Protected Areas (WDPA) [56]. The GUF and WDPA data were accessed in February 2016 and 2019, respectively.

Unless otherwise noted, all analyses were performed with R v3.6.0 [52, 57–73]. The code used in the analyses is available at https://nistara.github.io/Tanzania-fruit-bat-study/.

### Site characterization of foraging areas/feeding roost locations and environmental surveillance

A convenience sample of locations identified as foraging areas/feeding roosts (or in proximity with them) or new day roosts by the tracking data were visited to determine types of trees at the site, presence of human dwellings, and to confirm whether there were any wild or domestic animals or signs of them at the sites. Where possible, we talked with the local community members about their observations on bat activity at these sites. Two remote-triggered field camera traps (Reconyx™ Hyperfire HC600) were set up at the horticultural garden at the Sokoine University of Agriculture (SUA), Morogoro, Tanzania (Fig. 6), in order to understand the potential for other species to contact fruits discarded by bats at this foraging site. The SUA horticultural gardens were chosen because they were identified as being visited by the tracked bats, who fed upon mangoes at this site. Fruit bat activity at this site was confirmed by the presence of mangoes eaten and subsequently discarded by the bats under the mango trees. The camera traps were fixed under mango trees 20–30 cm from the ground to capture images of both domestic and wild animals. They were active 24 h a day from 18th December 2017 to 2nd January 2018. Because the bats were originally tracked in November 2016 during the mango harvesting season, we waited for the next mango season (December 2017) to set up the camera traps under circumstances similar to the tracking period.

## Results

### GPS logger data, foraging areas/feeding roost locations, and new day roosts

**Fig. 2:**
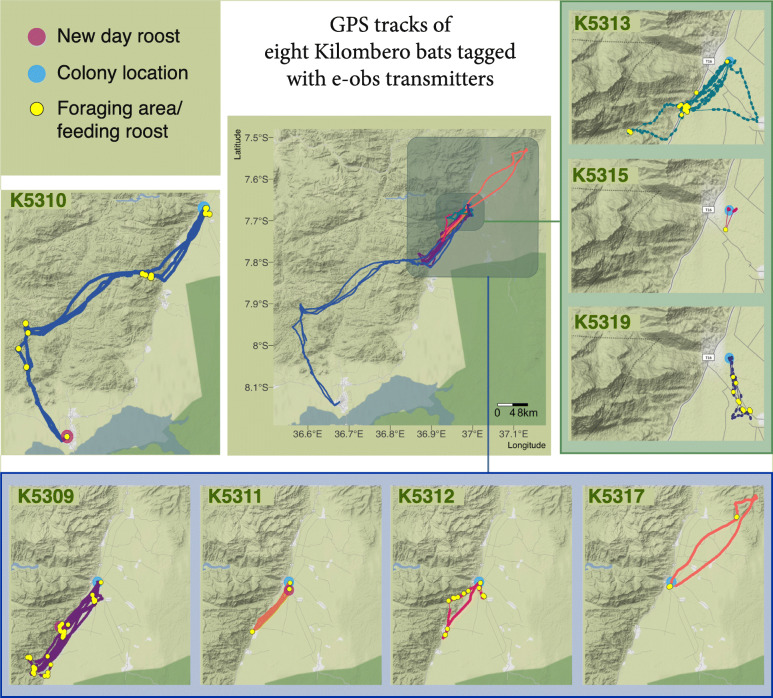
Map of the tracking data obtained from eight out of ten e-obs tags attached on *Eidolon helvum* bats in the Kilombero District area

**Fig. 3:**
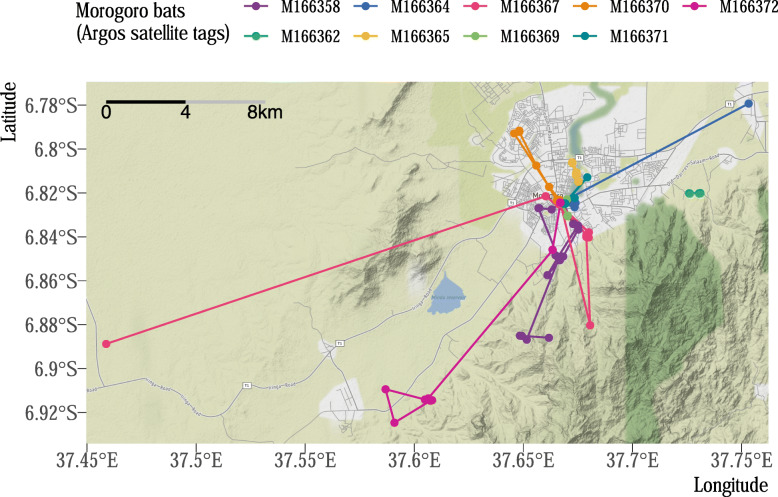
Map of the tracking data obtained from nine of the ten bats tagged with Argos satellite tags in Morogoro

Twenty five *E. helvum* bats were tagged across the two study sites: 15 bats in Kilombero (10 e-obs tags and five Argos satellite tags), and 10 bats in Morogoro (all Argos satellite tags). Data was retrieved from 21 of these bats (Kilombero: 8/10 e-obs tags and 4/5 Argos tags, Morogoro: 9/10 Argos tags) (Table 1). The mean logger mass of the tracked bats did not exceed 5.54% (mean ± SD: 2.92 ± 1.97, min: 1.13, max: 5.54) of bat body weight (271 ± 30 g). The bats were predominantly males (84%) because their larger body mass kept the percent tag weight low and facilitated their inclusion in the study (males: 277 ± 25.8 g, females: 240 ± 34.8 g). The tag affixed with a collar stayed on the bat for the longest time period (14 days compared to a maximum of six days for tags attached by adhesives). We collected data for 1–6 foraging nights per bat; even though the collared bat (M166367) was tracked for 14 days, its GPS fixes corresponded to 6 foraging nights. The number of GPS fixes from e-obs tags (n: 31662) vastly outnumbered those by the Argos satellite tags (n: 156). We identified 505 foraging areas/feeding roost locations and three new day roost locations from the high-resolution e-obs data (Table 2 and Fig. 2). In contrast to the e-obs tag data, we were unable to classify Argos satellite tag fixes as foraging or not because of the inability to assess time spent at each location and whether the bat was in flight or not. Nonetheless, they still provided information on areas frequented by bats (Fig. 3 and Additional file 3: Figure S3).

**Table 1:** Deployment summary, nights tracked, cumulative flight distances, and kernel density estimates of habitat utilization of tracked bats

Bat	Location	Tag	Sex	Body weight (g)	Date deployed (in 2016)	Nights with fixes	No. of GPS fixes	Mean (min, max) nightly cumulative distance (km)	KDE 50% (ha)	KDE 95% (ha)
K5309	Kilombero	e-obs	M	284	14-Nov	5	9212	44.04 (37.65, 50.48)	3326.90	11,743.12
K5310	Kilombero	e-obs	M	310	14-Nov	5	10,806	55.83 (2.07, 97.57)	33,513.36	124,413.78
K5311	Kilombero	e-obs	M	272	14-Nov	2	1670	22.68 (22.31, 23.06)	1514.94	4861.94
K5312	Kilombero	e-obs	M	293	15-Nov	3	1957	12.48 (0.73, 28.82)	1527.30	6497.41
K5313	Kilombero	e-obs	M	271	14-Nov	6	3804	12.24 (7.93, 22.91)	142.25	1437.45
K5314	Kilombero	e-obs	M	276	15-Nov	–	–	–	–	–
K5315	Kilombero	e-obs	M	293	15-Nov	1	340	2.92 (2.92, 2.92)	23.62	96.88
K5316	Kilombero	e-obs	M	281	15-Nov	–	–	–	–	–
K5317	Kilombero	e-obs	F	271	15-Nov	4	2846	14.3 (0.33, 52.14)	4193.79	18,985.86
K5319	Kilombero	e-obs	M	300	15-Nov	2	1027	8.09 (5.58, 10.60)	118.16	469.22
K166357	Kilombero	Argos	M	310	16-Nov	–	–	–		–
K166359	Kilombero	Argos	M	276	16-Nov	1	11	5.13 (5.13, 5.13)	195.64	1193.77
K166361	Kilombero	Argos	F	261	16-Nov	2	8	0.1 (0, 0.20)	196.62	1257.64
K166363	Kilombero	Argos	M	273	16-Nov	1	3	0.06 (0.06, 0.06)	–	–
K166366	Kilombero	Argos	M	263	16-Nov	1	12	0.77 (0.77, 0.77)	8.79	51.49
M166364	Morogoro	Argos	M	288	03-Nov	3	6	0.35 (0, 1.06)	2832.96	16,753.90
M166367	Morogoro	Argos	M	276	03-Nov	6	10	1.12 (0, 4.45)	7909.28	51,330.65
M166358	Morogoro	Argos	M	294	18-Nov	2	30	9.99 (9.84, 10.15)	868.67	4277.60
M166360	Morogoro	Argos	M	280	18-Nov	–	–	–	–	–
M166362	Morogoro	Argos	M	200	18-Nov	1	4	0.66 (0.66, 0.66)	–	–
M166372	Morogoro	Argos	F	236	18-Nov	3	22	6.19 (0.01, 18.36)	2212.86	14,106.77
M166365	Morogoro	Argos	M	300	18-Nov	1	6	1.69 (1.69, 1.69)	22.34	109.96
M166369	Morogoro	Argos	F	193	18-Nov	1	18	2.62 (2.62, 2.62)	26.06	115.78
M166370	Morogoro	Argos	M	251	18-Nov	2	15	3.25 (1.99, 4.50)	565.31	2387.27
M166371	Morogoro	Argos	M	230	18-Nov	1	11	4.95 (4.95, 4.95)	36.61	242.45

**Table 2:** Number and characteristics of foraging areas/feeding roost locations and new day roosts for e-obs tagged bats

Bat	Forage/feed roost GPS fixes	Maximum straight-line distance to forage/feed roost sites (km)	New day roosts (corresponding no. of GPS fixes)	Maximum straight-line distance between day roosts (km)	Forage/feed roost fixes within 100 m of built-up areas	Forage/feed roost fixes within 100 m of protected areas	Protected areas where bat had forage/feed roost GPS fixes
K5309	150	19.83	1 (30)	10.50	26.67% (40/150)	54% (81/150)	Udzungwa Mountains National Park
K5310	84	62.63	1 (30)	62.86	11.90% (10/84)	69.05% (58/84)	Udzungwa Mountains National Park
K5311	25	11.03	1 (55)	2.50	84.00% (21/25)	0% (0/25)	–
K5312	45	11.17	0	–	77.78% (35/45)	17.78% (8/45)	Udzungwa Mountains National Park
K5313	108	6.37	0	–	91.67% (99/108)	4.63% (5/108)	Udzungwa Mountains National Park
K5315	12	0.97	0	–	0% (0/12)	0% (0/12)	–
K5317	48	16.68	0	–	0% (0/48)	25% (12/48)	Mikumi National Park
K5319	33	2.84	0	–	81.82% (27/33)	0% (0/33)	–

**Fig. 4:**
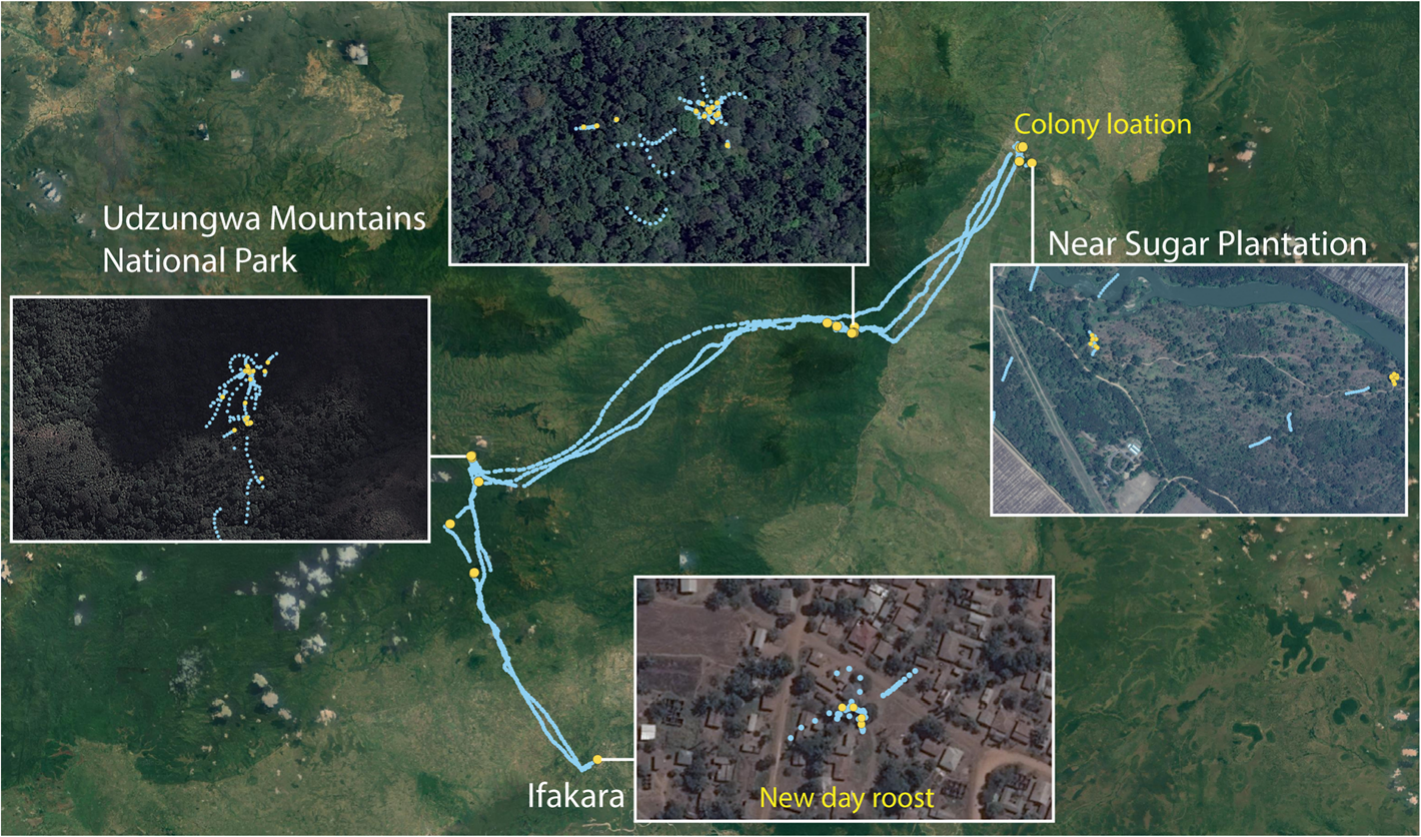
The flight path of bat K5310, who frequented both protected and urban areas, flying a cumulative distance of up to 97.57 km during a single foraging night (yellow circles depict foraging areas/feeding roost locations)

**Fig. 5:**
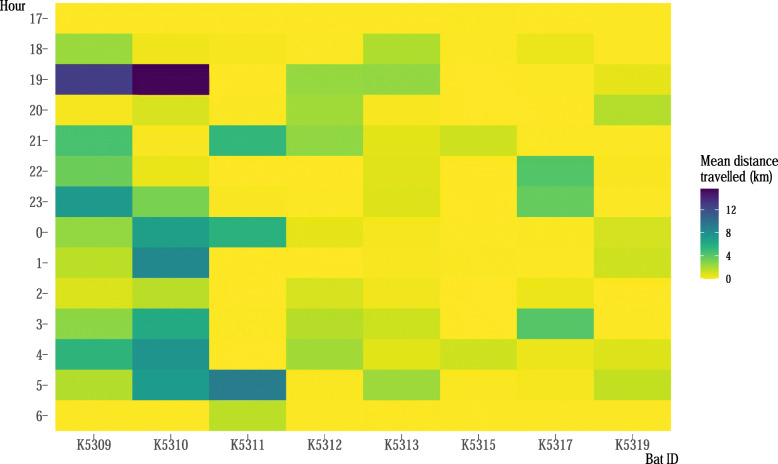
Heatmap of the mean hourly distances (km) flown by individual bats on foraging nights

The mean cumulative nightly flight distance observed for e-obs tagged bats was 26.14 km (min: 0.33, max: 97.57). One male bat, K5310, flew cumulative nightly distances of 96.46 and 97.57 km on two consecutive nights, the longest nightly distances flown by any of the tracked bats, connecting his roost location in the Kilombero site to a newly identified day roost in Ifakara, Kilombero District (Fig. 4, Additional file 4: Table S4 and Figure S4). He foraged in urban, semi-urban, and intact forest landscapes along his flight path. Bat K5310 also had the maximum hourly distance flown of any bat: 38.08 km flown between 1 and 2 am on the third tracked night (Additional file 5: Table S5.1 and Figure S5). The mean hourly distances flown by all bats is shown in Fig. 5 and presented in Additional file 5: Table S5.2. Home range sizes varied between 51.49 and 124,413.78 ha based on 95% KDE (Table 1 and Additional file 6: Figure S6). The bats tended to visit the same sites on subsequent nights (Additional file 7: Figure S7), and while all male e-obs tagged bats flew in a direction south of their roosting colony, the only female e-obs tagged bat (K5317) flew in the northern direction (Fig. 2).

### Distance of foraging areas/feeding roost locations from urban built-up and protected areas

Of the 505 identified foraging areas/feeding roost locations obtained from *E. helvum* bats tagged with e-obs loggers in Kilombero, approximately 25% (126/505) were in areas classified as built-up by the Global Urban Footprint while 46% (232/505) were within 100 m of built-up areas (Table 2) [54]. Even though we could not ascertain foraging areas from the Argos satellite tags, distances of GPS fixes from urban areas excluding the tagging location revealed that approximately 60% (84/139) of these locations were within urban areas, and 77% (107/139) were in proximity (within 100 m) to built-up landscapes.

In the case of protected areas, approximately 32% (164/505) of the Kilombero *E. helvum* foraging points were in or within 100 m of a national park or reserve, the majority (93%) of them in Udzungwa Mountains National Park, followed by Mikumi National Park (Table 2). Eleven GPS fixes from the Morogoro bats were within 100 m of protected areas, all in the Nyandira Reserve.

### Site characterization and environmental surveillance

We visited nine sites for habitat characterization and while we did not hone in directly on GPS fixes to identify the exact trees visited by bats, we observed the general diversity of trees present at these sites. Sixteen species of trees (Table 3) were identified and conversations with community members at these sites revealed that mangoes were a favorite of *E. helvum*. During mango season (November–December), the bats were reported to feed on mango fruit and in addition to the noise they made while feeding at night, they also caused destruction of the mango harvest, scattering chewed-upon mangoes on the ground. Five of the sites visited by us corresponded to newly identified day roost locations. We did not observe any new roosting colony, although community members at the Ifakara site pointed out palm trees that served as colony roost locations in the previous year (2015).

**Table 3:** Characteristics of nine sites frequented by *Eidolon helvum*

No.	Site (Lat, Long)	Human dwellings/buildings	Wild animals or signs of animals	Domestic animals or their signs	Trees observed
1	Ifakara^†^ (−8.136, 36.987)	Yes	None observed	Chickens, dogs, cats	*Syzygium guineese,*
					*Elaeis quineensis,*
					*Muntingia calabura,*
					*Mangofera indica,*
					*Musa* spp.
2	Mang’ula, Udzungwa Mountains National Park (−7.799, 36.878)	No	Non-human primates	None observed	Sorindea madagascarensis,
					*Psychotria capensis,*
					*Leptactina platyphylla*
3	Msolwa ujamaa, Udzungwa (−7.744, 36.929)	Yes	Non-human primates	Chickens, ducks, goats, dogs, cats	*Cocos nucifera*,
					*Mangofera indica,*
					*Azadirachta indica,*
					*Ficus* spp.,
					*Milicia excelsa*
4, 5	Udzungwa^†^	Yes	Non-human primates	None observed	*Syzygium guineese*,
	(−7.731, 36.929)				*Mangofera indica*,
	(−7.731, 36.922)				*Psidium guajava*,
					*Anacardium occidentale*
6	Kidatu (−7.683, 36.966)	Yes	None observed	Chickens, dogs, cats, goats, pigs	Syzygium guineese,
					*Mangofera indica*,
					*Citrus* spp. (orange),
					*Ficus* spp.,
					Mishoki^*^
7, 8	Kilombero, Illovo^†^	No	None observed	None observed	*Ficus* spp.
	(−7.673, 36.987)				
	(−7.673, 36.988)				
9	Horticulture garden, Morogoro (−6.845, 37.663)	Yes	Via camera traps: Non-human primates, mongoose, rat, cat	None observed	*Mangofera indica*

^*^Local name for tree not identified by common English names

^†^These locations were also newly identified day roost sites

**Fig. 6:**
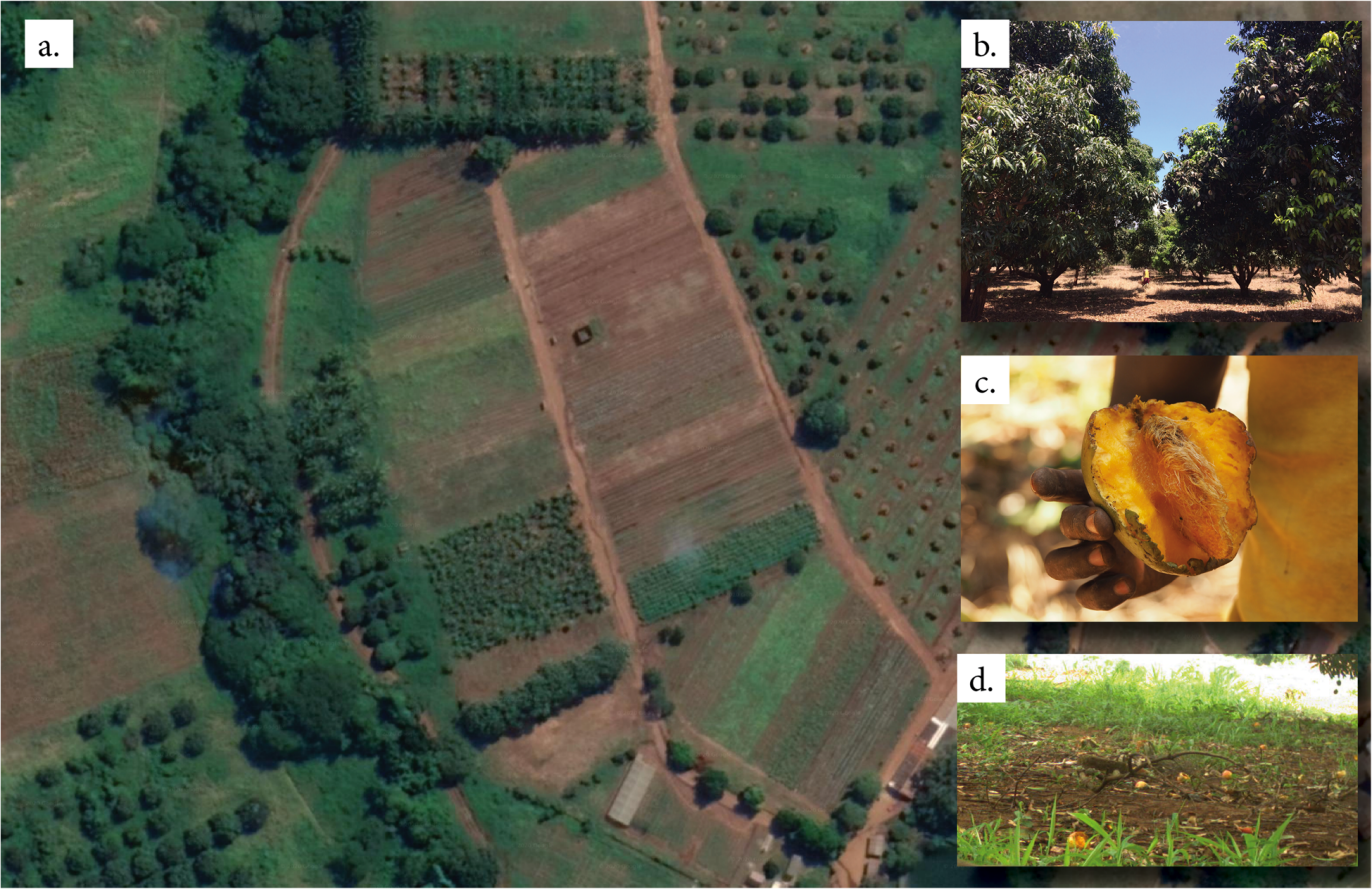
a. Site for setting up camera-traps, identified by tracking data. b. Worker harvesting mangoes at this site. c. Half-eaten mango discarded by foraging bats. d. Discarded mangoes on the ground under mango trees

**Fig. 7:**
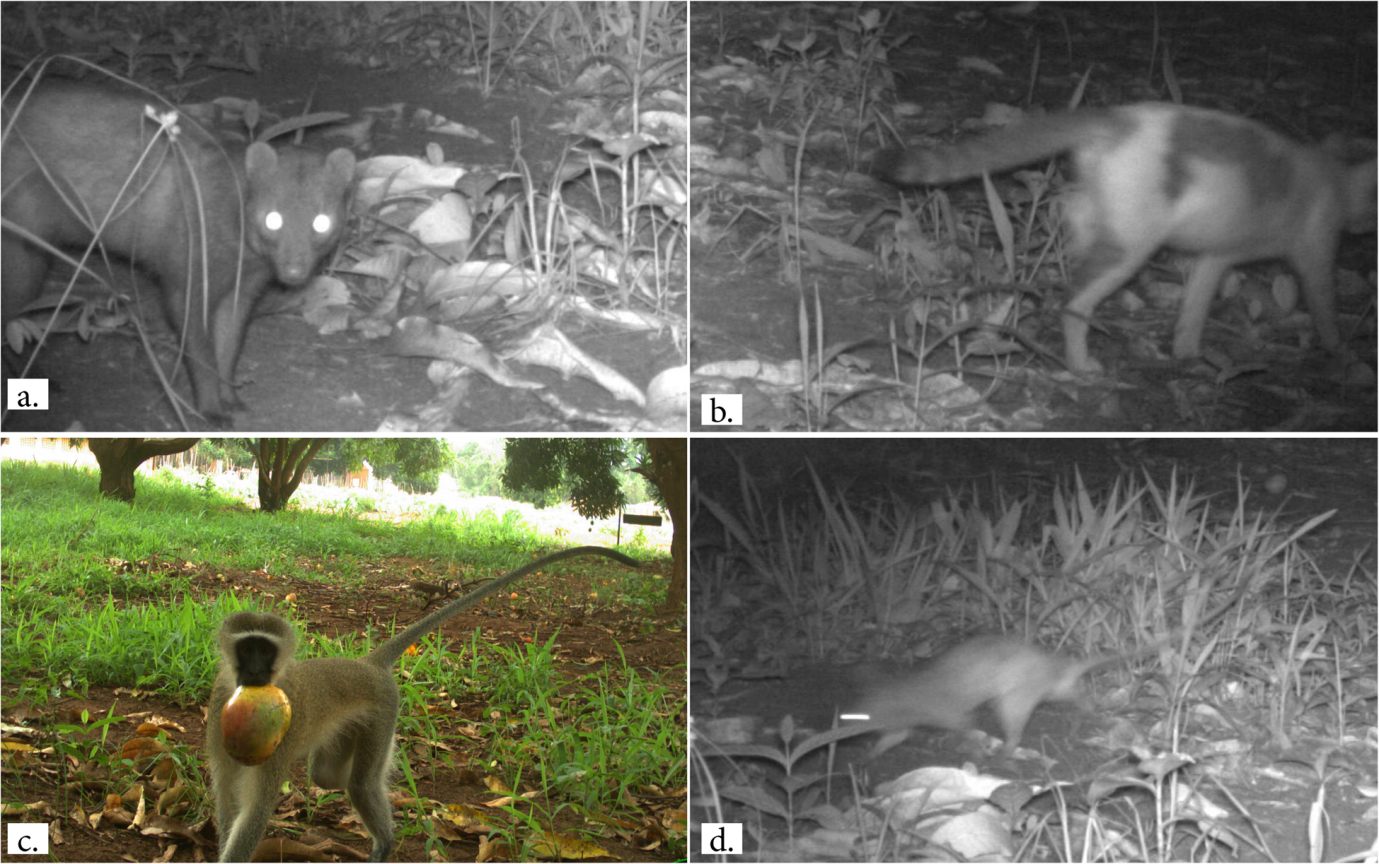
Pictures of species detected at the camera-trap site. **a** Common dwarf mongoose (*Helogale parvula*). **b** Domestic cat (*Felis catus*). **c** Vervet monkey (*Chlorocebus pygerythrus*). **d** Black rat (*Rattus rattus*)

At the horticulture garden in Sokoine University of Agriculture (SUA) in Morogoro, we directly observed discarded mangoes that had been chewed and left behind by fruit bats (Fig. 6). Camera-traps set up at this orchard showed visits by five mammals: vervet monkeys (*Chlorocebus pygerythrus*), domestic cat (*Felis catus*), common dwarf mongoose (*Helogale parvula*), *Rattus rattus*, and humans (Fig. 7). Vervet monkeys at the site were observed handling fallen or discarded mangoes and putting them in their mouths (Fig. 7 and Additional file 8). The people observed at the camera trapping site were predominantly adults, including farm workers, security guards, and visitors who were there to collect fallen mangoes.

## Discussion

Our study takes an integrative approach to better understand *E. helvum* ecology in Tanzania, looking at both their movement patterns and identification of potential pathways of pathogen transmission among bats and other species. In the first work of this kind in Tanzania, results reveal that one straw-colored fruit bat, *E. helvum*, flew up to 97 km over a single foraging night, frequenting both urban built-up and protected areas. With maximum straight-line distances of 62 km from day roosts to foraging areas/feeding roost locations, their foraging distances enable *E. helvum* to connect land fragmented by human activities and provide valuable seed dispersal and pollination services across vast distances [16–18]. Seed dispersal by *E. helvum* can help regenerate forests in degraded landscapes and can also contribute to the local economy through support for the propagation of economically important plants and trees, e.g., African teak, a valuable source of timber [74]. The African teak tree (*Milicia excelsa*) was among several tree species identified by us at sites visited by tracked bats, along with mango and cashew trees, which are important fruit trees grown in Tanzania [74–76].

Protected areas like Udzungwa Mountains National Park (UMNP) are important for *E. helvum* for both foraging and roosting activities. They provide native sources of food to fruit bats, rather than introduced agricultural fruit species like mangoes. In return, bats can help maintain forest tree composition and propagation in a mutually beneficial relationship [3]. *Eidolon helvum* bats in the Morogoro site were tracked to within 100 m of the Nyandira reserve, part of the Uluguru Mountain range. Both UMNP and the Uluguru mountains are important areas for endemic species of vertebrates and trees in Africa [77], and therefore the seed dispersal services offered by fruit bats in these regions potentially also support the biodiversity contained within them. Forests no longer exist in the Nyandira reserve, due primarily to clearance for new farmland, and it is possible that tracked bats were foraging on human-cultivated plants or trees [78, 79]. The loss of natural food sources due to clearing of native forest and the subsequent shift by bats to feeding on agricultural fruit crops can lead to a conflict between bats and fruit-growers [80]. Perceptions of *E. helvum* shared by community members at our study sites and identified foraging areas/feeding roost locations revealed that bats were disliked and harassed by some community members. Farmers growing mangoes complained of fruit bats negatively impacting their mango harvest, while also being highly-vocal at night and disrupting village households. Some community members drove the bats away with smoke from fires lit under tree roosts or by cutting down the trees themselves. Our findings support the suggestion that preserving natural forests or strategically planting native trees specifically for bat roosts could encourage and draw bats away from fruit crops and human dwellings, thus lessening the potential for bat-human conflict [81].

Camera-traps set up at locations identified by tracking data helped us elucidate species interactions and provide evidence that bats, non-human primates, and other species may have common or shared virus exposure pathways. Due to viral shedding through oral secretions, fruits discarded by bats could serve as vectors for disease transmission to other animals coming into contact with the discarded chewed fruit, e.g. the vervet monkeys pictured handling discarded mangoes [82], and people, especially children who eat discarded fruits from bats because of their sweetness and them having already been spoiled for sale at markets. Similarly, contaminated fecal droppings at foraging and roosting locations could be another route of infection to humans and other species. Bats connecting different landscapes might possibly act as bridge species between relatively undisturbed regions and urban areas, potentially transmitting novel pathogens to other species either directly or indirectly [83]. Currently, there are no known human outbreaks associated with bats in Tanzania, in contrast with bat-associated zoonotic disease outbreaks occuring elsewhere in east-central Africa [84, 85]. However, factors such as roosting in urban colonies (our study sites, [16, 17]), foraging in both urban and protected areas, and the possibility of being transmission hosts of human diseases [32, 33] make tracking *E. helvum* movements important for understanding further exposure pathways and species interactions.

As a pilot study, we successfully explored new ways to collect data that can be used to inform disease surveillance. Increasing the numbers and species of animals tracked, as well as determining differences due to sex, season, or migratory patterns could further help our understanding of bat movement patterns and factors affecting disease transmission and spread between bats and other species [9, 17, 23].

## Conclusions

*Eidolon helvum* bats play an important role in the pollination and seed dispersal of plants within both protected and urban areas in Tanzania; and the conservation of bats and protected areas is thus interdependent. Their movement patterns also suggest the possibility of indirect contact among bats, non-human primates, livestock, and humans in bat foraging areas near human dwellings and in horticulture, as well as an opportunity for virus sharing to occur between bats and other wild species in protected areas. Ensuring that bats have access to wild food sources away from human dwellings or crop plantations should be explored as a potential intervention to mitigate conflict between fruit bats and humans, reduce potential virus sharing, and foster a more amicable coexistence between species.

## Supplementary information

**Additional file 1 Table S1**. Association between the classification of GPS fixes as Flying or Not Flying by different acceleration axes (XYZ, XZ, and YZ). **Figure S1**. Association between the classification of GPS fixes as Flying or Not Flying by different acceleration axes (XYZ, XZ, and YZ).

**Additional file 2 Figure S2**. Plots of acceleration axes readings and associated GPS classification (Flying/Not Flying)

**Additional file 3 Figure S3**. Map of GPS tracks of bats tagged with Argos tags in the Kilombero site.

**Additional file 4 Table S4**. Cumulative distances flown by individual bats on each foraging night. **Figure S4**. Line plot of cumulative distances flown by individual bats on each foraging night.

**Additional file 5 Figure S5**. Heatmap of mean hourly distances (km) flown by bats during individual foraging nights. **Table S5.1.** Mean cumulative hourly distances (km) flown by bats during individual foraging nights. **Table S5.2.** Cumulative hourly distances (km) flown by bats during foraging nights.

**Additional file 6 Figure S6**. Contours of 50 (darker) and 95 (lighter) percent probability of utilization for each bat. The white star represents the tagging location/colony roost site.

**Additional file 7 Figure S7**. Maps of GPS tracks of bats tagged with e-obs loggers in Kilombero. Each map is followed by a map with a nightly breakdown of GPS tracks. The tracks are colored by individual bats, with the larger blue circle corresponding to colony location, the larger red circle corresponding to new day roosts (if any), and foraging/feeding roost areas depicted with yellow circles.

**Additional file 8.** Pictures of camera traps and collected images.

## Data Availability

The GPS unit data analyzed during the current study are available at https://osf.io/m3xks/.

## Abbreviations

CoV: Coronavirus
GPS: Global Positioning System
GUF: Global Urban Footprint
IACUC: Institutional Animal Care and Use Committee
MERS: Middle East respiratory syndrome
SARS: Severe acute respiratory syndrome
SUA: Sokoine University of Agriculture
UMNP: Udzungwa Mountains National Park
WDPA: World Database of Protected Areas

## References

[1] Fujita MS, Tuttle MD. Flying foxes (Chiroptera: Pteropodidae): threatened animals of key ecological and economic importance. Conserv Biol. 1991;5(4):455–63. http://doi.wiley.com/10.1111/j.1523-1739.1991.tb00352.x.

[2] Muscarella R, Fleming TH. The role of frugivorous bats in tropical forest succession. Biol Rev Camb Philos Soc. 2007;82(4):573–90. 10.1111/j.1469-185X.2007.00026.x. 10.1111/j.1469-185X.2007.00026.x 17944618

[3] Kunz TH, Braun de Torrez E, Bauer D, Lobova T, Fleming TH. Ecosystem services provided by bats. Ann N Y Acad Sci. 2011;1223:1–38. 10.1111/j.1749-6632.2011.06004.x. 10.1111/j.1749-6632.2011.06004.x 21449963

[4] Seltzer CE, Ndangalasi HJ, Cordeiro NJ. Seed dispersal in the dark: shedding light on the role of fruit bats in Africa. Biotropica. 2013;45(4):450–6. 10.1111/btp.12029.

[5] Scanlon AT, Petit S, Tuiwawa M, Naikatini A. High similarity between a bat-serviced plant assemblage and that used by humans. Biol Conserv. 2014;174:111–9. 10.1016/j.biocon.2014.03.023.

[6] Nathan R. Long-distance dispersal of plants. Science. 2006;313(5788):786–8. 10.1126/science.1124975. 10.1126/science.1124975 16902126

[7] Damschen EI, Brudvig LA, Haddad NM, Levey DJ, Orrock JL, Tewksbury JJ. The movement ecology and dynamics of plant communities in fragmented landscapes. Proc Natl Acad Sci U S A. 2008;105(49):19078–83. 10.1073/pnas.0802037105. 10.1073/pnas.0802037105 PMC2614718 19060187

[8] Shilton LA, Altringham JD, Compton SG, Whittaker RJ. Old World fruit bats can be long-distance seed dispersers through extended retention of viable seeds in the gut. Proc R Soc Lond Ser B Biol Sci. 1999. 10.1098/rspb.1999.0625.

[9] Richter HV, Cumming GS. Food availability and annual migration of the straw-colored fruit bat *Eidolon helvum*. J Zool. 2006;268(1):35–44. 10.1111/j.1469-7998.2005.00020.x.

[10] Tsoar A, Nathan R, Bartan Y, Vyssotski A, Dell’Omo G, Ulanovsky N. Large-scale navigational map in a mammal. Proc Natl Acad Sci U S A. 2011;108(37):E718–24. 10.1073/pnas.1107365108. 10.1073/pnas.1107365108 PMC3174628 21844350

[11] Foley JA, Defries R, Asner GP, Barford C, Bonan G, Carpenter SR, et al. Global consequences of land use. Science. 2005;309(5734):570–4. 10.1126/science.1111772. 10.1126/science.1111772 16040698

[12] Mayaux P, Holmgren P, Achard F, Eva H, Stibig H-J, Branthomme A. Tropical forest cover change in the 1990s and options for future monitoring. Philos Trans R Soc Lond Ser B Biol Sci. 2005;360(1454):373–84. 10.1098/rstb.2004.1590. 10.1098/rstb.2004.1590 PMC1569459 15814351

[13] Bacles CFE, Lowe AJ, Ennos RA. Effective seed dispersal across a fragmented landscape. Science. 2006;311(5761):628. 10.1126/science.1121543. 10.1126/science.1121543 16456072

[14] Cordeiro NJ, Howe HF. Forest fragmentation severs mutualism between seed dispersers and an endemic African tree. Proc Natl Acad Sci U S A. 2003;100(24):14052–6. 10.1073/pnas.2331023100. 10.1073/pnas.2331023100 PMC283544 14614145

[15] Corlett RT. Frugivory and seed dispersal by vertebrates in tropical and subtropical Asia: an update. Global Ecology and Conservation. 2017;11:1–22. 10.1016/j.gecco.2017.04.007.

[16] Abedi-Lartey M, Dechmann DKN, Wikelski M, Scharf AK, Fahr J. Long-distance seed dispersal by straw-coloured fruit bats varies by season and landscape. Global Ecology and Conservation. 2016;7:12–24. 10.1016/j.gecco.2016.03.005.

[17] Fahr J, Abedi-Lartey M, Esch T, Machwitz M, Suu-Ire R, Wikelski M, et al. Pronounced seasonal changes in the movement ecology of a highly gregarious central-place forager, the African straw-coloured fruit bat (*Eidolon helvum*). PLoS One. 2015;10(10):e0138985. 10.1371/journal.pone.0138985. 10.1371/journal.pone.0138985 PMC4605647 26465139

[18] van Toor ML, O’Mara MT, Abedi-Lartey M, Wikelski M, Fahr J, Dechmann DKN. Linking colony size with quantitative estimates of ecosystem services of African fruit bats. Curr Biol. 2019;29(7):R237–8. 10.1016/j.cub.2019.02.033. 10.1016/j.cub.2019.02.033 30939302

[19] Thomas DW. The annual migrations of three species of West African fruit bats (Chiroptera: Pteropodidae). Can J Zool. 1983;61(10):2266–72. 10.1139/z83-299.

[20] Hayman DTS, McCrea R, Restif O, Suu-Ire R, Fooks AR, Wood JLN, et al. Demography of straw-colored fruit bats in Ghana. J Mammal. 2012;93(5):1393–404. 10.1644/11-MAMM-A-270.1. 10.1644/11-MAMM-A-270.1 PMC3605799 23525358

[21] Webala PW, Musila S, Makau R. Roost occupancy, roost site selection and diet of straw-coloured fruit bats (Pteropodidae: *Eidolon helvum*) in western Kenya: the need for continued public education. Acta Chiropt. 2014;16(1):85–94. 10.3161/150811014X683291.

[22] Peel AJ, Wood JLN, Baker KS, Breed AC, Carvalho AD, Fernández-Loras A, et al. How does Africa’s most hunted bat vary across the continent? Population traits of the straw-coloured fruit bat (*Eidolon helvum*) and its interactions with humans. Acta Chiropt. 2017;19(1):77–92. 10.3161/15081109ACC2017.19.1.006.

[23] Richter HV, Cumming GS. First application of satellite telemetry to track African straw-coloured fruit bat migration. J Zool. 2008;275(2):172–6. 10.1111/j.1469-7998.2008.00425.x.

[24] Amman BR, Carroll SA, Reed ZD, Sealy TK, Balinandi S, Swanepoel R, et al. Seasonal pulses of Marburg virus circulation in juvenile *Rousettus aegyptiacus* bats coincide with periods of increased risk of human infection. PLoS Pathog. 2012;8(10):e1002877. 10.1371/journal.ppat.1002877. 10.1371/journal.ppat.1002877 PMC3464226 23055920

[25] Towner JS, Amman BR, Sealy TK, Carroll SAR, Comer JA, Kemp A, et al. Isolation of genetically diverse Marburg viruses from Egyptian fruit bats. PLoS Pathog. 2009;5(7):e1000536. 10.1371/journal.ppat.1000536. 10.1371/journal.ppat.1000536 PMC2713404 19649327

[26] Ogawa H, Miyamoto H, Nakayama E, Yoshida R, Nakamura I, Sawa H, et al. Seroepidemiological prevalence of multiple species of filoviruses in fruit bats (*Eidolon helvum*) migrating in Africa. J Infect Dis. 2015;212(Suppl 2):S101–8. 10.1093/infdis/jiv063. 10.1093/infdis/jiv063 25786916

[27] Shears P, O’Dempsey TJD. Ebola virus disease in Africa: epidemiology and nosocomial transmission. J Hosp Infect. 2015;90(1):1–9. 10.1016/j.jhin.2015.01.002. 10.1016/j.jhin.2015.01.002 25655197

[28] Goldstein T, Anthony SJ, Gbakima A, Bird BH, Bangura J, Tremeau-Bravard A, et al. The discovery of Bombali virus adds further support for bats as hosts of ebolaviruses. Nat Microbiol. 2018;3(10):1084–9. 10.1038/s41564-018-0227-2. 10.1038/s41564-018-0227-2 PMC6557442 30150734

[29] Thanapongtharm W, Linard C, Wiriyarat W, Chinsorn P, Kanchanasaka B, Xiao X, et al. Spatial characterization of colonies of the flying fox bat, a carrier of Nipah virus in Thailand. BMC Vet Res. 2015;11:81. 10.1186/s12917-015-0390-0. 10.1186/s12917-015-0390-0 PMC4389713 25880385

[30] Daszak P, Zambrana-Torrelio C, Bogich TL, Fernandez M, Epstein JH, Murray KA, et al. Interdisciplinary approaches to understanding disease emergence: the past, present, and future drivers of Nipah virus emergence. Proc Natl Acad Sci U S A. 2013;110(Suppl 1):3681–8. 10.1073/pnas.1201243109. 10.1073/pnas.1201243109 PMC3586606 22936052

[31] Drexler JF, Corman VM, Gloza-Rausch F, Seebens A, Annan A, Ipsen A, et al. Henipavirus RNA in African bats. PLoS One. 2009;4(7):e6367. 10.1371/journal.pone.0006367. 10.1371/journal.pone.0006367 PMC2712088 19636378

[32] Baker KS, Todd S, Marsh GA, Crameri G, Barr J, Kamins AO, et al. Novel, potentially zoonotic paramyxoviruses from the African straw-colored fruit bat *Eidolon helvum*. J Virol. 2013;87(3):1348–58. 10.1128/JVI.01202-12. 10.1128/JVI.01202-12 PMC3554137 23152534

[33] Anthony SJ, Johnson CK, Greig DJ, Kramer S, Che X, Wells H, et al. Global patterns in coronavirus diversity. Virus Evol. 2017;3(1):vex012. 10.1093/ve/vex012. 10.1093/ve/vex012 PMC5467638 28630747

[34] Li W, Shi Z, Yu M, Ren W, Smith C, Epstein JH, et al. Bats are natural reservoirs of SARS-like coronaviruses. Science. 2005;310(5748):676–9. 10.1126/science.1118391. 10.1126/science.1118391 16195424

[35] Anthony SJ, Gilardi K, Menachery VD, Goldstein T, Ssebide B, Mbabazi R, et al. Further evidence for bats as the evolutionary source of Middle East respiratory syndrome coronavirus. MBio. 2017;8(2). 10.1128/mBio.00373-17. 10.1128/mBio.00373-17 PMC5380844 28377531

[36] Ge X-Y, Li J-L, Yang X-L, Chmura AA, Zhu G, Epstein JH, et al. Isolation and characterization of a bat SARS-like coronavirus that uses the ACE2 receptor. Nature. 2013;503(7477):535–8. 10.1038/nature12711. 10.1038/nature12711 PMC5389864 24172901

[37] Han H-J, Wen H-L, Zhou C-M, Chen F-F, Luo L-M, Liu J-W, et al. Bats as reservoirs of severe emerging infectious diseases. Virus Res. 2015;205:1–6. 10.1016/j.virusres.2015.05.006. 10.1016/j.virusres.2015.05.006 PMC7132474 25997928

[38] Kunz TH. Ecology of bats: Springer Science & Business Media; 2013.

[39] Teeling EC, Springer MS, Madsen O, Bates P, O’brien SJ, Murphy WJ. A molecular phylogeny for bats illuminates biogeography and the fossil record. Science. 2005;307(5709):580–4. 10.1126/science.1105113. 10.1126/science.1105113 15681385

[40] Wilson DE, Reeder DM. Mammal species of the world: a taxonomic and geographic reference: JHU Press; 2005.

[41] Jepsen GL. Early Eocene bat from Wyoming. Science. 1966;154(3754):1333–9. 10.1126/science.154.3754.1333. 10.1126/science.154.3754.1333 17770307

[42] Drexler JF, Corman VM, Wegner T, Tateno AF, Zerbinati RM, Gloza-Rausch F, et al. Amplification of emerging viruses in a bat colony. Emerg Infect Dis. 2011;17(3):449–56. 10.3201/eid1703.100526. 10.3201/eid1703.100526 PMC3165994 21392436

[43] O’Shea TJ, Cryan PM, Cunningham AA, Fooks AR, Hayman DTS, Luis AD, et al. Bat flight and zoonotic viruses. Emerg Infect Dis. 2014;20(5):741–5. 10.3201/eid2005.130539. 10.3201/eid2005.130539 PMC4012789 24750692

[44] Dobson AP. What links bats to emerging infectious diseases? Science. 2005;310(5748):628–9. 10.1126/science.1120872. 10.1126/science.1120872 16254175

[45] Calisher CH, Childs JE, Field HE, Holmes KV, Schountz T. Bats: important reservoir hosts of emerging viruses. Clin Microbiol Rev. 2006;19(3):531–45. 10.1128/CMR.00017-06. 10.1128/CMR.00017-06 PMC1539106 16847084

[46] Peel AJ, Sargan DR, Baker KS, Hayman DTS, Barr JA, Crameri G, et al. Continent-wide panmixia of an African fruit bat facilitates transmission of potentially zoonotic viruses. Nat Commun. 2013;4:2770. 10.1038/ncomms3770. 10.1038/ncomms3770 PMC3836177 24253424

[47] Helbig-Bonitz M, Rutten G, Kalko EKV. Fruit bats can disperse figs over different land-use types on Mount Kilimanjaro, Tanzania. Afr J Ecol. 2014;52(1):122–5. 10.1111/aje.12090.

[48] Hayman DTS, Peel AJ. Can survival analyses detect hunting pressure in a highly connected species? Lessons from straw-coloured fruit bats. Biol Conserv. 2016;200:131–9. 10.1016/j.biocon.2016.06.003. 10.1016/j.biocon.2016.06.003 PMC4965785 27499548

[49] National Bureau of Statistics (2012). Tanzania population and housing census.

[50] Sikes RS, Gannon WL, Animal Care and Use Committee of the American Society of Mammalogists. Guidelines of the American Society of Mammalogists for the use of wild mammals in research. J Mammal. 2011;92(1):235–53. 10.1644/10-MAMM-F-355.1. 10.1093/jmammal/gyw078 PMC5909806 29692469

[51] O’Mara MT, Wikelski M, Dechmann DKN. 50 years of bat tracking: device attachment and future directions. Methods Ecol Evol. 2014;5(4):311–9. 10.1111/2041-210X.12172.

[52] Calenge C (2006). The package adehabitat for the R software: tool for the analysis of space and habitat use by animals. Ecol Model.

[53] GRASS Development team. Geographic Resources Analysis Support System (GRASS) Software. Open Source Geospatial Foundation. 2018. Available from: https://grass.osgeo.org.

[54] Esch T, Heldens W, Hirner A, Keil M, Marconcini M, Roth A, et al. Breaking new ground in mapping human settlements from space – the Global Urban Footprint. ISPRS J Photogramm Remote Sens. 2017;134:3042. 10.1016/j.isprsjprs.2017.10.012.

[55] Esch T, Schenk A, Ullmann T, Thiel M, Roth A, Dech S. Characterization of land cover types in TerraSAR-X images by combined analysis of speckle statistics and intensity information. IEEE Trans Geosci Remote Sens. 2011;49(6):1911–25. 10.1109/TGRS.2010.2091644.

[56] UNEP-WCMC. Protected area profile for United Republic of Tanzania from the world database of protected areas; 2018. Available from: www.protectedplanet.net.

[57] R Development Core Team. R: a language and environment for statistical computing. Vienna: R Foundation for statistical Computing; 2008. Available from: http://www.R-project.org.

[58] Kahle D, Wickham H. Ggmap: spatial visualization with ggplot2. The R Journal. 2013;5(1):144–61 Available from: https://journal.r-project.org/archive/2013-1/kahle-wickham.pdf.

[59] Harrison E, Drake T, Ots R. Finalfit: quickly create elegant regression results tables and plots when modelling; 2020. Available from: https://CRAN.R-project.org/package=finalfit.

[60] Dowle M, Srinivasan A. Data.Table: extension of ‘data.frame’; 2019. Available from: https://CRAN.R-project.org/package=data.table.

[61] Kranstauber B, Smolla M, Scharf AK. Move: visualizing and analyzing animal track data; 2019. Available from: https://CRAN.R-project.org/package=move.

[62] Wickham H, François R, Henry L, Müller K. “Dplyr”: a grammar of data manipulation; 2020. Available from: https://CRAN.R-project.org/package=dplyr.

[63] Bivand R, Lewin-Koh N. Maptools: tools for handling spatial objects; 2019. Available from: https://CRAN.R-project.org/package=maptools.

[64] Grolemund G, Wickham H. Dates and times made easy with lubridate. J Stat Softw. 2011;40(3):1–25 Available from: http://www.jstatsoft.org/v40/i03.

[65] Xie Y. Knitr: a general-purpose package for dynamic report generation in R; 2020. Available from: https://yihui.org/knitr.

[66] Wickham H, Henry L. Tidyr: tidy messy data; 2020. Available from: https://CRAN.R-project.org/package=tidyr.

[67] Wilke CO. Cowplot: streamlined plot theme and plot annotations for ‘ggplot2’; 2019. Available from: https://CRAN.R-project.org/package=cowplot.

[68] Rudis B. Hrbrthemes: additional themes, theme components and utilities for ‘ggplot2’; 2019. Available from: https://CRAN.R-project.org/package=hrbrthemes.

[69] Pebesma E. Simple features for R: standardized support for spatial vector data. The R Journal. 2018;10(1):439–46. 10.32614/RJ-2018-009.

[70] Santos BO. Ggsn: north symbols and scale bars for maps created with ‘ggplot2’ or ‘ggmap’; 2019. Available from: https://CRAN.R-project.org/package=ggsn.

[71] Zhu H. kableExtra: construct complex table with ‘kable’ and pipe syntax. 2019; Available from: https://CRAN.R-project.org/package=kableExtra.

[72] Bivand R, Keitt T, Rowlingson B. Rgdal: bindings for the ‘geospatial’ data abstraction library; 2019. Available from: https://CRAN.R-project.org/package=rgdal.

[73] Bivand R, Rundel C. Rgeos: Interface to geometry engine - open source (‘geos’); 2019. Available from: https://CRAN.R-project.org/package=rgeos.

[74] Daïnou K, Laurenty E, Mahy G, Hardy OJ, Brostaux Y, Tagg N, et al. Phenological patterns in a natural population of a tropical timber tree species, *Milicia excelsa* (Moraceae): evidence of isolation by time and its interaction with feeding strategies of dispersers. Am J Bot. 2012;99(9):1453–63. 10.3732/ajb.1200147. 10.3732/ajb.1200147 22912370

[75] Mitchell D (2004). Tanzania’s cashew sector: constraints and challenges in a global environment.

[76] Msogoya JT, Kimaro SE (2011). Assessment and management of post-harvest losses of fresh mango under small-scale business in Morogoro, Tanzania. Journal of Animal & Plant Sciences.

[77] Burgess ND, Butynski TM, Cordeiro NJ, Doggart NH, Fjeldså J, Howell KM, et al. The biological importance of the Eastern Arc Mountains of Tanzania and Kenya. Biol Conserv. 2007;134(2):209–31. 10.1016/j.biocon.2006.08.015.

[78] Doggart N, Lovett J, Mhoro B, Kiure J, Burgess N. Biodiversity surveys in the forest reserves of the Uluguru Mountains. Technical paper for The Wildlife Conservation Society of Tanzania. 2004. Available from: http://www.easternarc.or.tz/groups/webcontent/documents/pdf/Ulugurus%20draft%20Part%201%20v.5.pdf.

[79] Burgess N, Doggart N, Lovett JC. The Uluguru Mountains of eastern Tanzania: the effect of forest loss on biodiversity. Oryx. 2002;36(2):140–52. 10.1017/S0030605302000212.

[80] Aziz SA, Olival KJ, Bumrungsri S, Richards GC, Racey PA (2016). The conflict between pteropodid bats and fruit growers: species, legislation and mitigation. Bats in the anthropocene: conservation of bats in a changing world.

[81] Law B, Eby P, Somerville D. Tree-planting to conserve flying-foxes and reduce orchard damage. Managing the grey-headed flying-fox as a threatened species. Sydney: Royal Zoological Society of New South Wales; 2002. p. 84–94. Available from: https://publications.rzsnsw.org.au/doi/pdf/10.7882/FS.2002.041.

[82] Amman BR, Jones MEB, Sealy TK, Uebelhoer LS, Schuh AJ, Bird BH, et al. Oral shedding of Marburg virus in experimentally infected Egyptian fruit bats (*Rousettus aegyptiacus*). J Wildl Dis. 2015;51(1):113–24. 10.7589/2014-08-198. 10.7589/2014-08-198 PMC5022530 25375951

[83] Caron A, Cappelle J, Cumming GS, de Garine-Wichatitsky M, Gaidet N. Bridge hosts, a missing link for disease ecology in multi-host systems. Vet Res. 2015;46:83. 10.1186/s13567-015-0217-9. 10.1186/s13567-015-0217-9 PMC4509689 26198845

[84] Towner JS, Pourrut X, Albariño CG, Nkogue CN, Bird BH, Grard G, et al. Marburg virus infection detected in a common African bat. PLoS One. 2007;2(8):e764. 10.1371/journal.pone.0000764. 10.1371/journal.pone.0000764 PMC1942080 17712412

[85] Pigott DM, Golding N, Mylne A, Huang Z, Weiss DJ, Brady OJ, et al. Mapping the zoonotic niche of Marburg virus disease in Africa. Trans R Soc Trop Med Hyg. 2015;109(6):366–78. 10.1093/trstmh/trv024. 10.1093/trstmh/trv024 PMC4447827 25820266

