# Supplementary material for: Fruit bats in flight: a look into the movements of the ecologically important Eidolon helvum in Tanzania
Source: One Health Outlook. 2020 Aug 5;2:16. doi: 10.1186/s42522-020-00020-9 (PMC7402849; doi:10.1186/s42522-020-00020-9)

**Additional file 1**

**Comparing classification of GPS points using different acceleration axes.**

**Table S1: Association between the classification of GPS fixes as Flying or Not Flying by different acceleration axes (XYZ, XZ, and YZ).**

| **Dependent: Flying** |  | **Not Flying**  **Mean(SD)** | **Flying**  **Mean(SD)** | **p** |
| --- | --- | --- | --- | --- |
| axis | xyz | 11207 (33.4) | 20485 (33.3) | 0.735 |
|  | xz | 11200 (33.4) | 20492 (33.3) |  |
|  | yz | 11122 (33.2) | 20570 (33.4) |  |
| tagID | K5309 | 9380 (28.0) | 18256 (29.7) | <0.001 |
|  | K5310 | 6093 (18.2) | 26325 (42.8) |  |
|  | K5311 | 1823 (5.4) | 3217 (5.2) |  |
|  | K5312 | 3264 (9.7) | 2667 (4.3) |  |
|  | K5313 | 6171 (18.4) | 5241 (8.5) |  |
|  | K5315 | 984 (2.9) | 36 (0.1) |  |
|  | K5317 | 3597 (10.7) | 4941 (8.0) |  |
|  | K5319 | 2217 (6.6) | 864 (1.4) |  |

**Figure S1: Association between the classification of GPS fixes as Flying or Not Flying by different acceleration axes (XYZ, XZ, and YZ).**


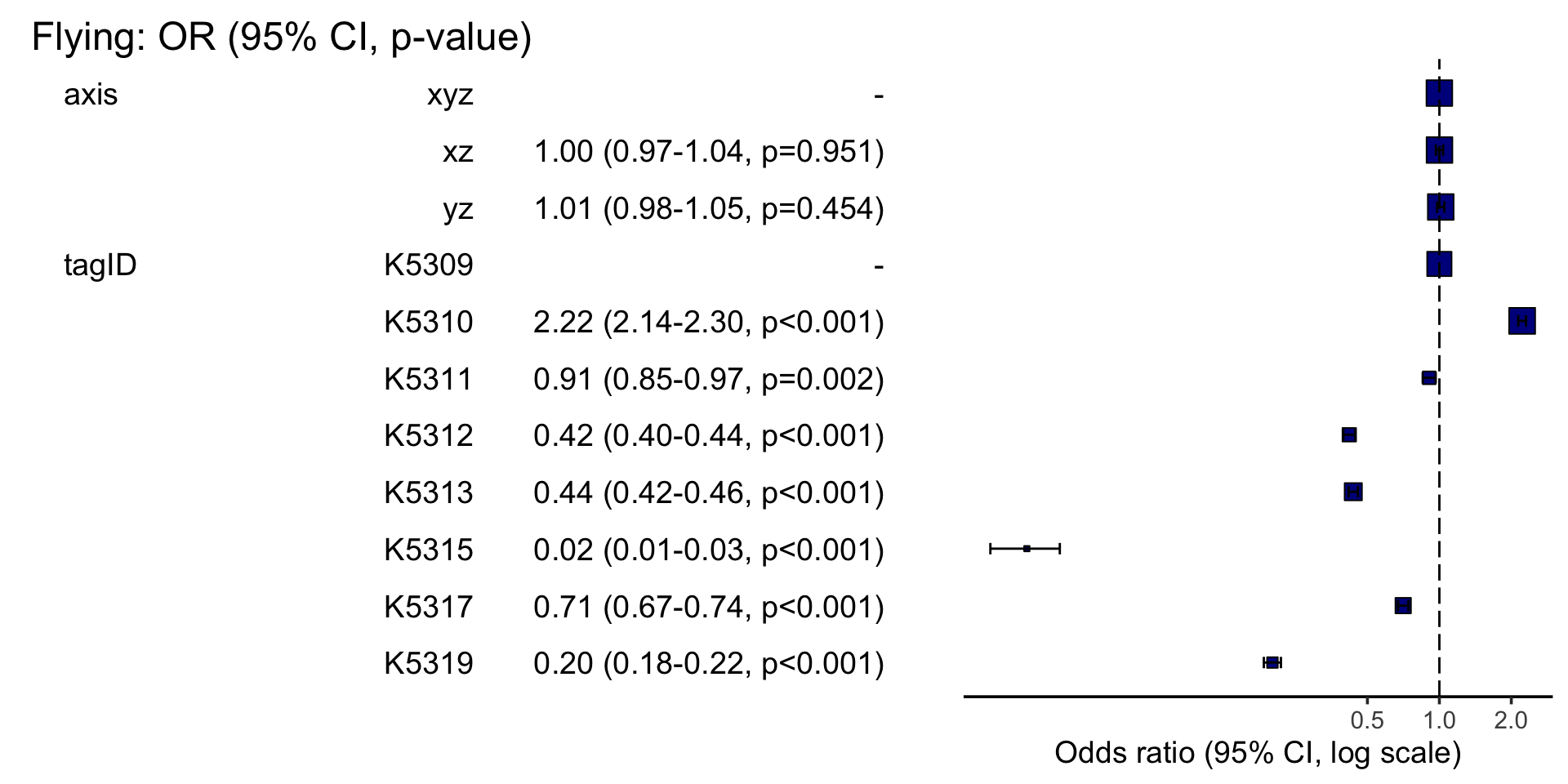


**Additional figures showing the classification of GPS fixes by different axes, for each bat.**


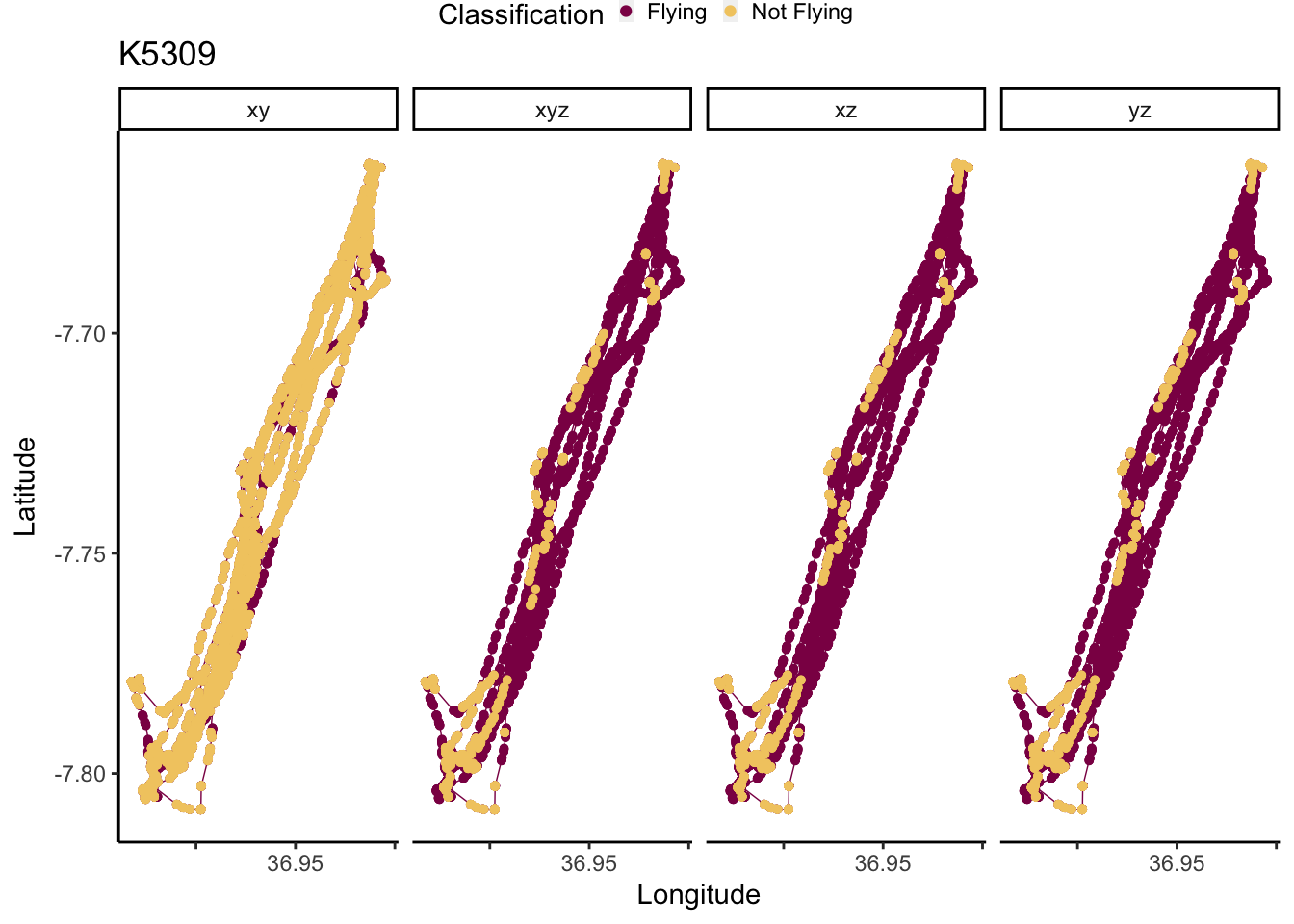


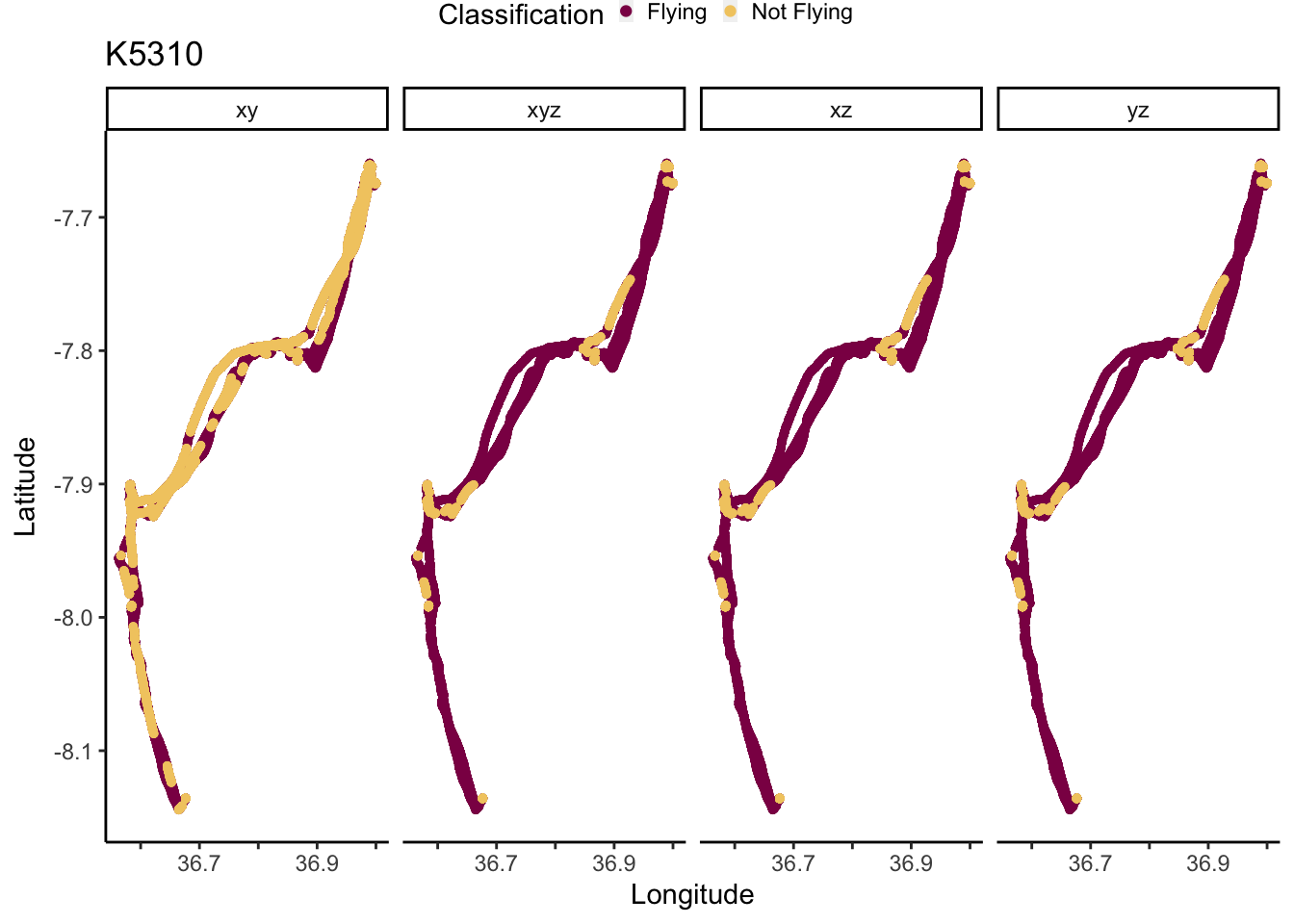


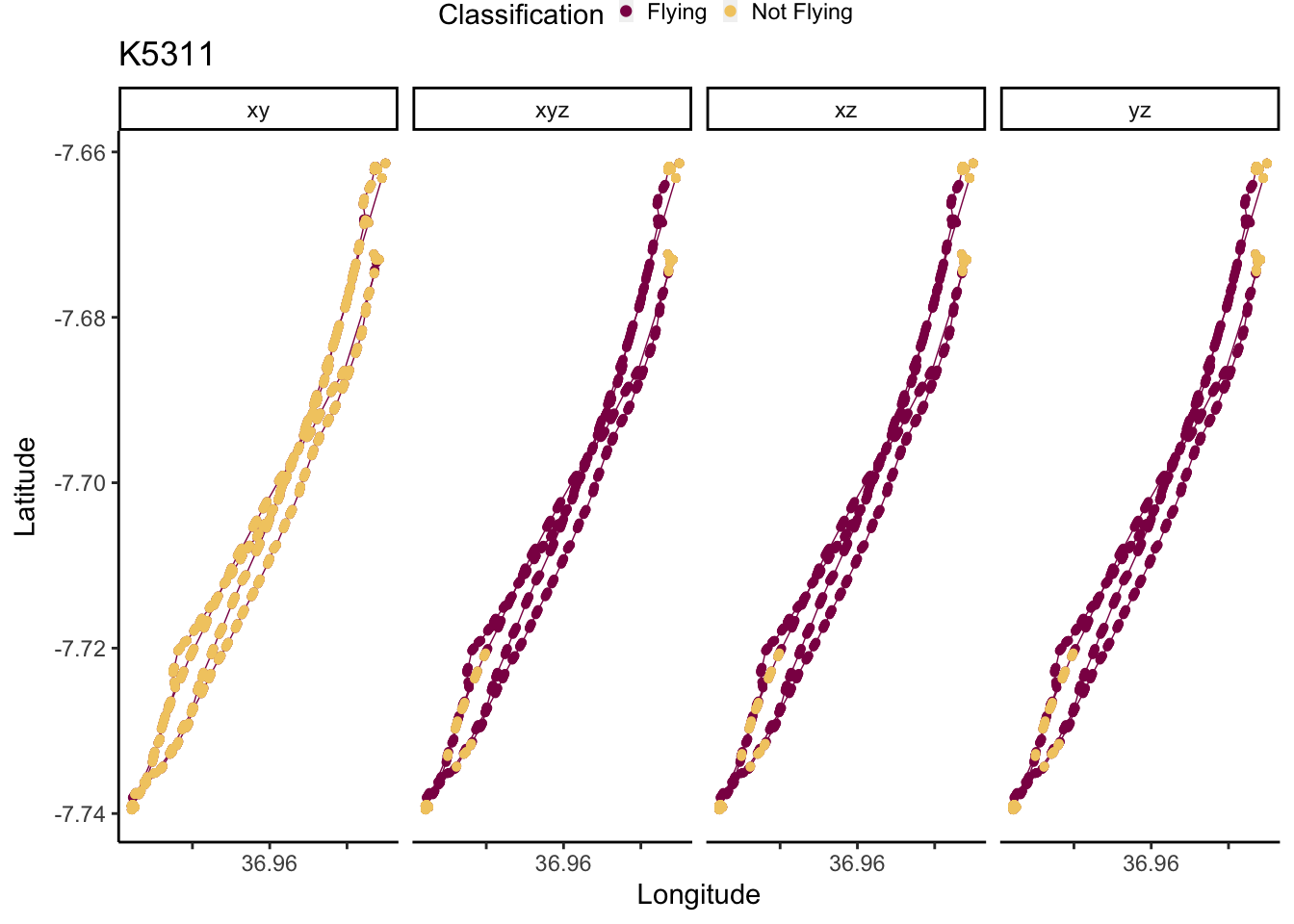


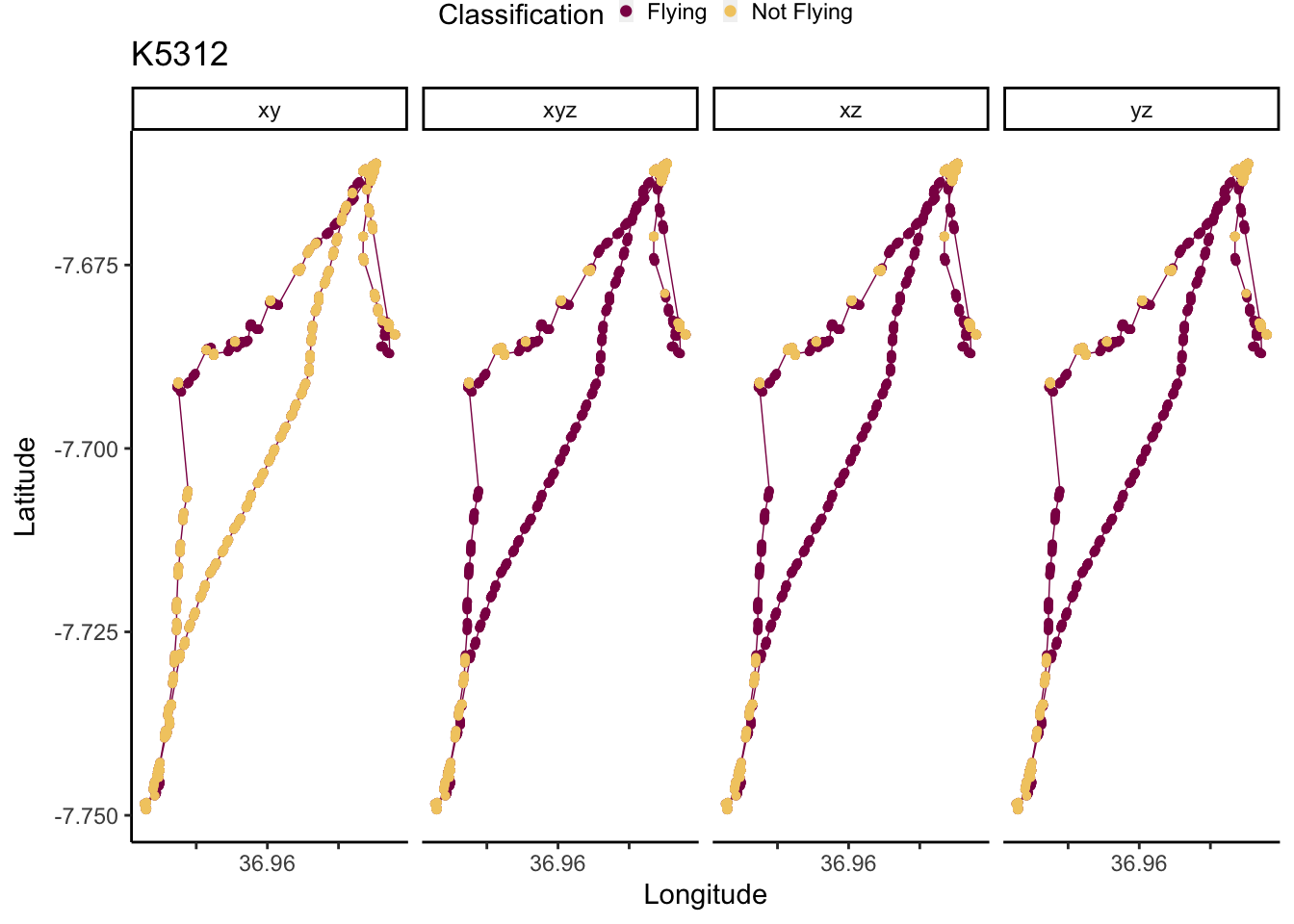


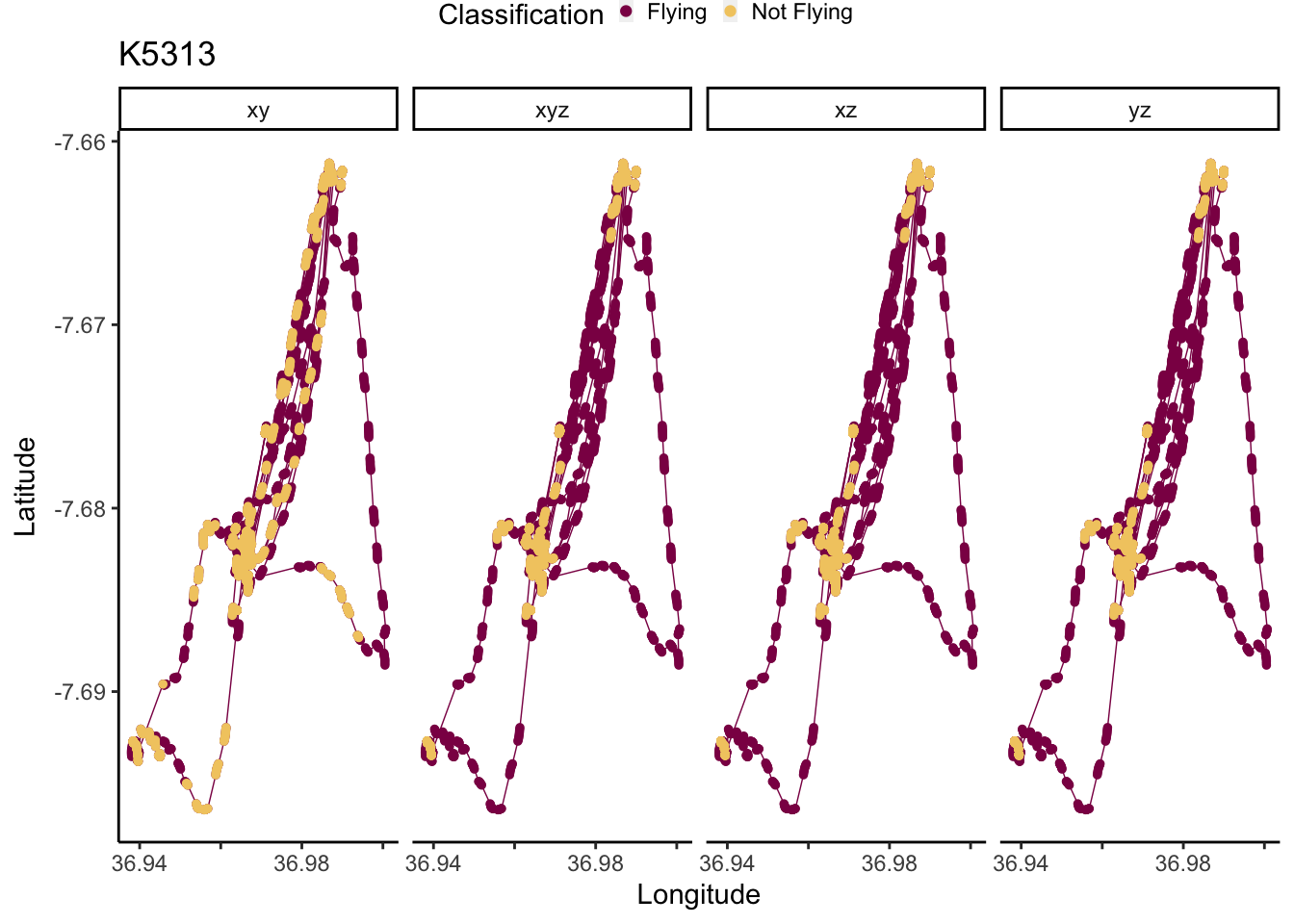


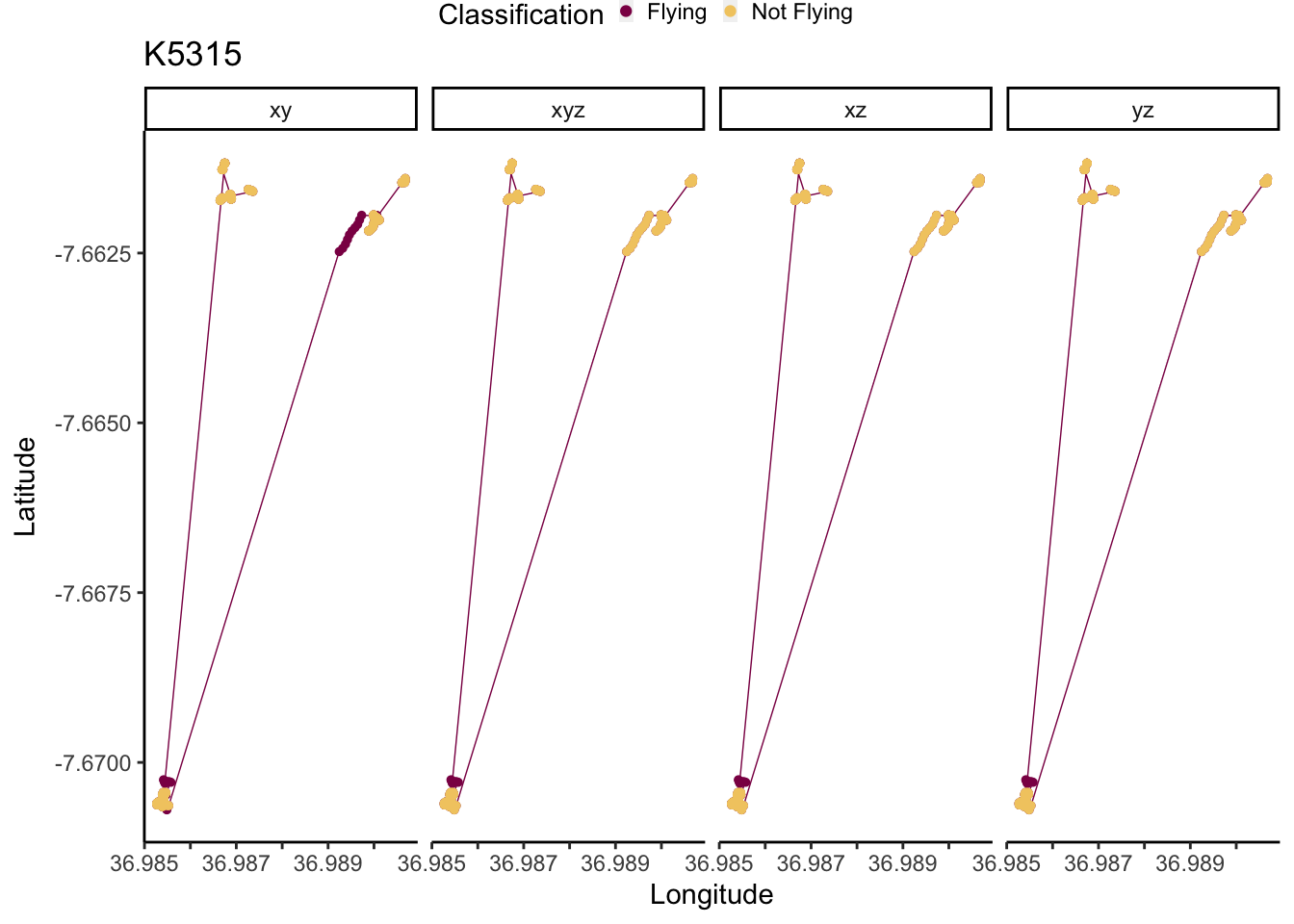


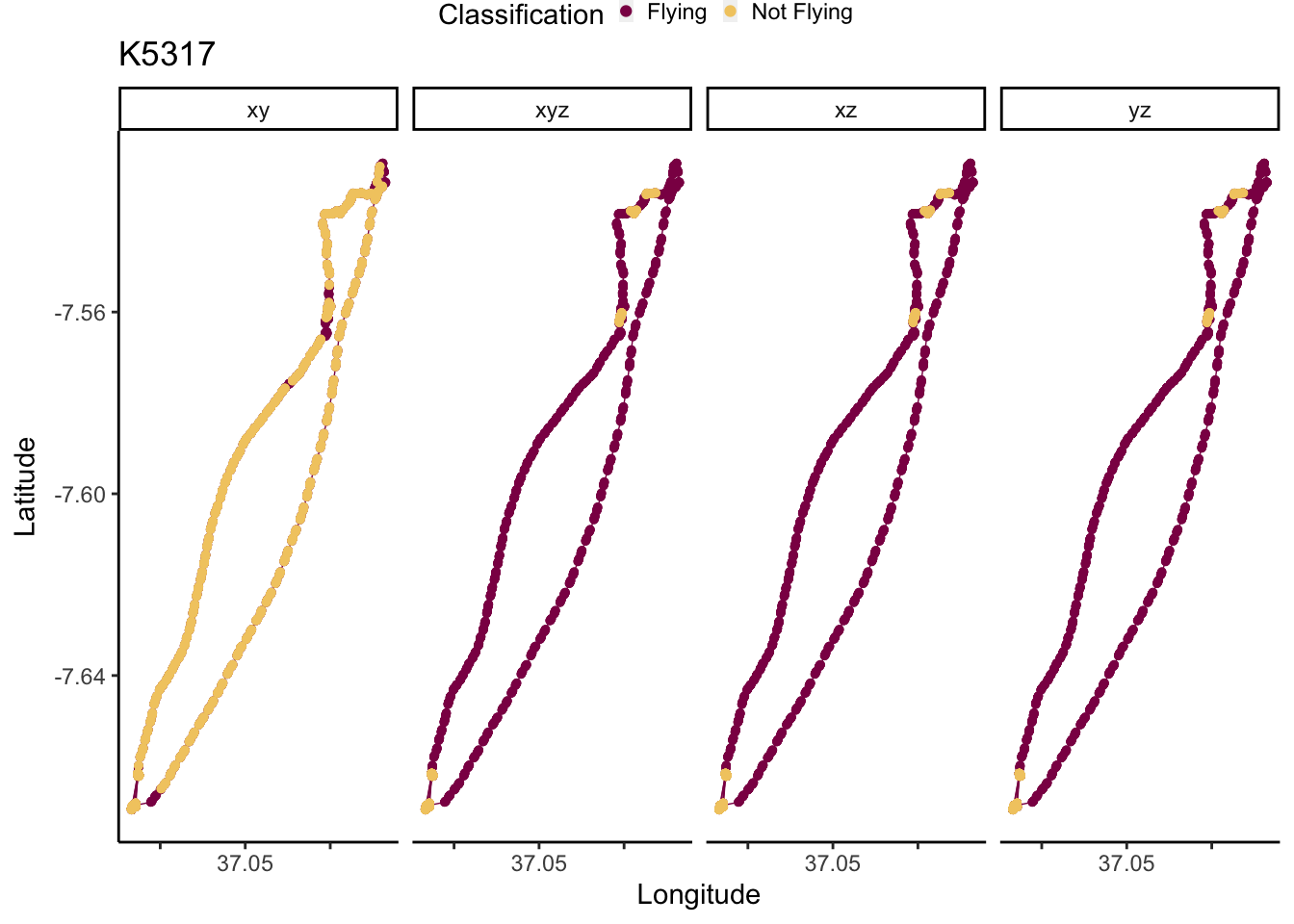


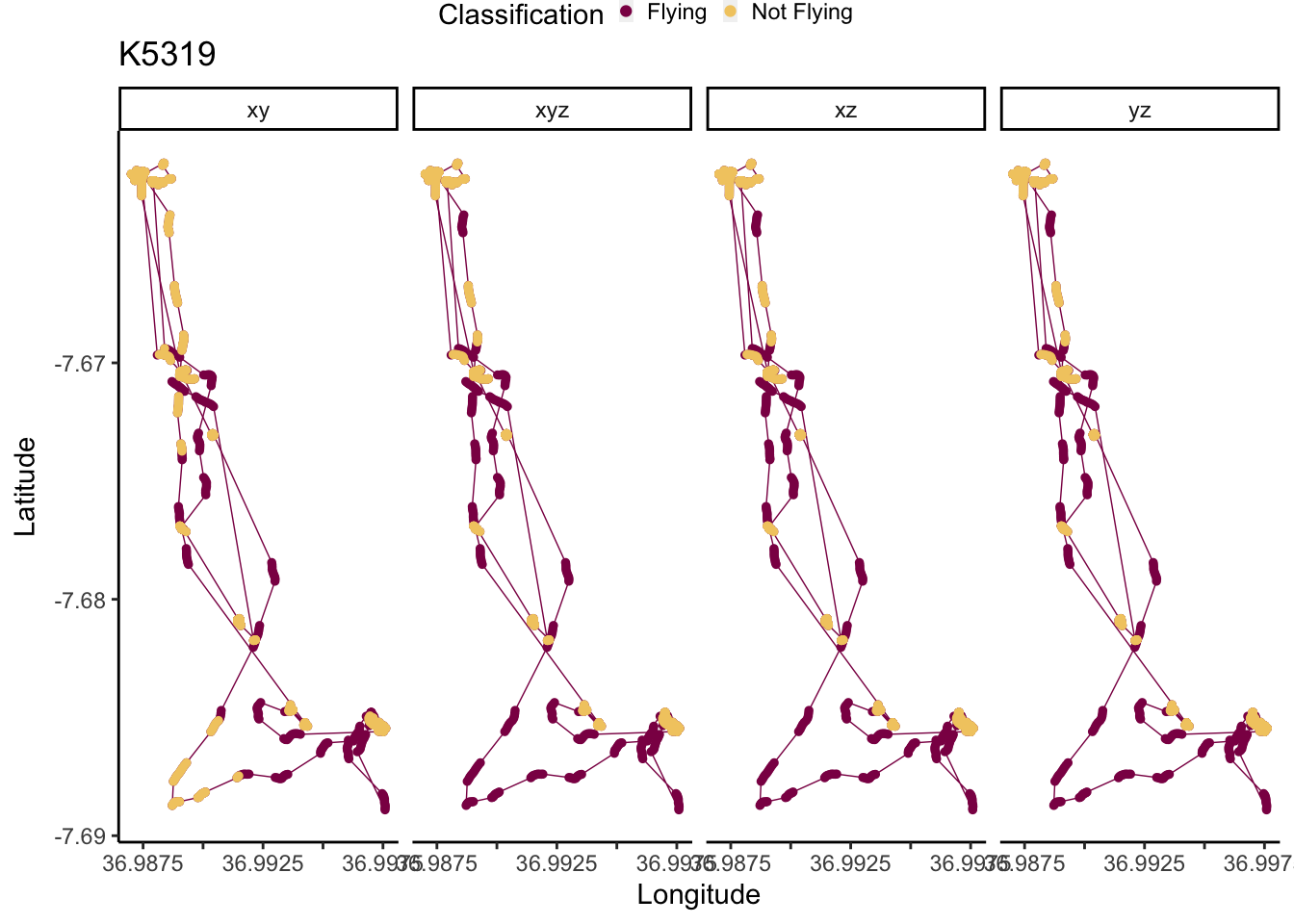

Supplement: Supplementary file 1 — Additional file 1 Table S1. Association between the classification of GPS fixes as Flying or Not Flying by different acceleration axes (XYZ, XZ, and YZ). Figure S1. Association between the classification of GPS fixes as Flying or Not Flying by different acceleration axes (XYZ, XZ, and YZ). [file 42522_2020_20_MOESM1_ESM.docx]
