# Supplementary material for: Fruit bats in flight: a look into the movements of the ecologically important Eidolon helvum in Tanzania
Source: One Health Outlook. 2020 Aug 5;2:16. doi: 10.1186/s42522-020-00020-9 (PMC7402849; doi:10.1186/s42522-020-00020-9)
Supplement: Supplementary file 2 — Additional file 2 Figure S2. Plots of acceleration axes readings and associated GPS classification (Flying/Not Flying) [file 42522_2020_20_MOESM2_ESM.docx]

**Additional file 2**

**Figure S2. Plots of acceleration axes readings and associated GPS classification (Flying/Not Flying)
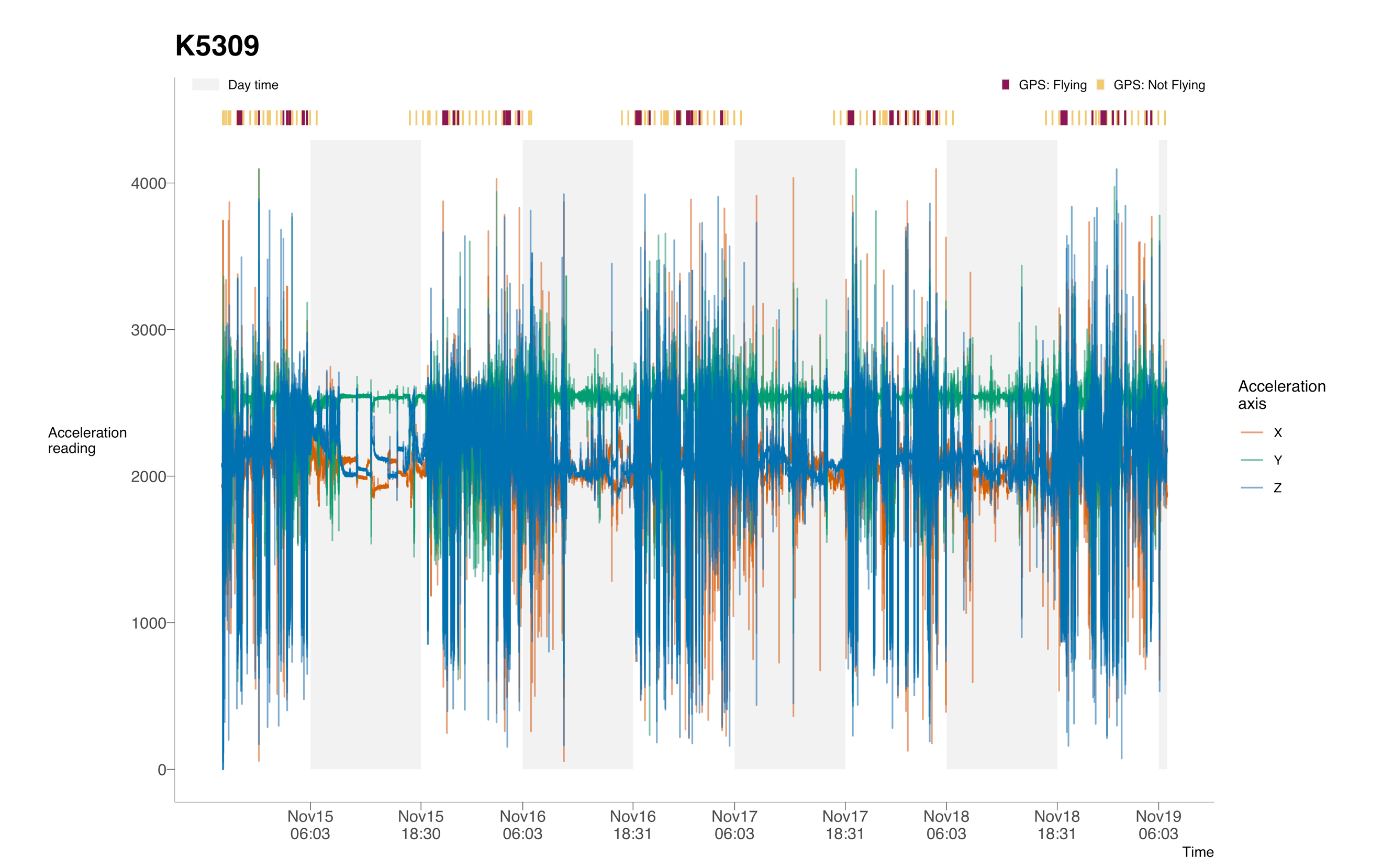

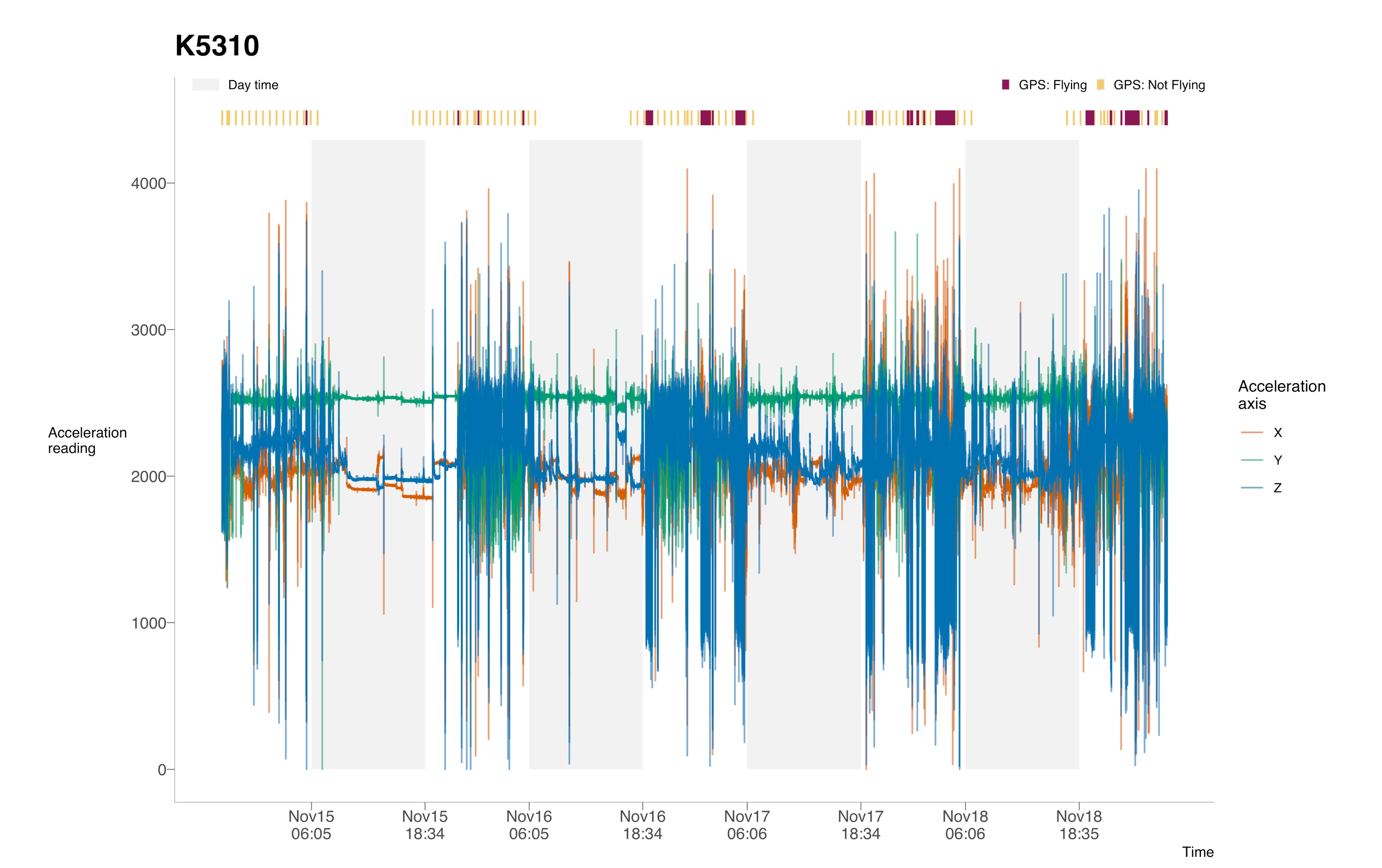

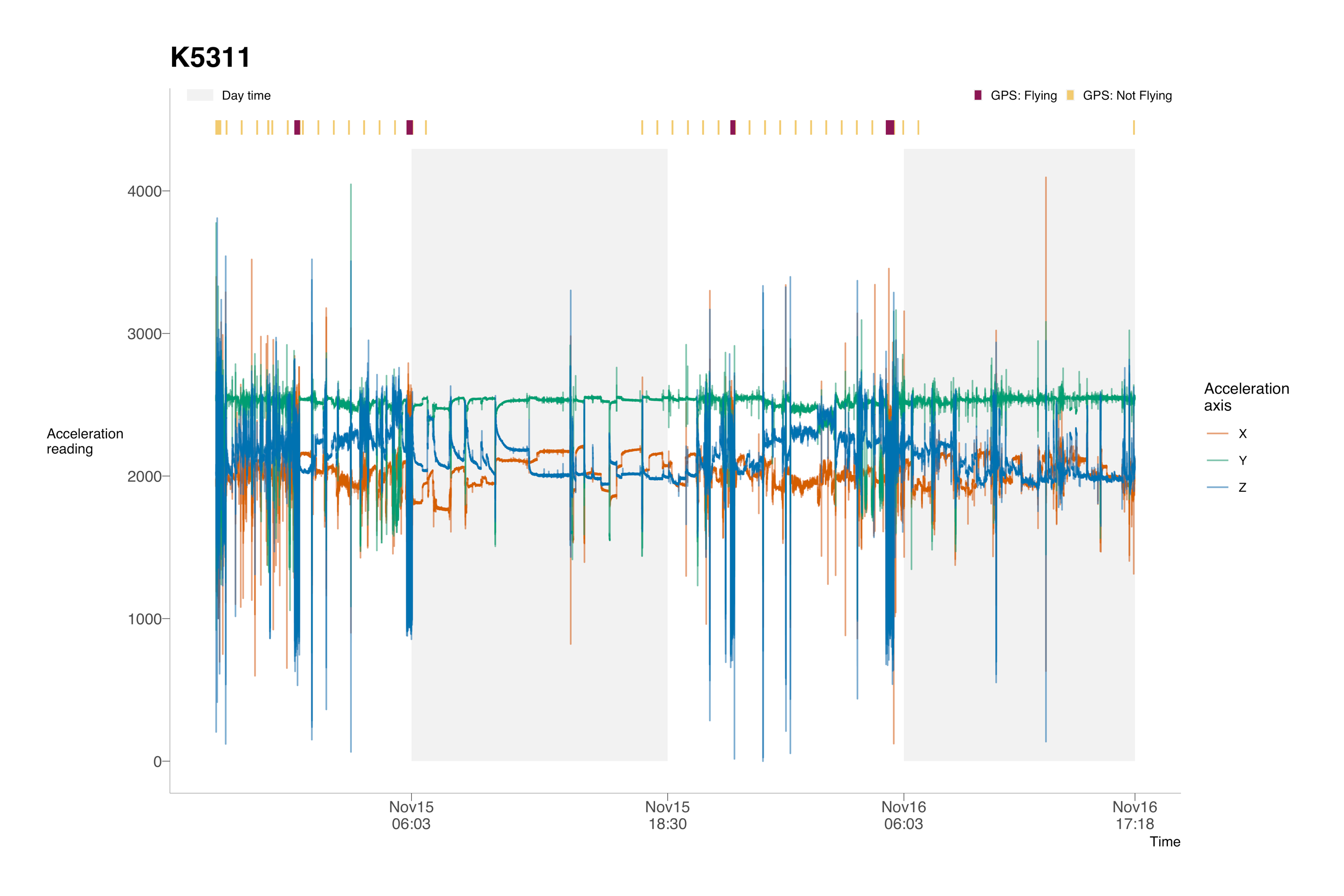

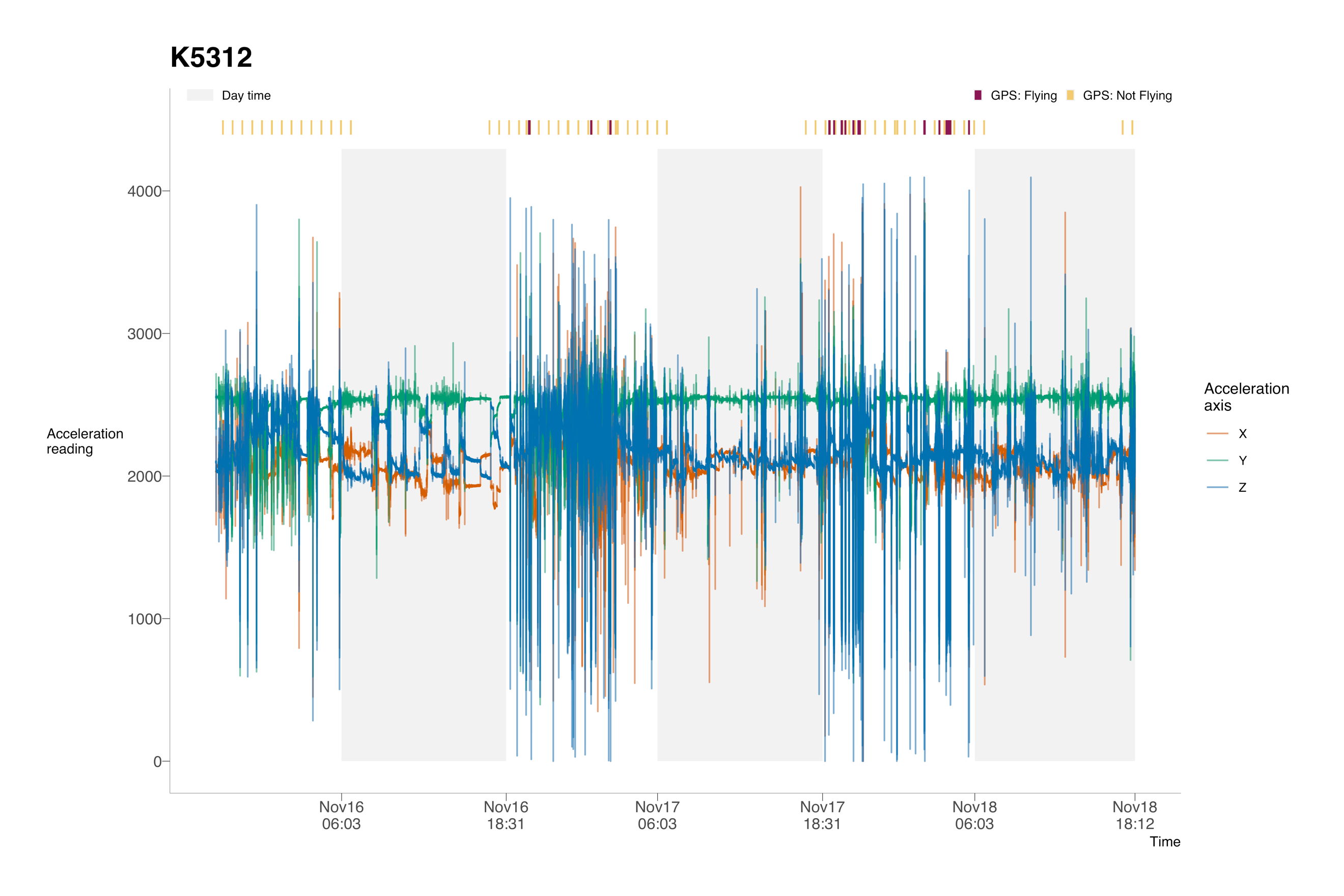

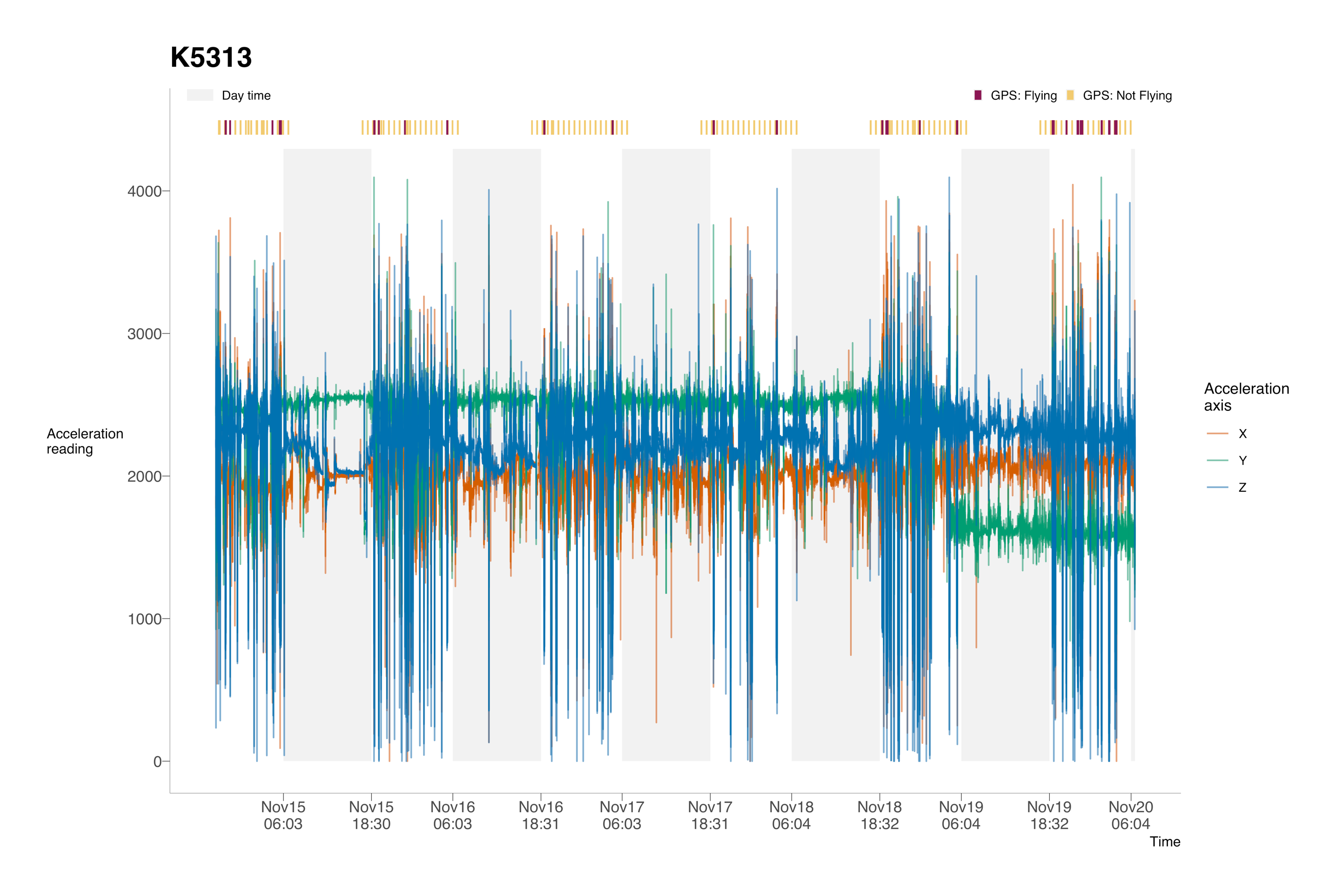

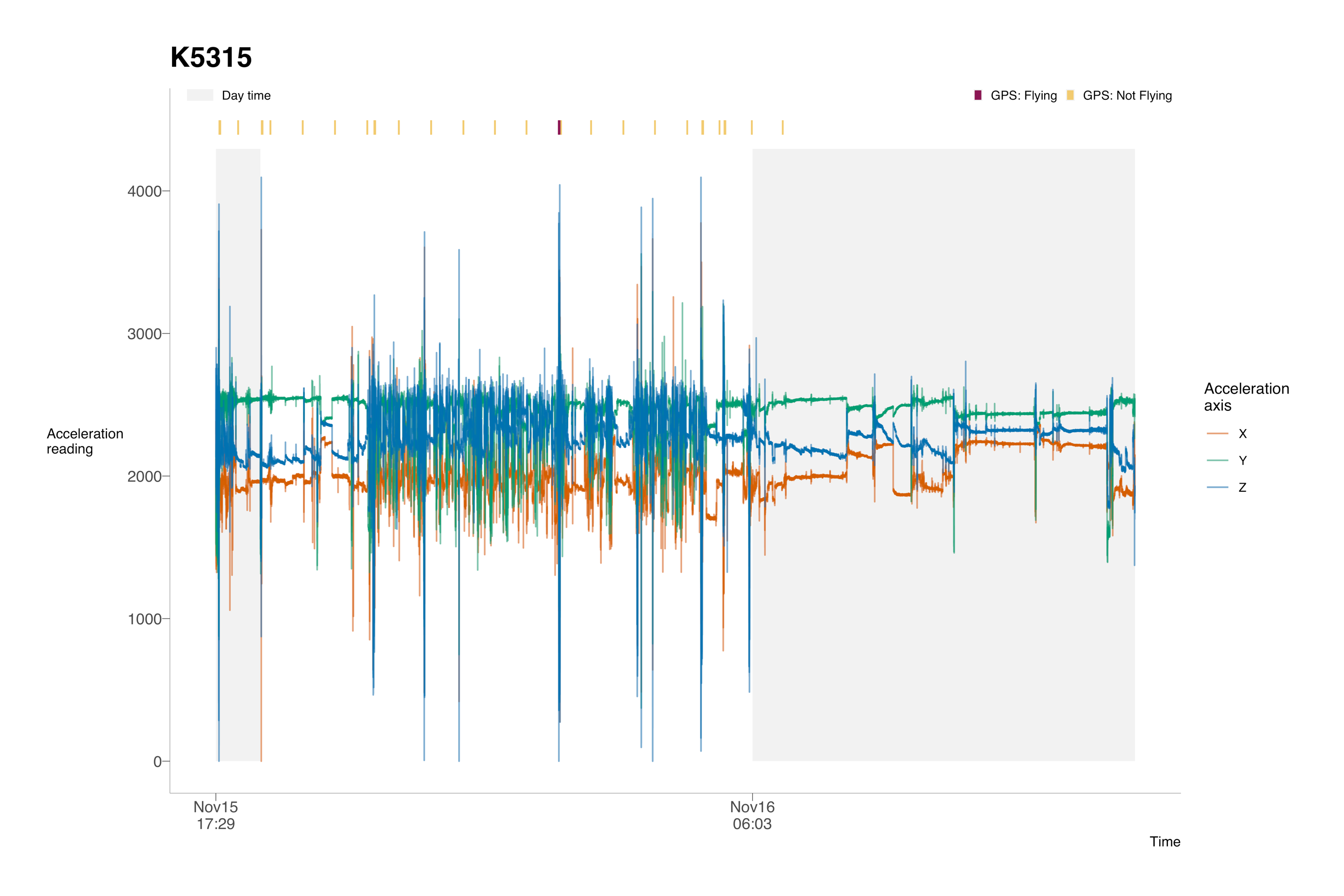

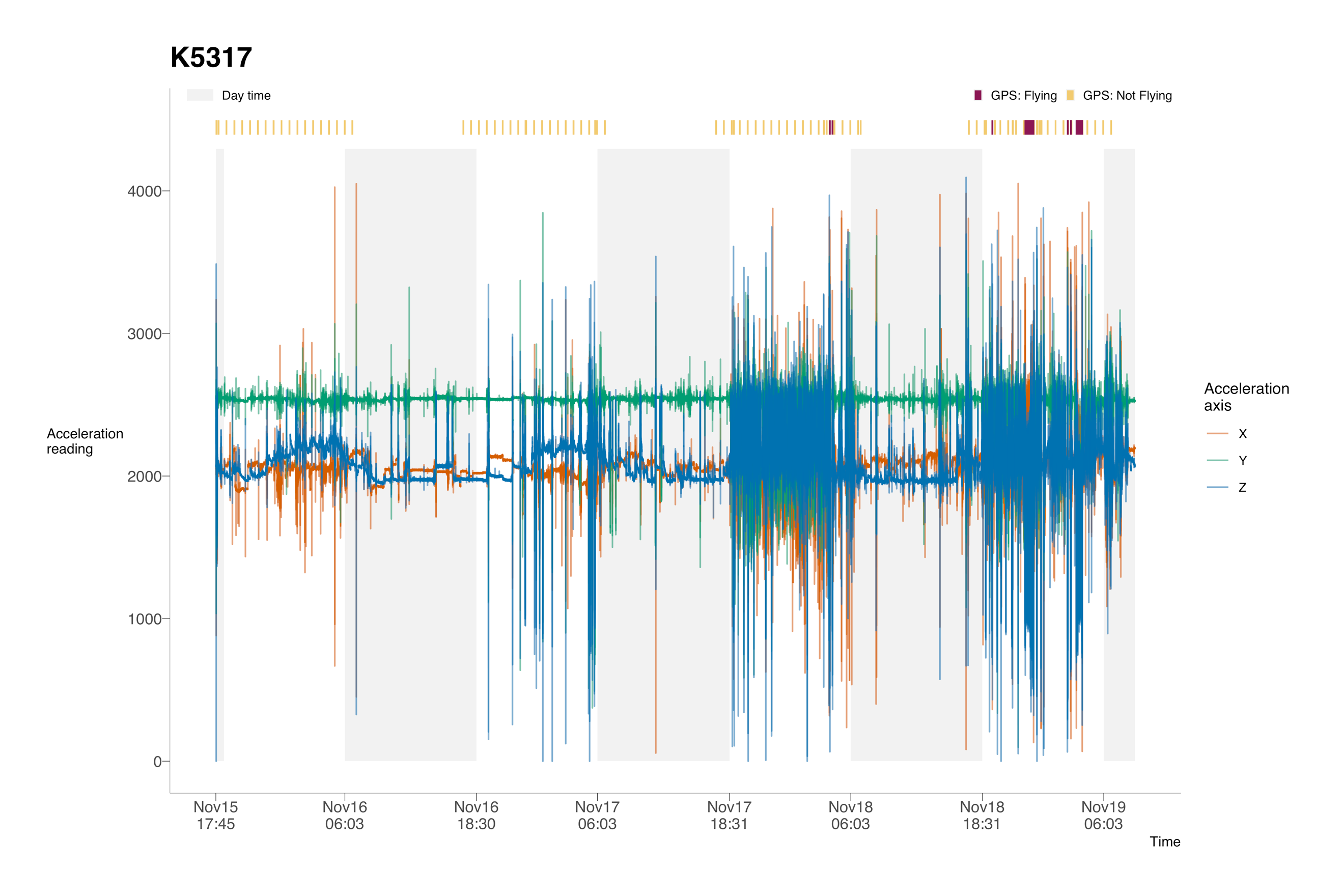

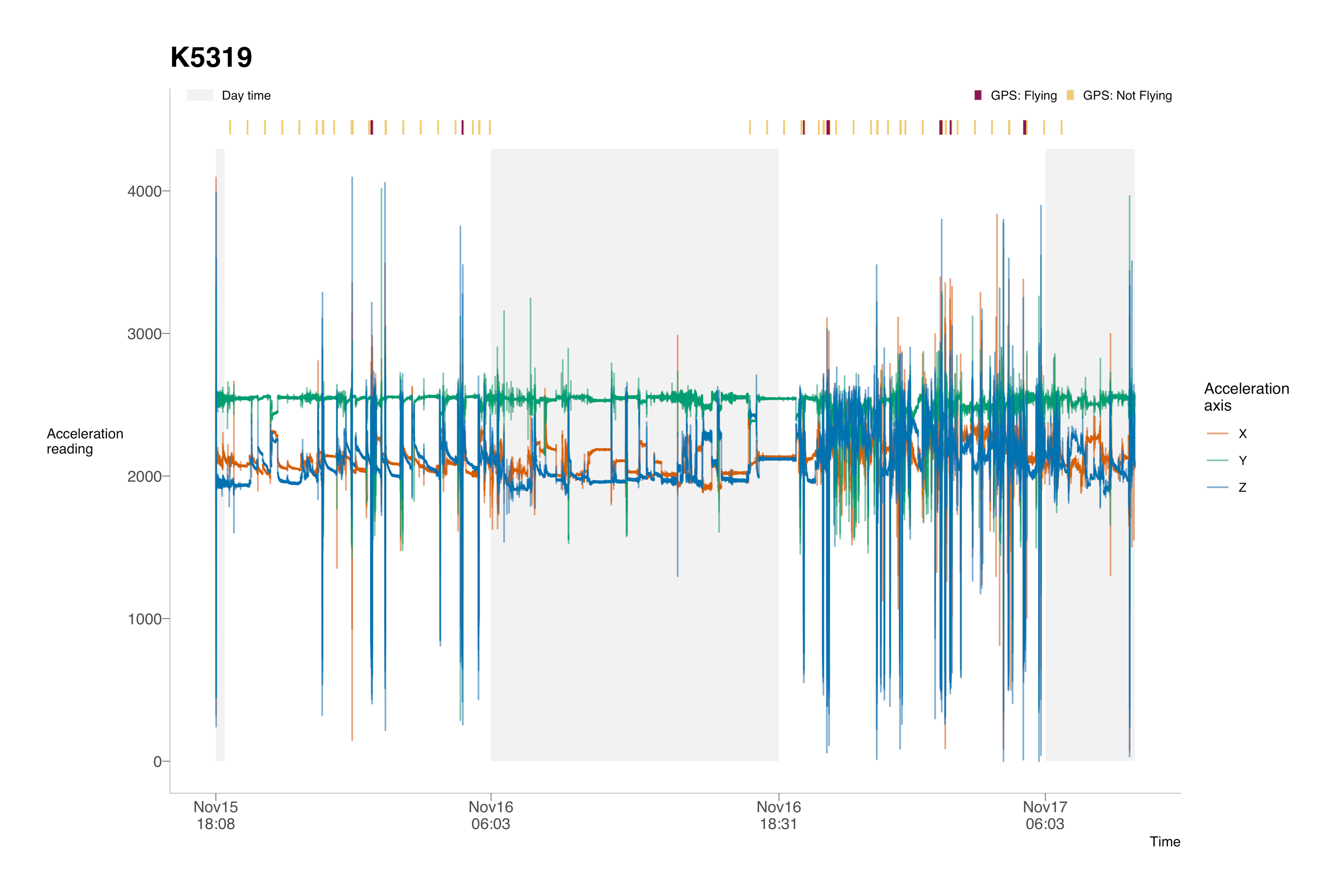
**
