# Supplementary figures and images for: Fruit bats in flight: a look into the movements of the ecologically important Eidolon helvum in Tanzania
Source: One Health Outlook. 2020 Aug 5;2:16. doi: 10.1186/s42522-020-00020-9 (PMC7402849; doi:10.1186/s42522-020-00020-9)

**Additional File 3**

**Figure S3. Map of GPS tracks of bats tagged with Argos tags in the Kilombero site.**


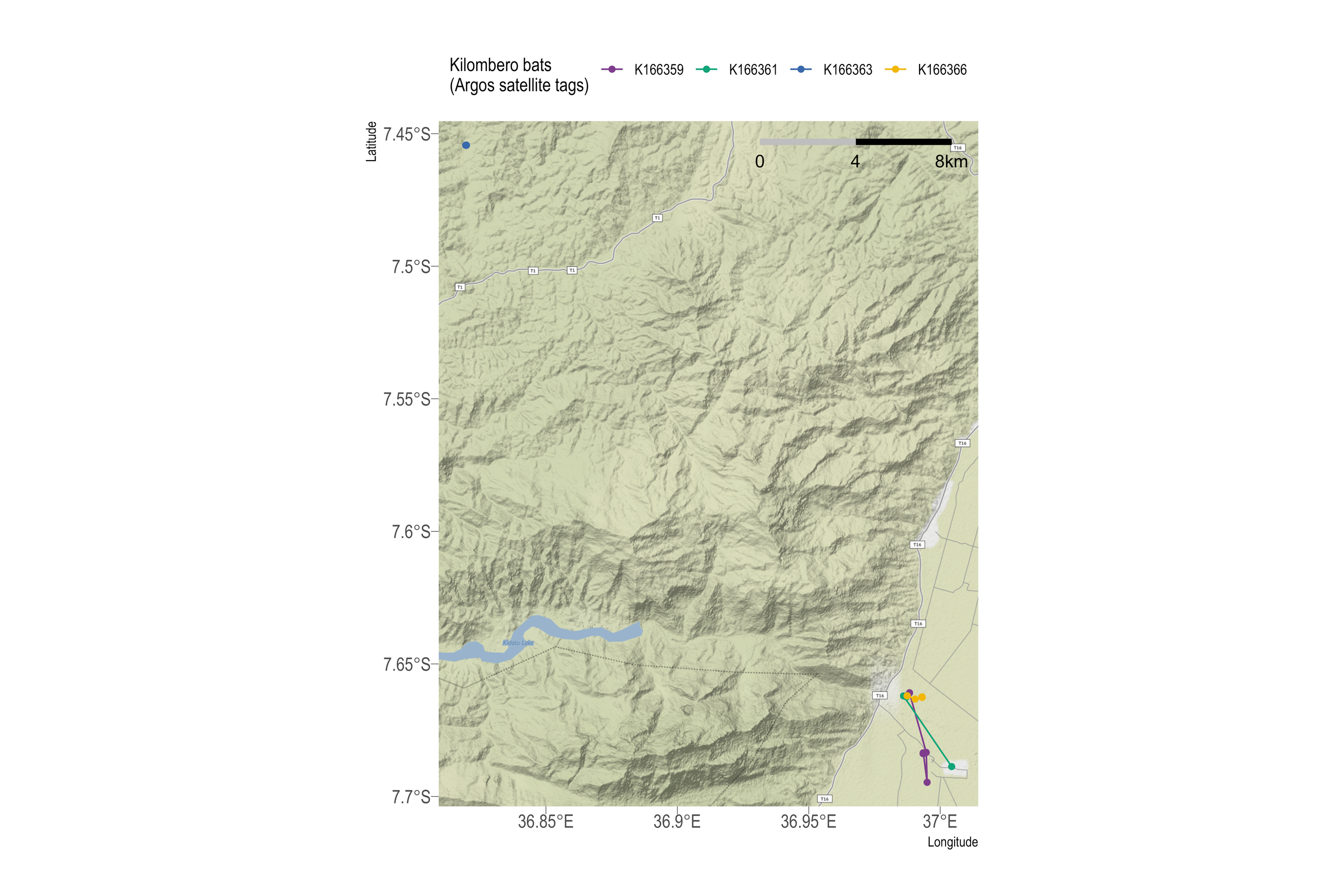

Supplement: Supplementary file 3 — Additional file 3 Figure S3. Map of GPS tracks of bats tagged with Argos tags in the Kilombero site. [file 42522_2020_20_MOESM3_ESM.docx]
