# Supplementary material for: Fruit bats in flight: a look into the movements of the ecologically important Eidolon helvum in Tanzania
Source: One Health Outlook. 2020 Aug 5;2:16. doi: 10.1186/s42522-020-00020-9 (PMC7402849; doi:10.1186/s42522-020-00020-9)

**Additional file 4**

**Cumulative distances flown by individual bats on each foraging night.**

**Table S4. Cumulative distances flown by individual bats on each foraging night.**

| Nights tracked | Bat | Nightly distance travelled (km) | | | | | |
| --- | --- | --- | --- | --- | --- | --- | --- |
|  |  | 1 | 2 | 3 | 4 | 5 | 6 |
| 5 | K5309 | 37.65 | 40.23 | 50.48 | 46.88 | 44.98 | - |
| 5 | K5310 | 2.07 | 5.39 | 96.46 | 97.57 | 77.66 | - |
| 2 | K5311 | 22.31 | 23.06 | - | - | - | - |
| 4 | K5312 | 0.73 | 7.89 | 28.82 | - | - | - |
| 6 | K5313 | 14.64 | 8.87 | 7.93 | 8.21 | 10.88 | 22.91 |
| 1 | K5315 | 2.92 | - | - | - | - | - |
| 4 | K5317 | 0.33 | 2.28 | 2.45 | 52.14 | - | - |
| 2 | K5319 | 5.58 | 10.6 | - | - | - | - |

**Figure S4. Line plot of cumulative distances flown by individual bats on each foraging night.**


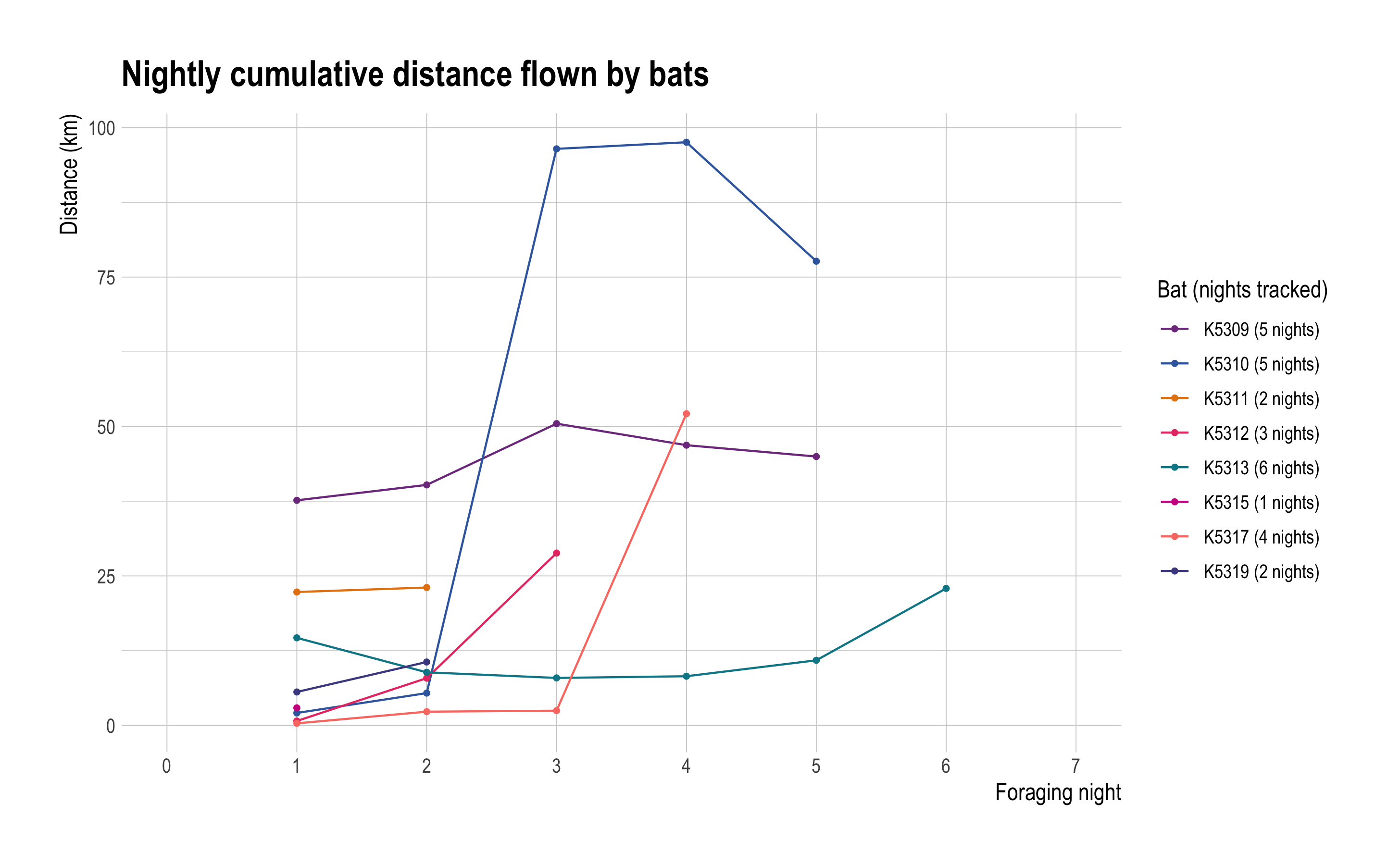

Supplement: Supplementary file 4 — Additional file 4 Table S4. Cumulative distances flown by individual bats on each foraging night. Figure S4. Line plot of cumulative distances flown by individual bats on each foraging night. [file 42522_2020_20_MOESM4_ESM.docx]
