# Supplementary material for: Fruit bats in flight: a look into the movements of the ecologically important Eidolon helvum in Tanzania
Source: One Health Outlook. 2020 Aug 5;2:16. doi: 10.1186/s42522-020-00020-9 (PMC7402849; doi:10.1186/s42522-020-00020-9)
Supplement: Supplementary file 5 — Additional file 5 Figure S5. Heatmap of mean hourly distances (km) flown by bats during individual foraging nights. Table S5.1. Mean cumulative hourly distances (km) flown by bats during individual foraging nights. Table S5.2. Cumulative hourly distances (km) flown by bats during foraging nights. [file 42522_2020_20_MOESM5_ESM.docx]

**Additional file 5**

**Cumulative hourly distances (km) flown by bats during individual foraging nights.**

**Figure S5: Heatmap of mean hourly distances (km) flown by bats during individual foraging nights**

**
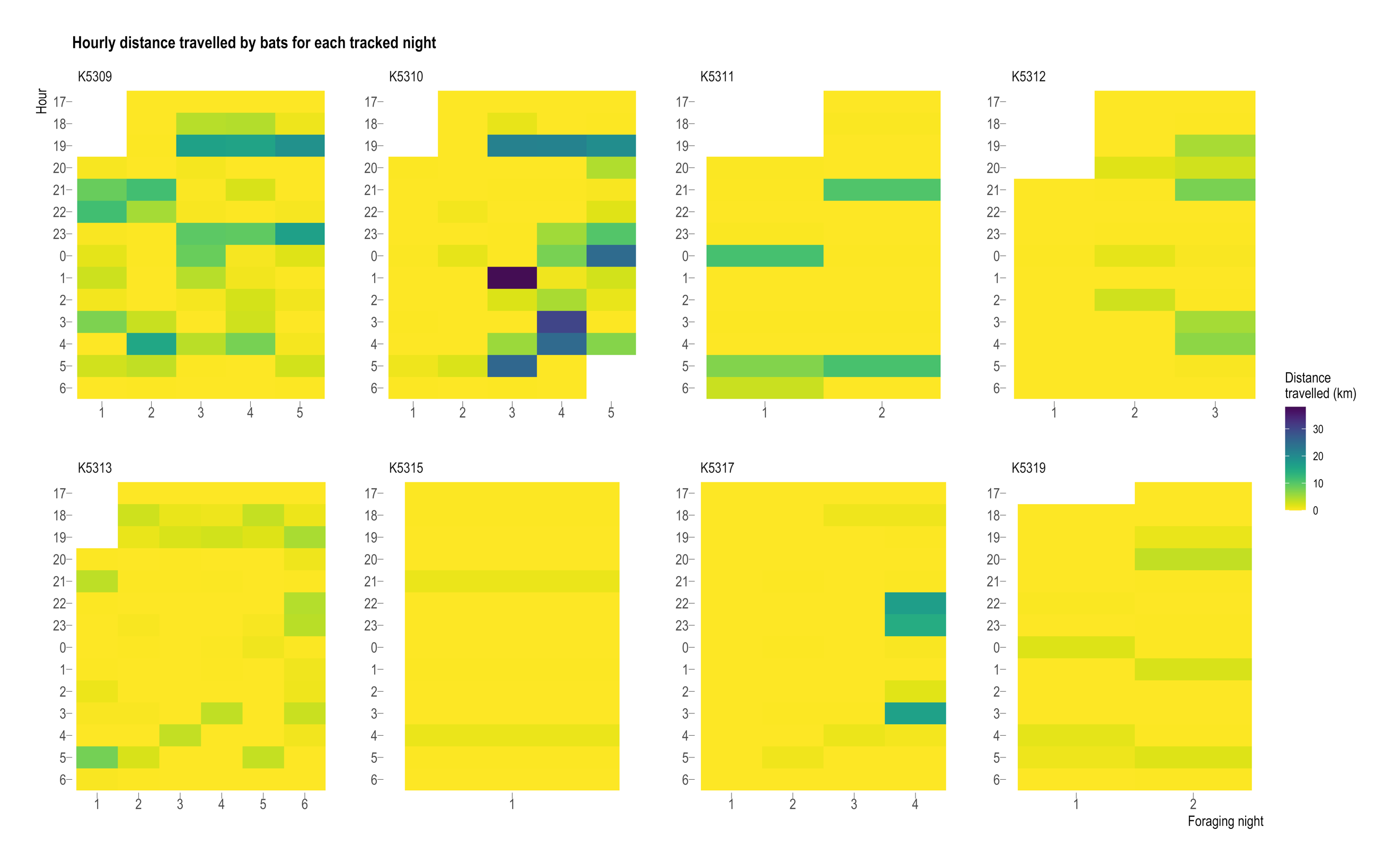
**

**Table S5.1 Mean cumulative hourly distances (km) flown by bats during individual foraging nights.**

|  | Cumulative hourly distance (km) | | |
| --- | --- | --- | --- |
| Bat | Mean | Min | Max |
| K5309 | 3.16 | 0 | 18.95 |
| K5310 | 4.12 | 0 | 38.08 |
| K5311 | 1.75 | 0 | 11.22 |
| K5312 | 0.89 | 0 | 7.68 |
| K5313 | 0.81 | 0 | 8.01 |
| K5315 | 0.19 | 0 | 1.15 |
| K5317 | 0.98 | 0 | 16.94 |
| K5319 | 0.51 | 0 | 3.47 |

**Table S5.2 Cumulative hourly distances (km) flown by bats during foraging nights.**

|  | Cumulative hourly distance (km) flown by each bat | | | | | | | |
| --- | --- | --- | --- | --- | --- | --- | --- | --- |
| Hour | K5309 | K5310 | K5311 | K5312 | K5313 | K5315 | K5317 | K5319 |
| 17 | 0  (0, 0.01) | 0  (0, 0.01) | 0  (0, 0) | 0.01  (0.01, 0.01) | 0  (0, 0.01) | 0.07  (0.07, 0.07) | 0.01  (0, 0.03) | 0.02  (0.02, 0.02) |
| 18 | 2.34  (0.03, 4.28) | 0.34  (0.01, 1.29) | 0.19  (0.19, 0.19) | 0.06  (0.02, 0.1) | 1.85  (0.96, 3.35) | 0.07  (0.07, 0.07) | 0.45  (0.02, 0.9) | 0.02  (0, 0.03) |
| 19 | 12.81  (0.05, 18.95) | 15.62  (0, 21.51) | 0.01  (0.01, 0.01) | 2.46  (0.01, 4.9) | 2.54  (1.21, 4.82) | 0  (0, 0) | 0.02  (0, 0.05) | 0.59  (0.01, 1.17) |
| 20 | 0.19  (0.01, 0.55) | 0.92  (0, 4.49) | 0.09  (0.01, 0.17) | 2.21  (1.74, 2.67) | 0.16  (0, 0.86) | 0  (0, 0) | 0  (0, 0.01) | 1.74  (0.01, 3.47) |
| 21 | 4.53  (0.03, 11.44) | 0.1  (0.01, 0.29) | 5.25  (0.04, 10.47) | 2.58  (0.03, 7.68) | 0.68  (0.02, 3.72) | 1.12  (1.12, 1.12) | 0.05  (0.02, 0.12) | 0.04  (0.02, 0.05) |
| 22 | 3.49  (0.06, 11.55) | 0.48  (0, 1.74) | 0  (0, 0.01) | 0  (0, 0.01) | 0.72  (0, 4.21) | 0.01  (0.01, 0.01) | 4.24  (0, 16.94) | 0.11  (0.02, 0.2) |
| 23 | 7.16  (0.05, 16.51) | 3.13  (0, 10.25) | 0.1  (0, 0.2) | 0.06  (0.01, 0.1) | 0.78  (0, 3.94) | 0  (0, 0) | 3.67  (0, 14.64) | 0.03  (0.01, 0.05) |
| 0 | 2.5  (0.08, 8.73) | 6.84  (0.06, 24.93) | 5.66  (0.09, 11.22) | 0.58  (0.09, 1.41) | 0.21  (0.03, 0.81) | 0.02  (0.02, 0.02) | 0.08  (0.01, 0.23) | 1  (0.06, 1.95) |
| 1 | 1.56  (0.01, 4.08) | 8.29  (0.01, 38.08) | 0  (0, 0.01) | 0.01  (0, 0.02) | 0.16  (0.01, 0.82) | 0.08  (0.08, 0.08) | 0.02  (0.01, 0.03) | 1.12  (0.03, 2.22) |
| 2 | 0.87  (0.01, 2.44) | 1.62  (0.01, 4.88) | 0.01  (0.01, 0.01) | 0.94  (0.01, 2.72) | 0.32  (0, 0.98) | 0  (0, 0) | 0.42  (0, 1.68) | 0  (0, 0.01) |
| 3 | 2.67  (0.02, 7.44) | 6.1  (0.03, 30.37) | 0.07  (0.06, 0.08) | 1.7  (0.01, 5.07) | 1.19  (0.02, 3.57) | 0.01  (0.01, 0.01) | 4.14  (0.01, 16.45) | 0.02  (0.02, 0.02) |
| 4 | 5.51  (0.03, 15.31) | 7.54  (0, 25.1) | 0  (0, 0) | 2.16  (0, 6.47) | 0.7  (0, 3.4) | 1.15  (1.15, 1.15) | 0.41  (0, 1.04) | 0.76  (0.04, 1.47) |
| 5 | 1.75  (0, 3.49) | 7.12  (0, 25.52) | 9.04  (7.1, 10.97) | 0.08  (0, 0.23) | 2.26  (0, 8.01) | 0.09  (0.09, 0.09) | 0.22  (0, 0.84) | 1.42  (0.96, 1.89) |
| 6 | 0.04  (0.02, 0.09) | 0.04  (0.01, 0.07) | 1.55  (0.03, 3.08) | 0.02  (0.01, 0.02) | 0.06  (0.01, 0.25) | 0.02  (0.02, 0.02) | 0.02  (0.01, 0.03) | 0.02  (0, 0.04) |
