# Supplementary material for: Fruit bats in flight: a look into the movements of the ecologically important Eidolon helvum in Tanzania
Source: One Health Outlook. 2020 Aug 5;2:16. doi: 10.1186/s42522-020-00020-9 (PMC7402849; doi:10.1186/s42522-020-00020-9)

**Additional file 6**

**Maps depicting 50% and 95% kernel density estimates of habitat utilization.**

**Figure S6: Contours of 50 (darker) and 95 (lighter) percent probability of utilization for each bat. The white star represents the tagging location/colony roost site.**

Bat with relatively large 95% KDE area (124413.78 ha)


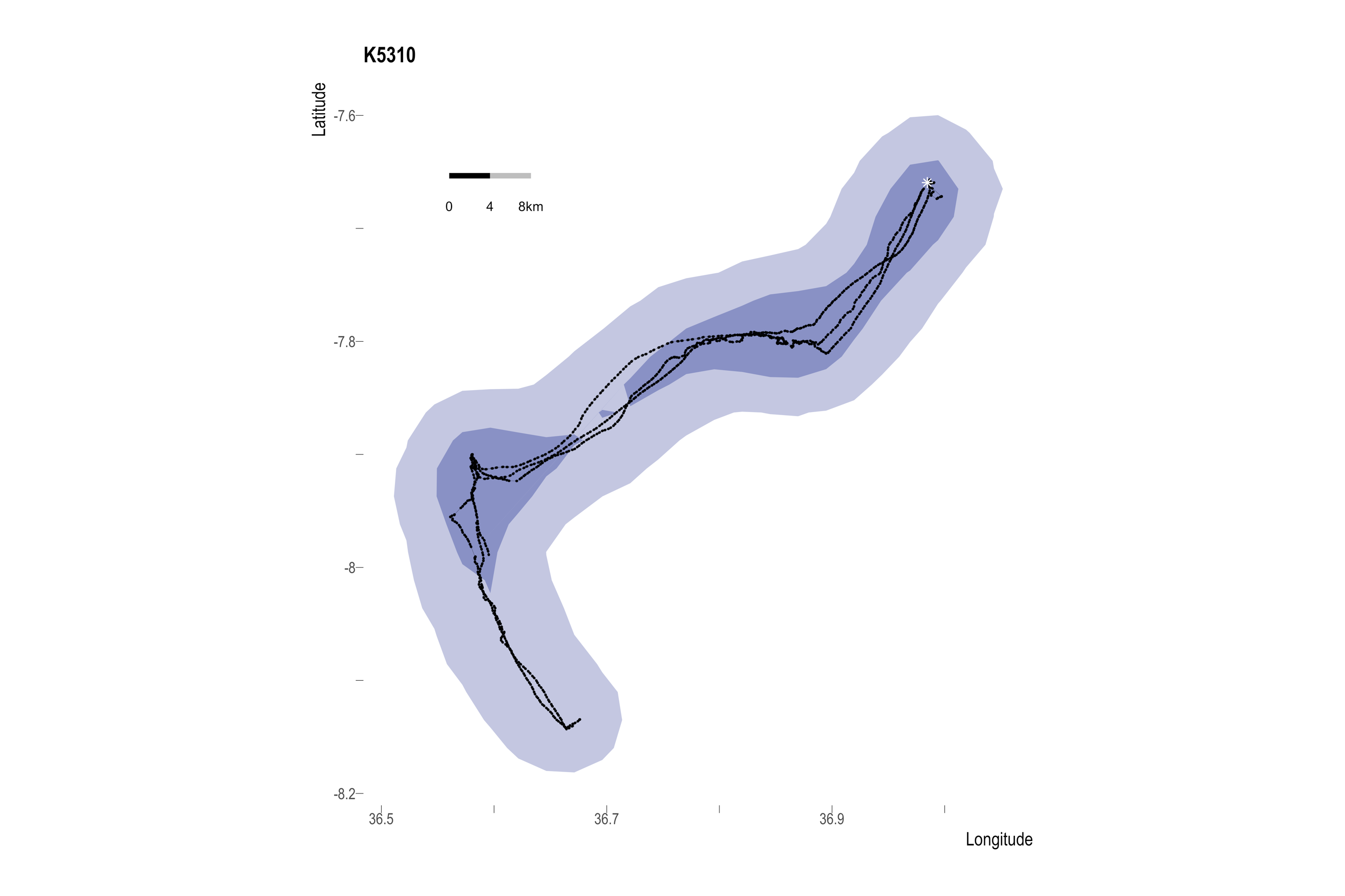


Bats with relatively medium 95% KDE areas (4861.94 to 18985.86 ha)
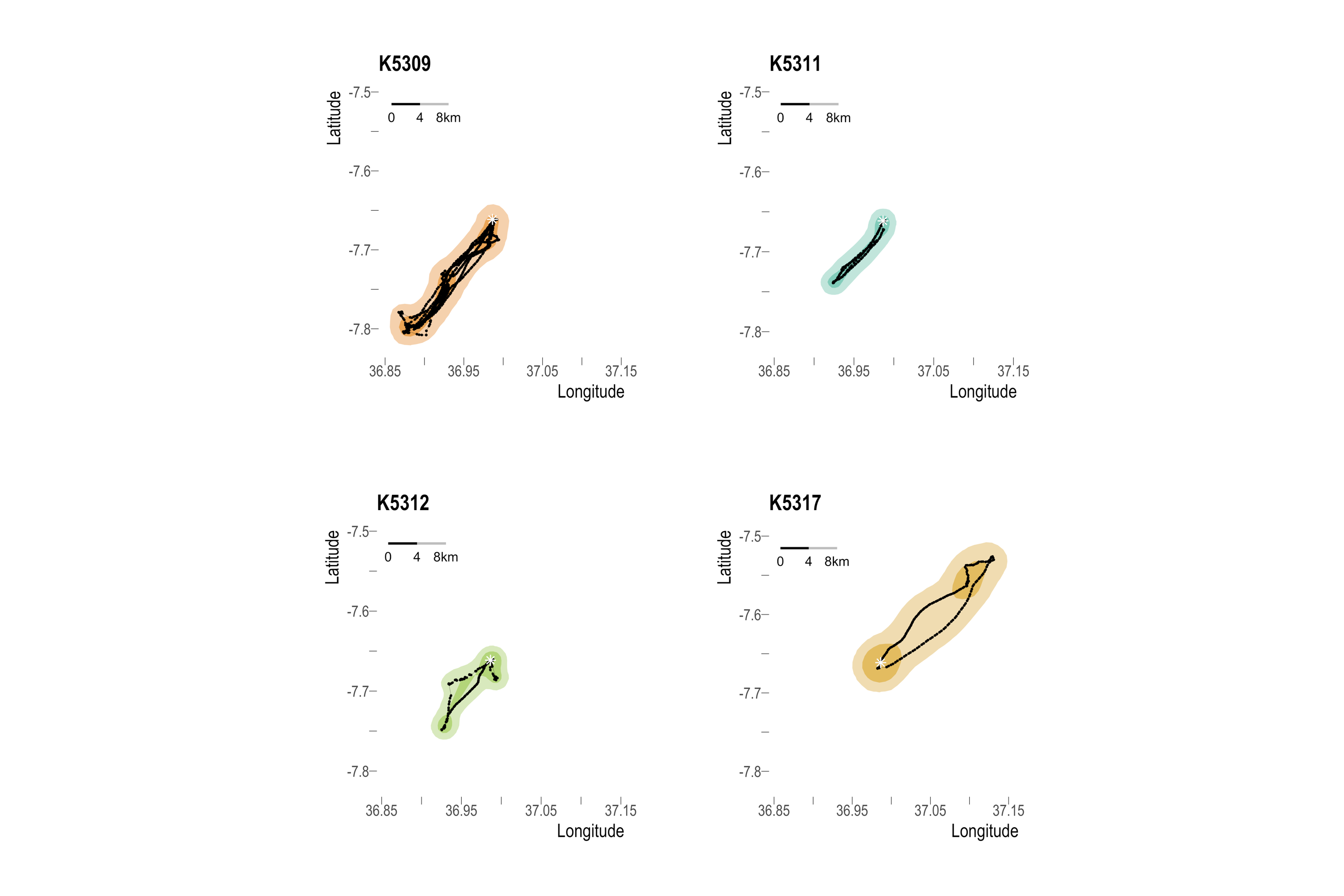


Bats with relatively small 95% KDE areas (96.88 to 1437.45 ha)
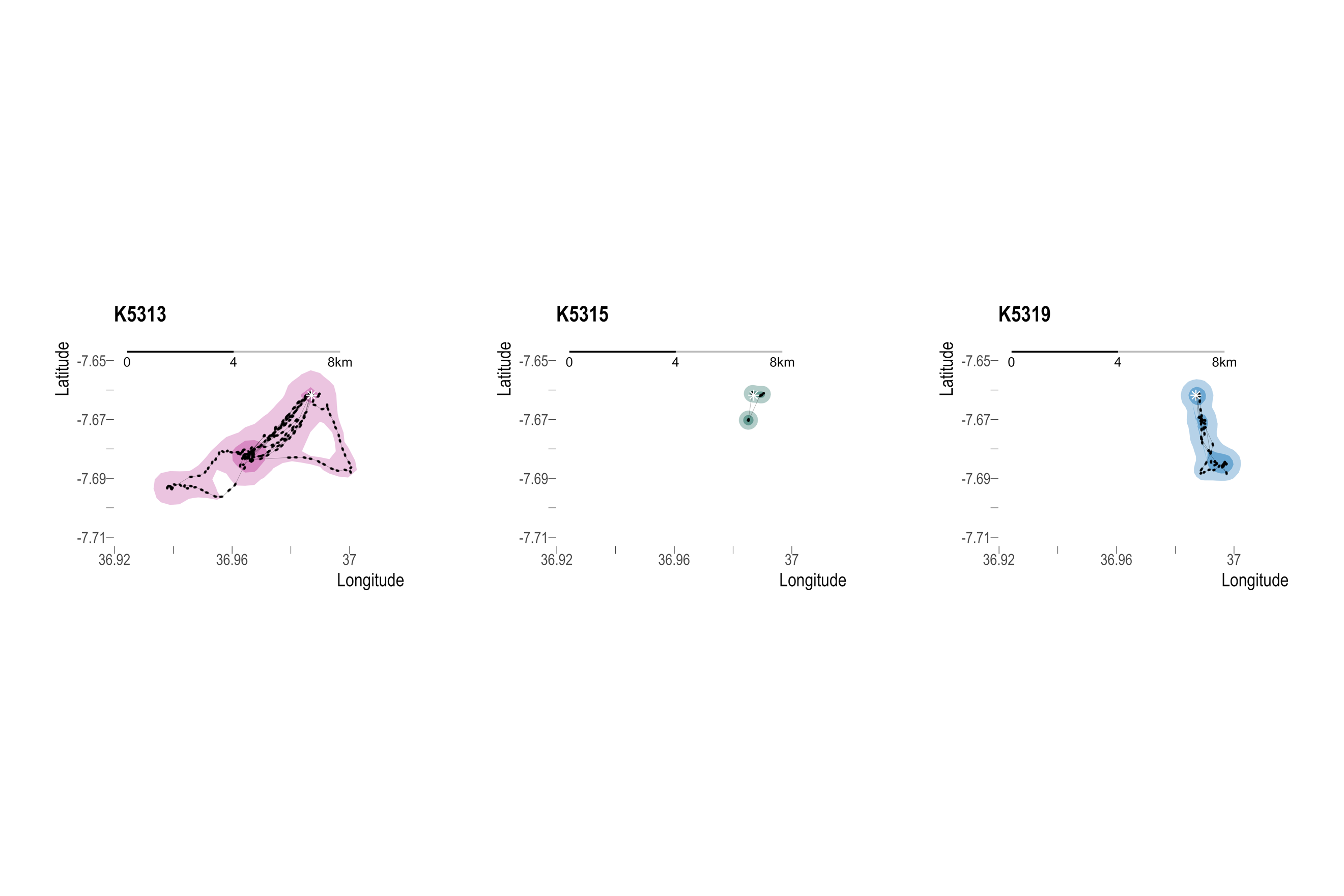

Supplement: Supplementary file 6 — Additional file 6 Figure S6. Contours of 50 (darker) and 95 (lighter) percent probability of utilization for each bat. The white star represents the tagging location/colony roost site. [file 42522_2020_20_MOESM6_ESM.docx]
