# Supplementary material for: Fruit bats in flight: a look into the movements of the ecologically important Eidolon helvum in Tanzania
Source: One Health Outlook. 2020 Aug 5;2:16. doi: 10.1186/s42522-020-00020-9 (PMC7402849; doi:10.1186/s42522-020-00020-9)

**Additional File 7**

**Figure S7: Maps of GPS tracks of bats tagged with e-obs loggers in Kilombero. Each map is followed by a map with a nightly breakdown of GPS tracks. The tracks are colored by individual bats, with the larger blue circle corresponding to colony location, the larger red circle corresponding to new day roosts (if any), and foraging/feeding roost areas depicted with yellow circles.**


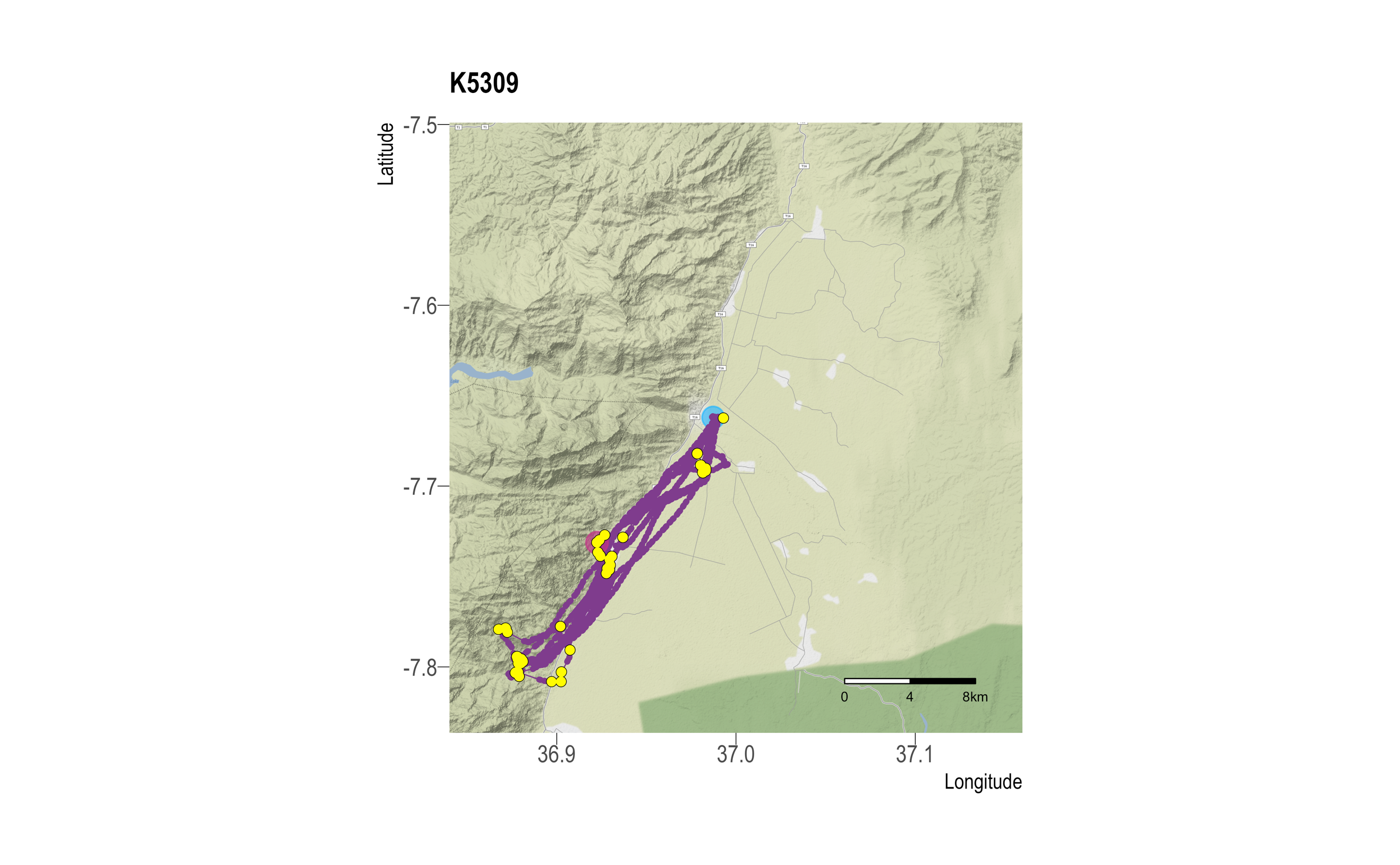

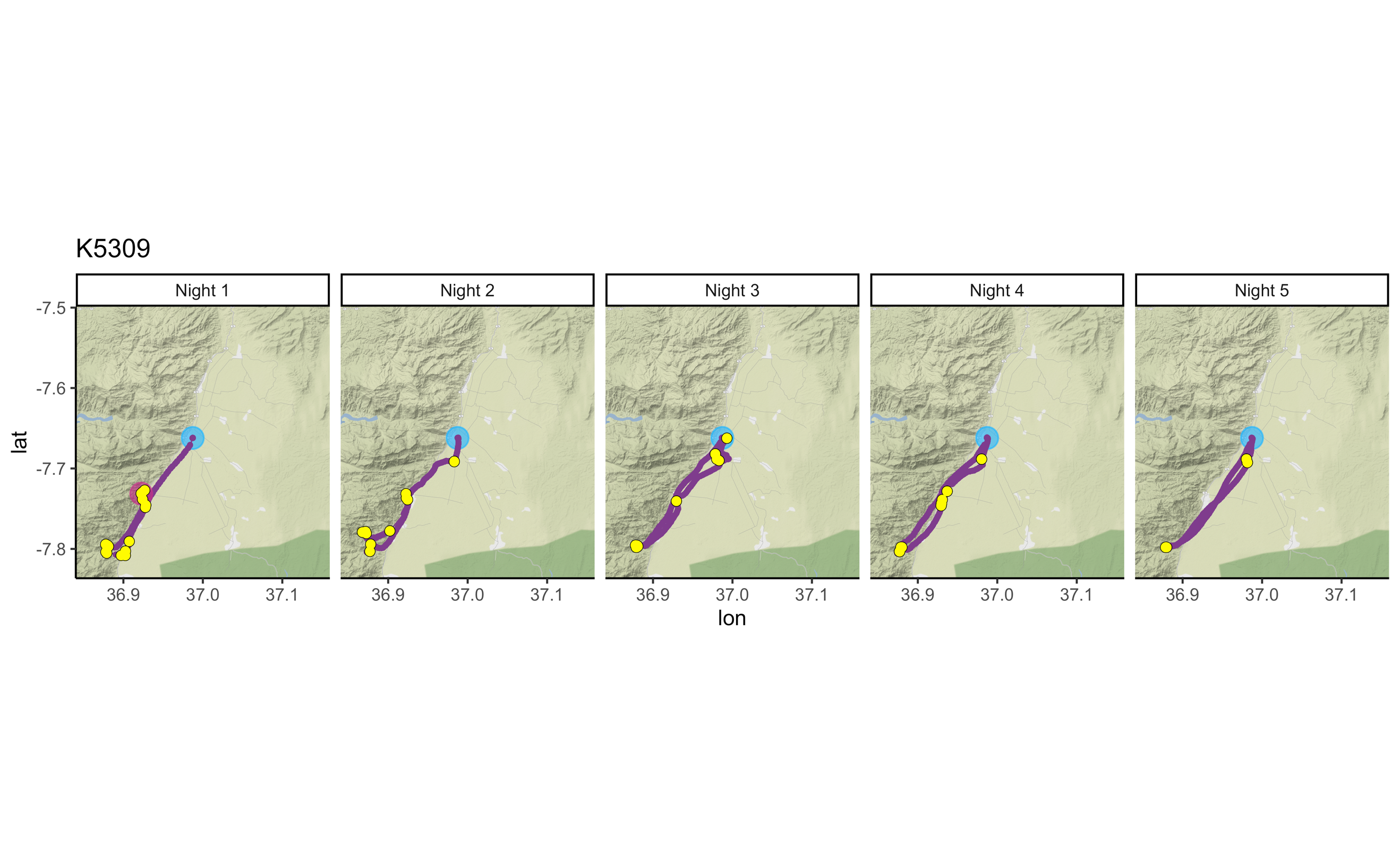

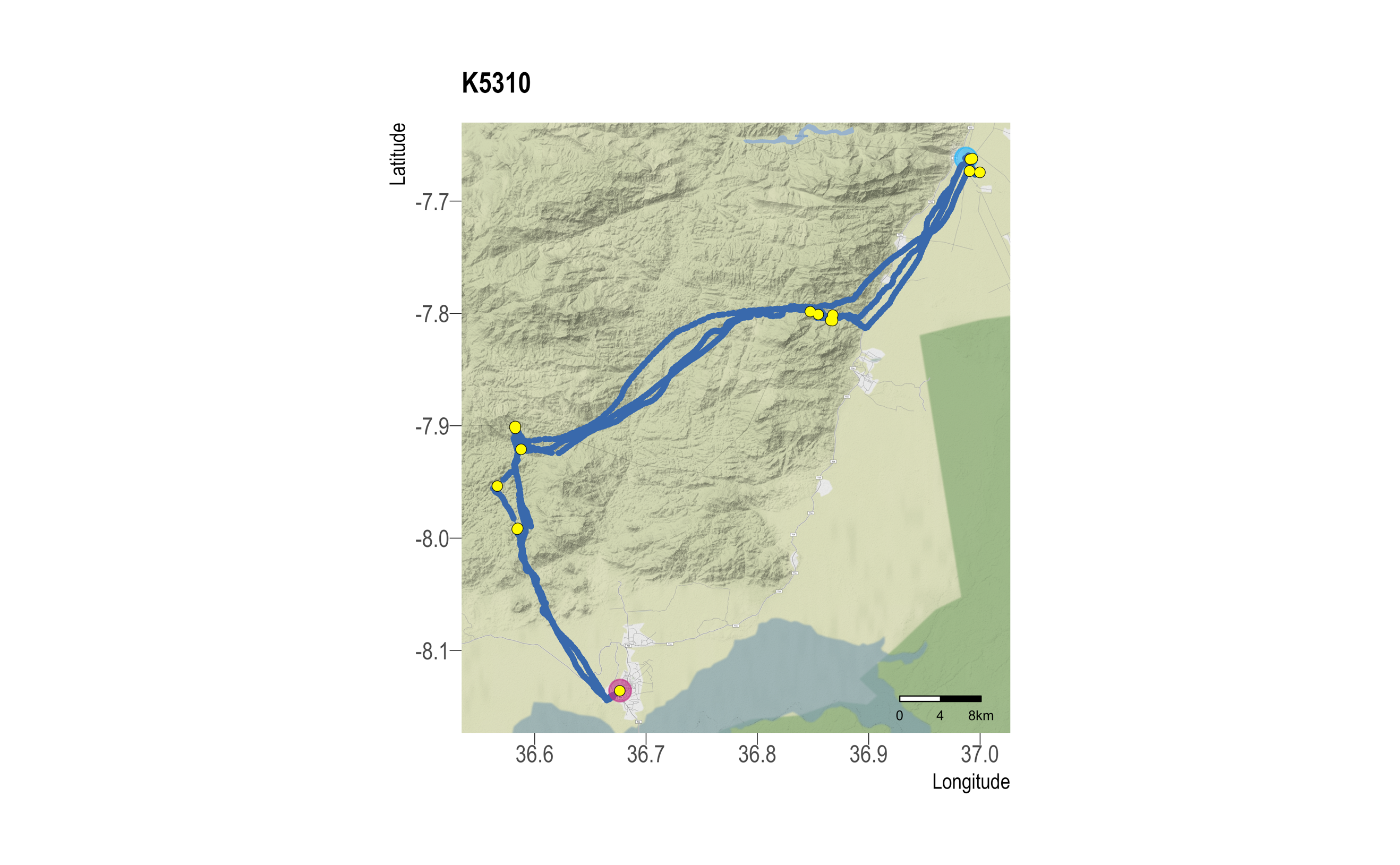

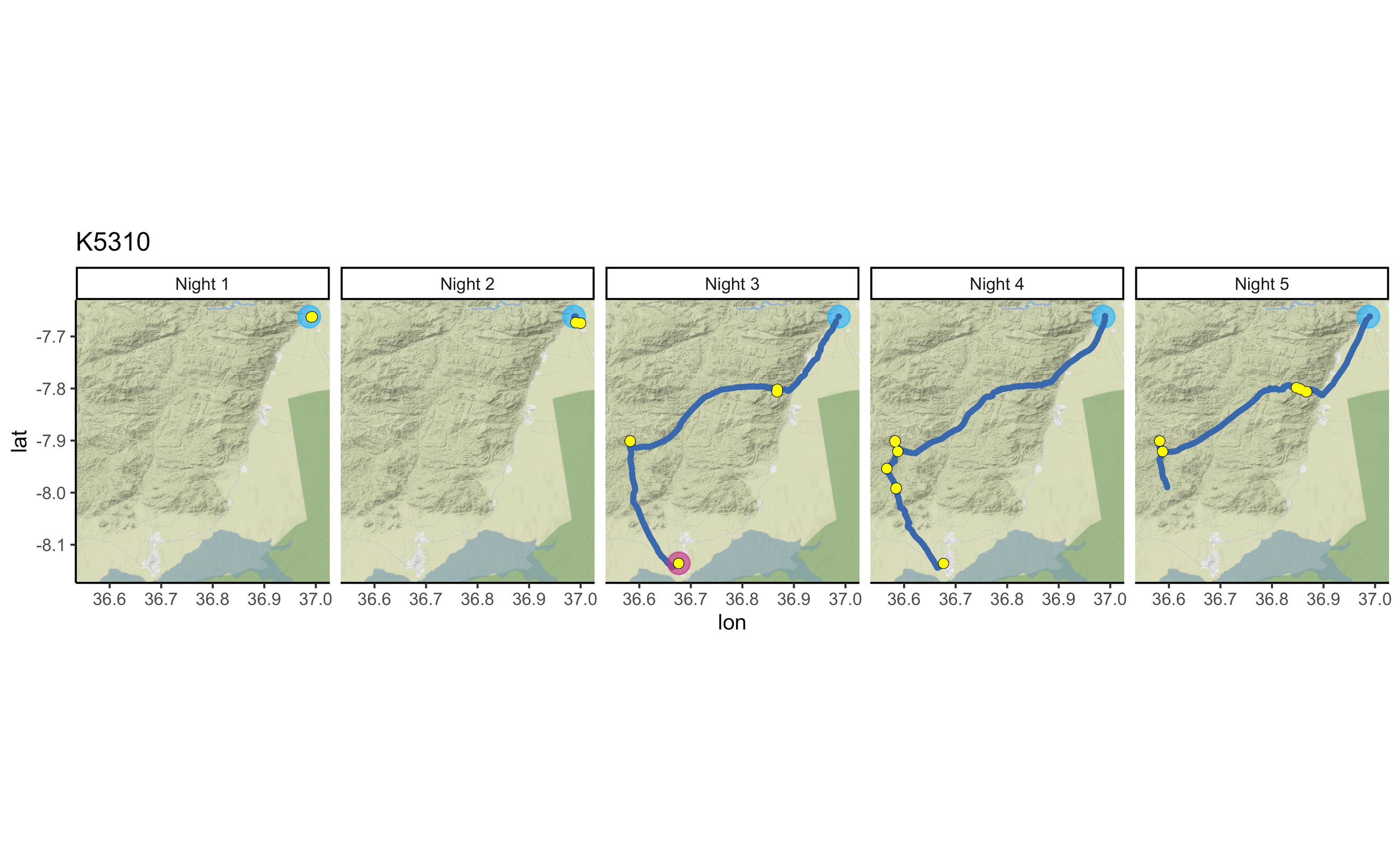

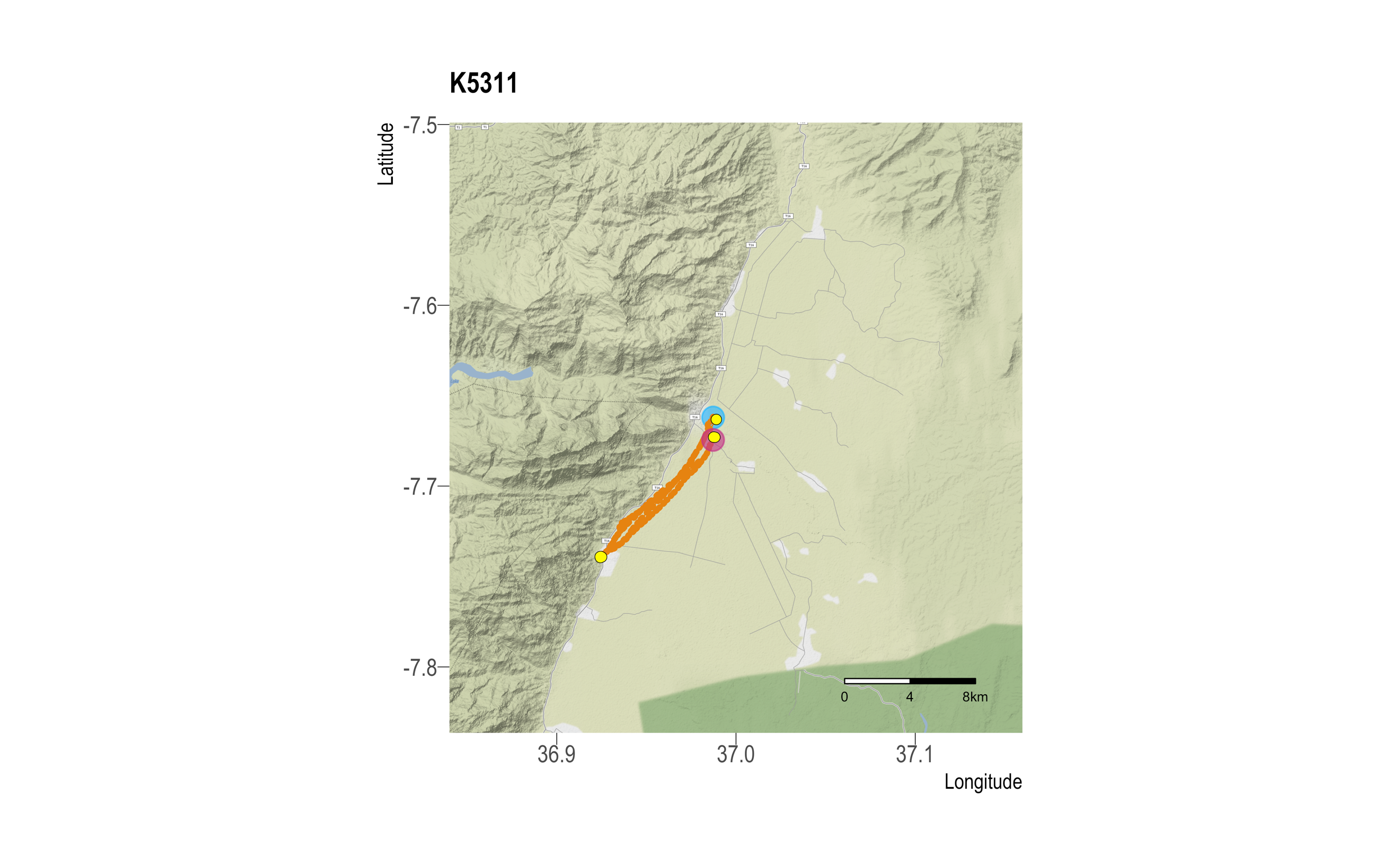

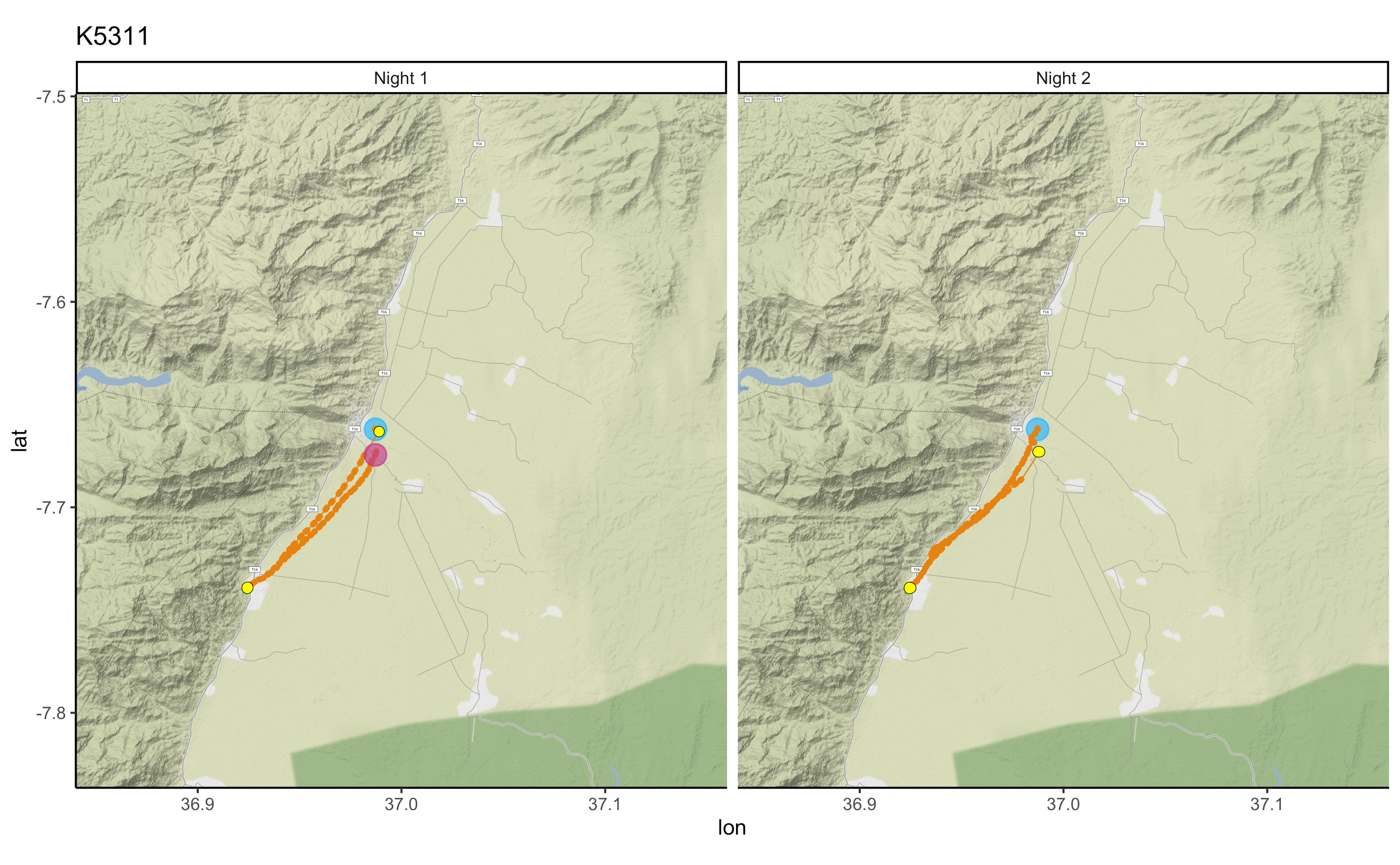


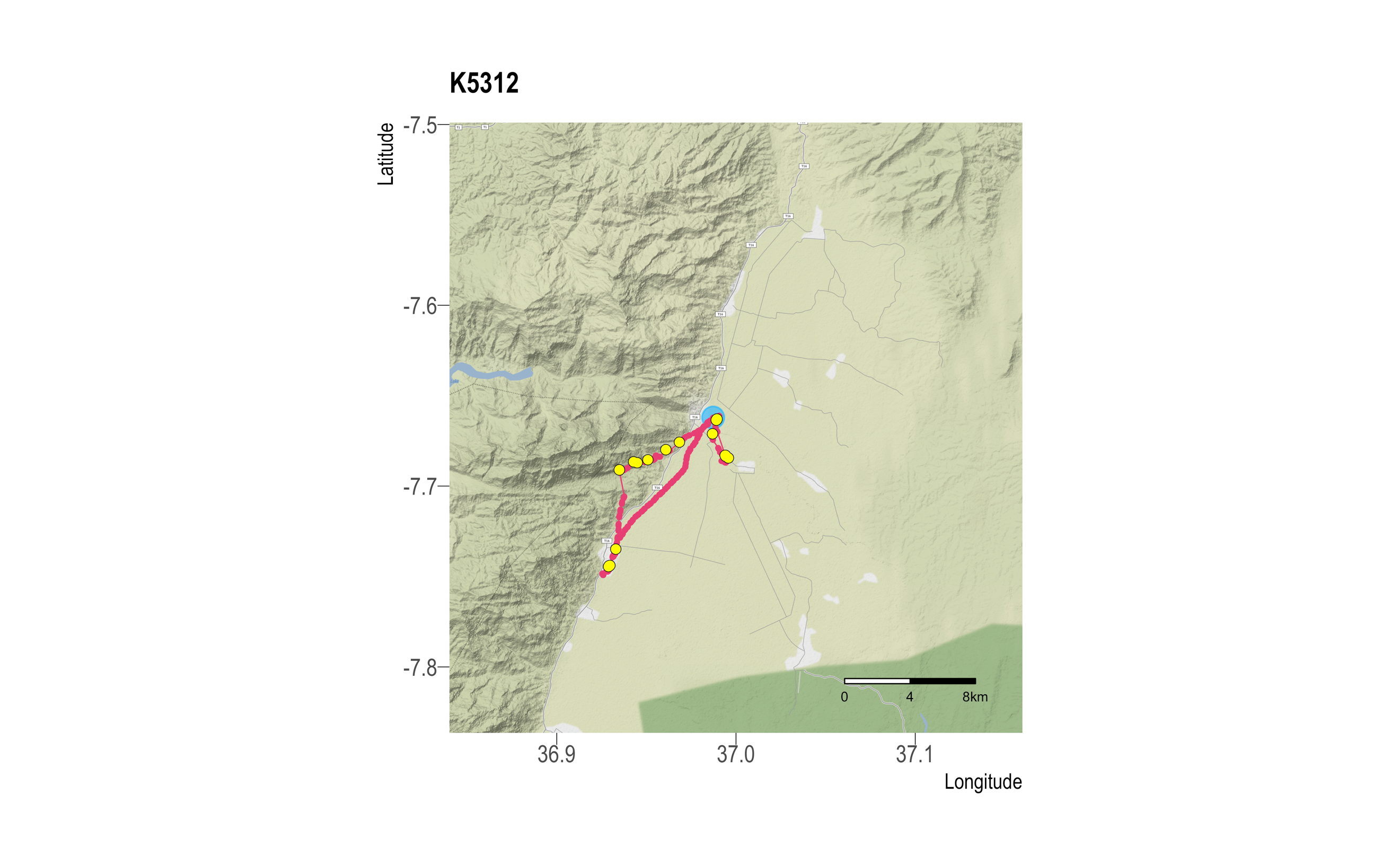

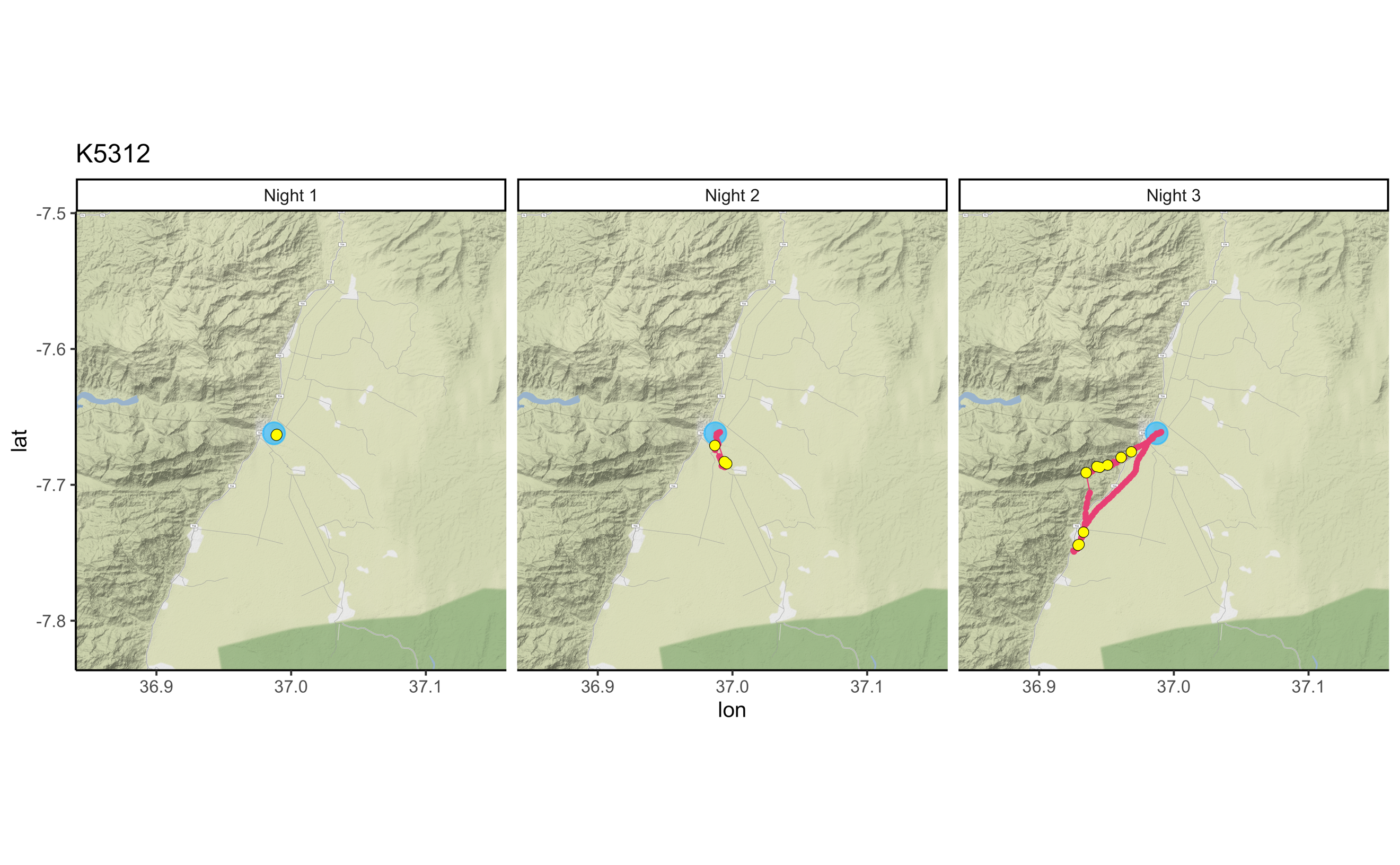

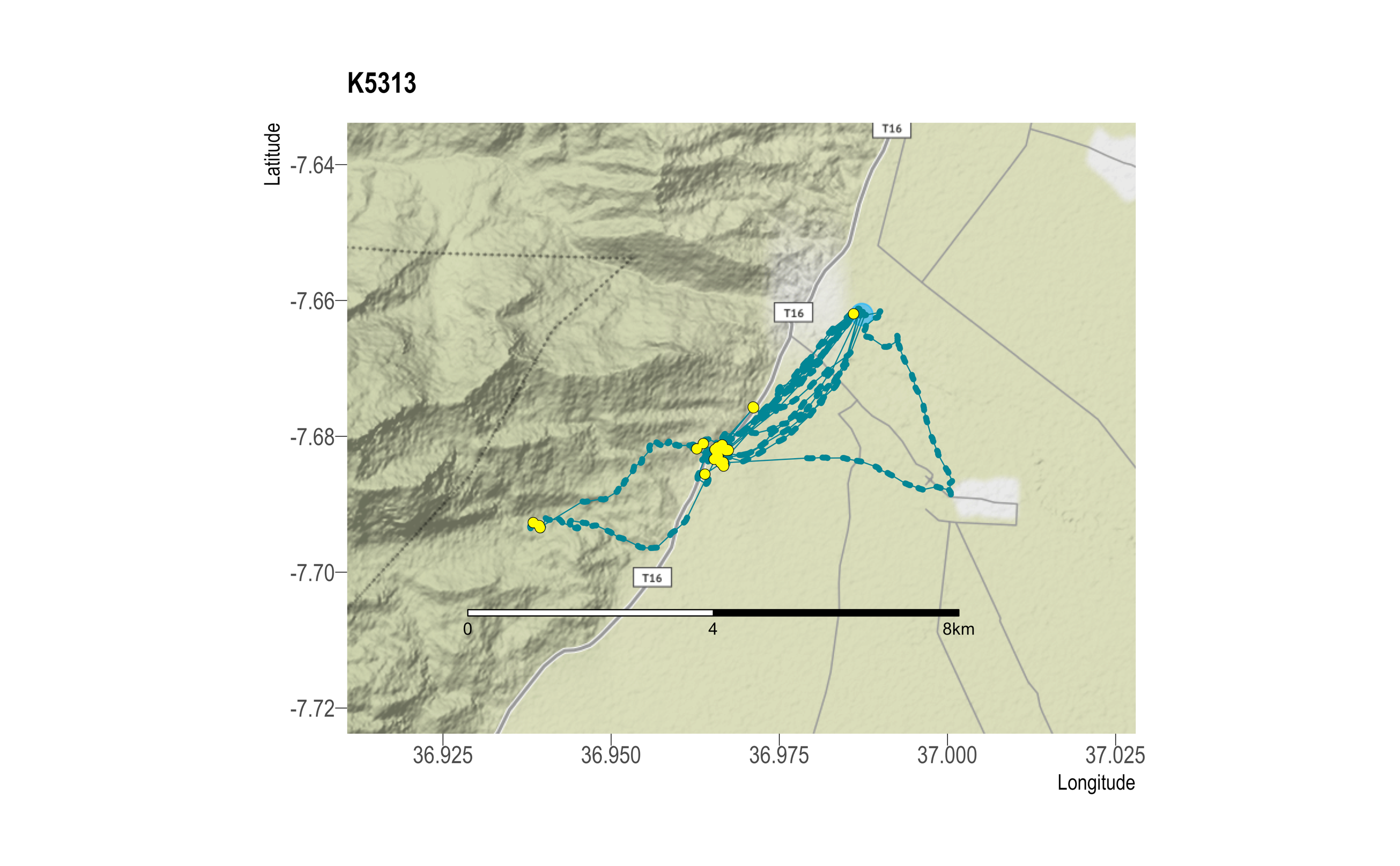

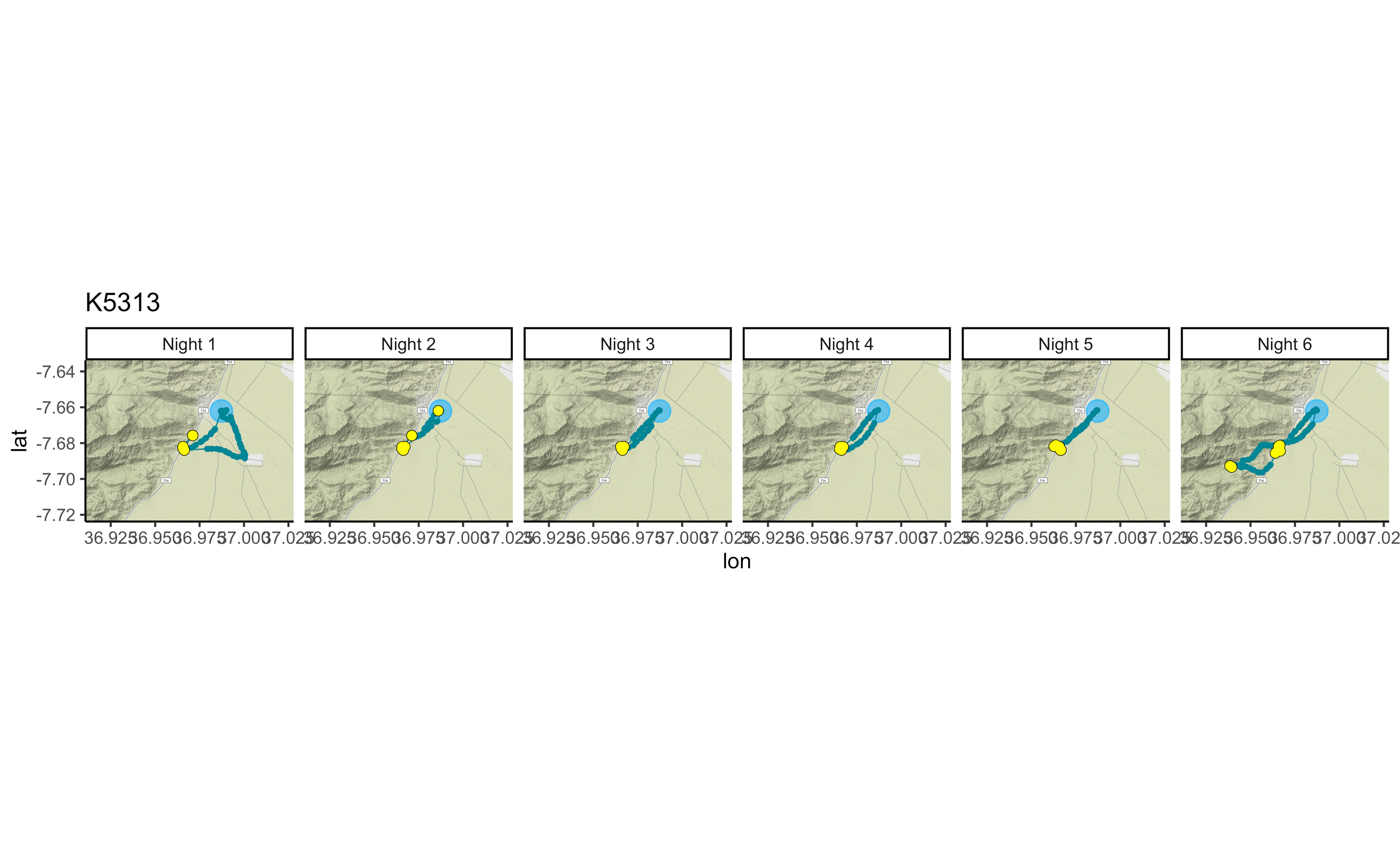

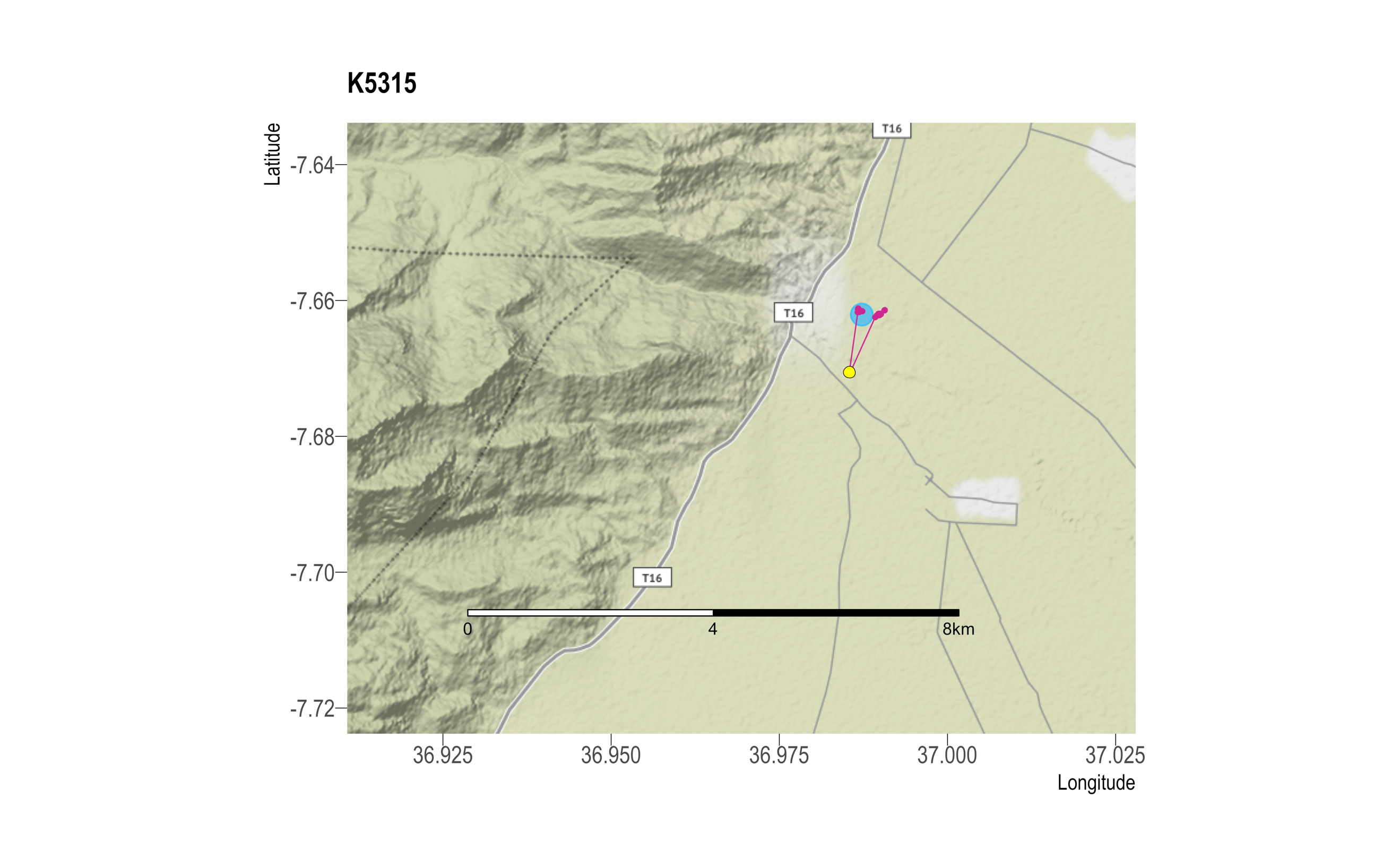

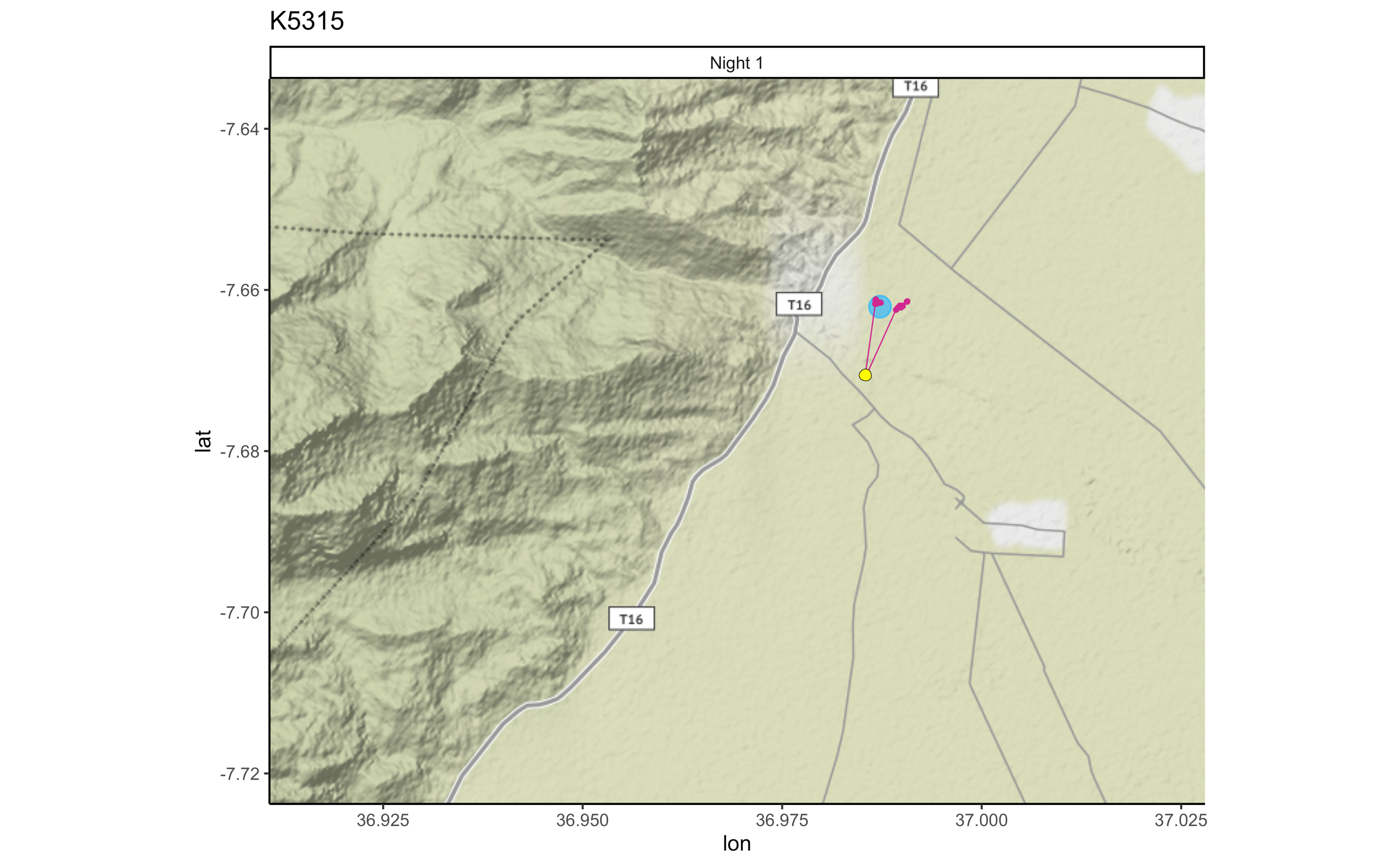

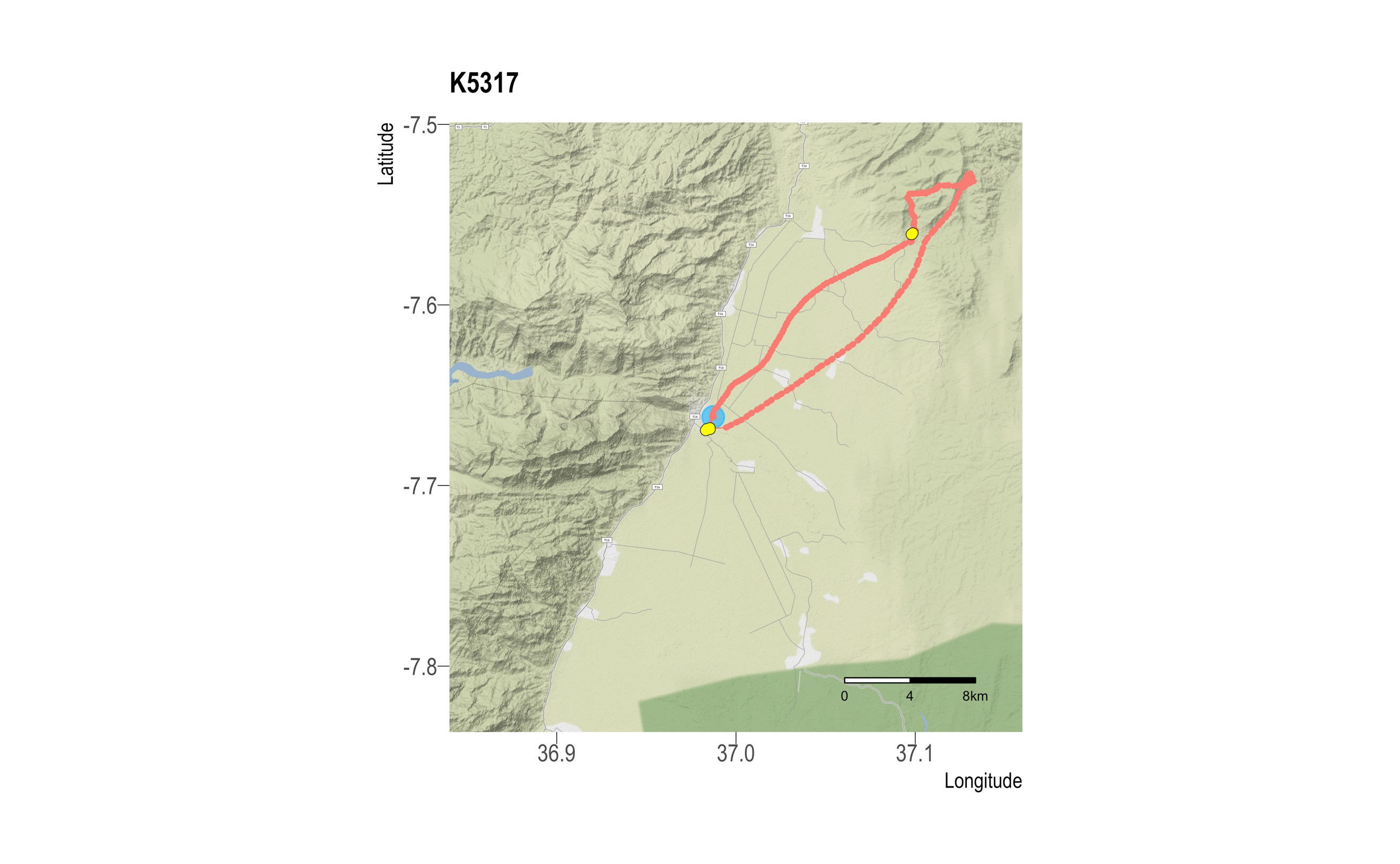

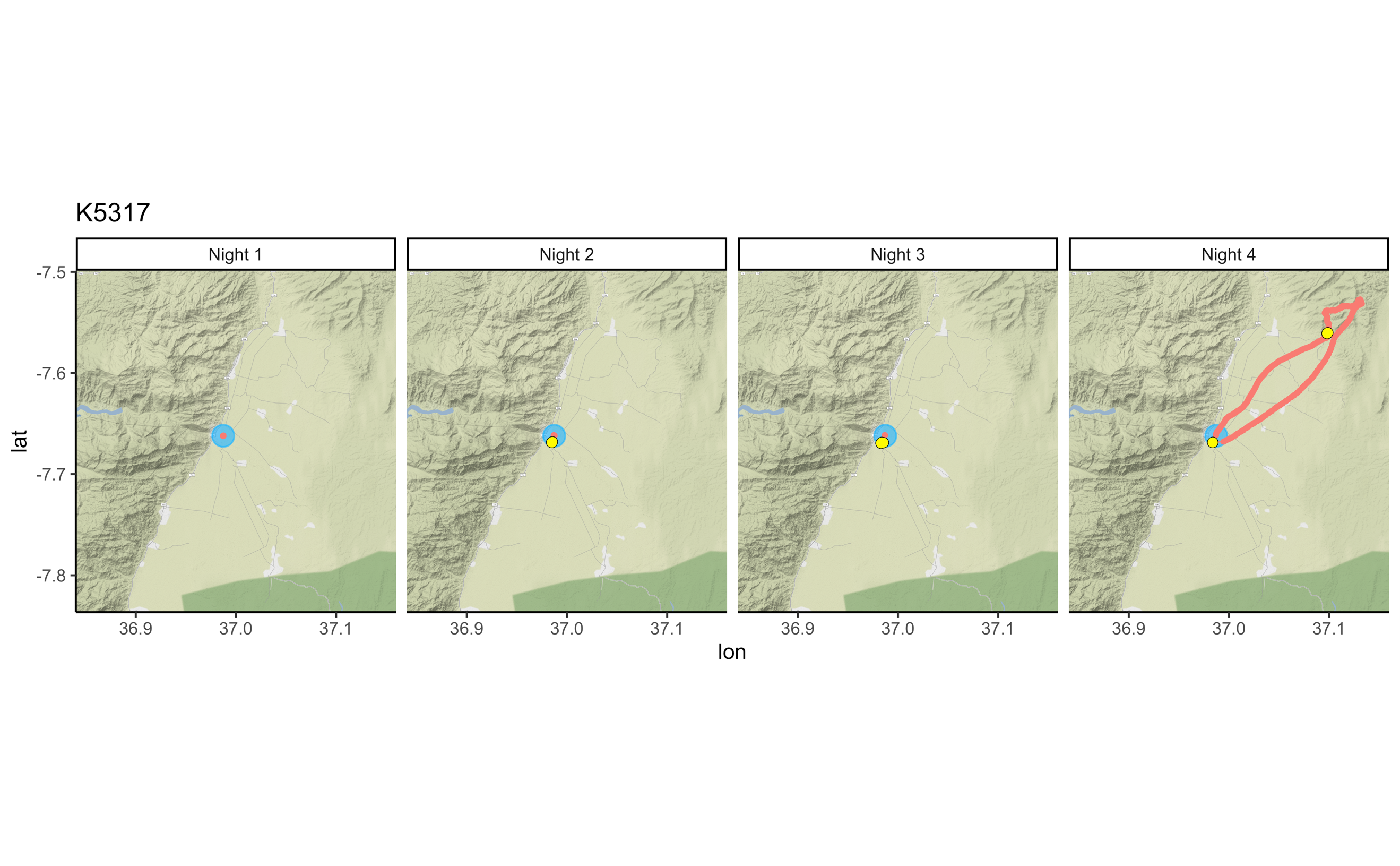

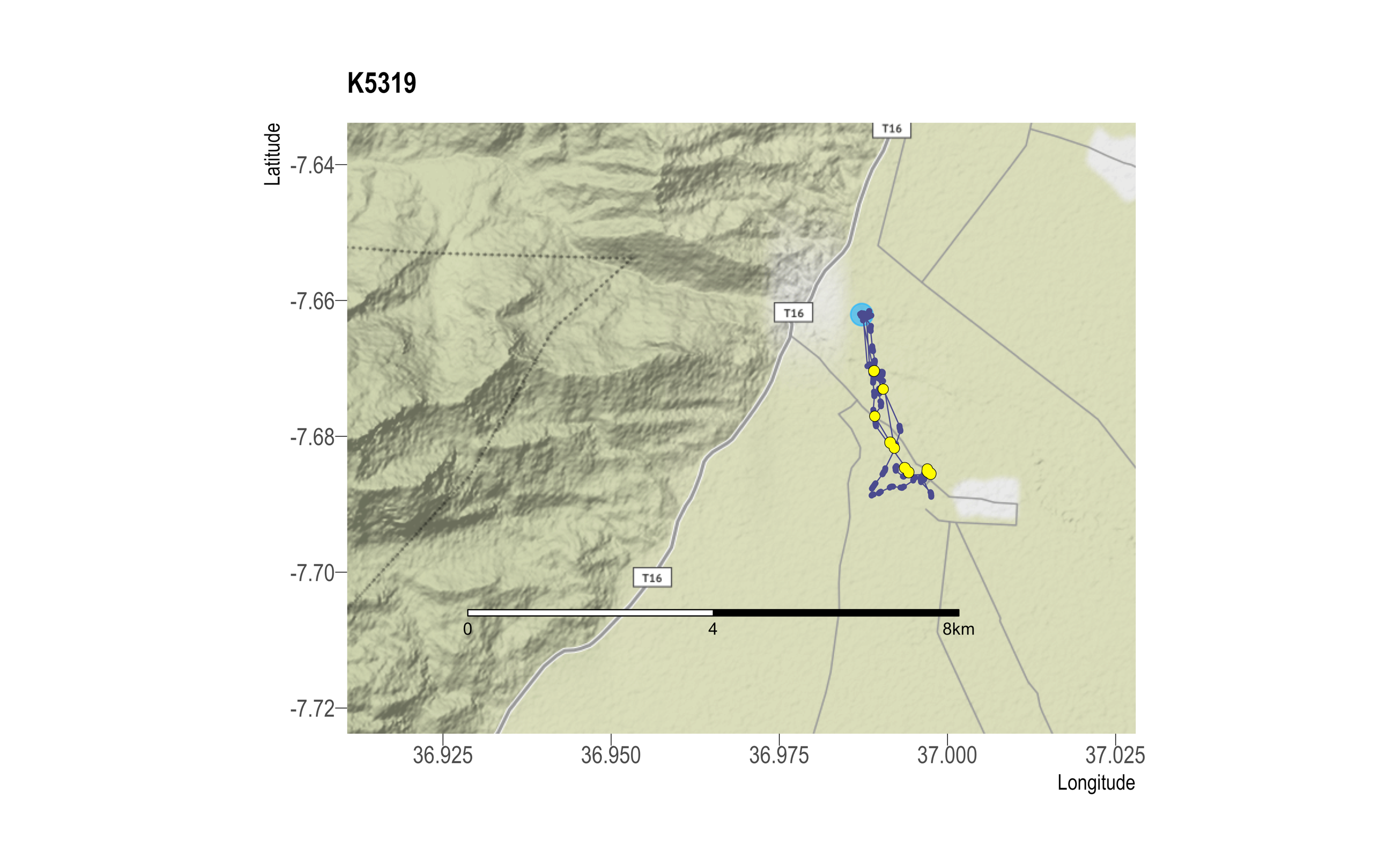

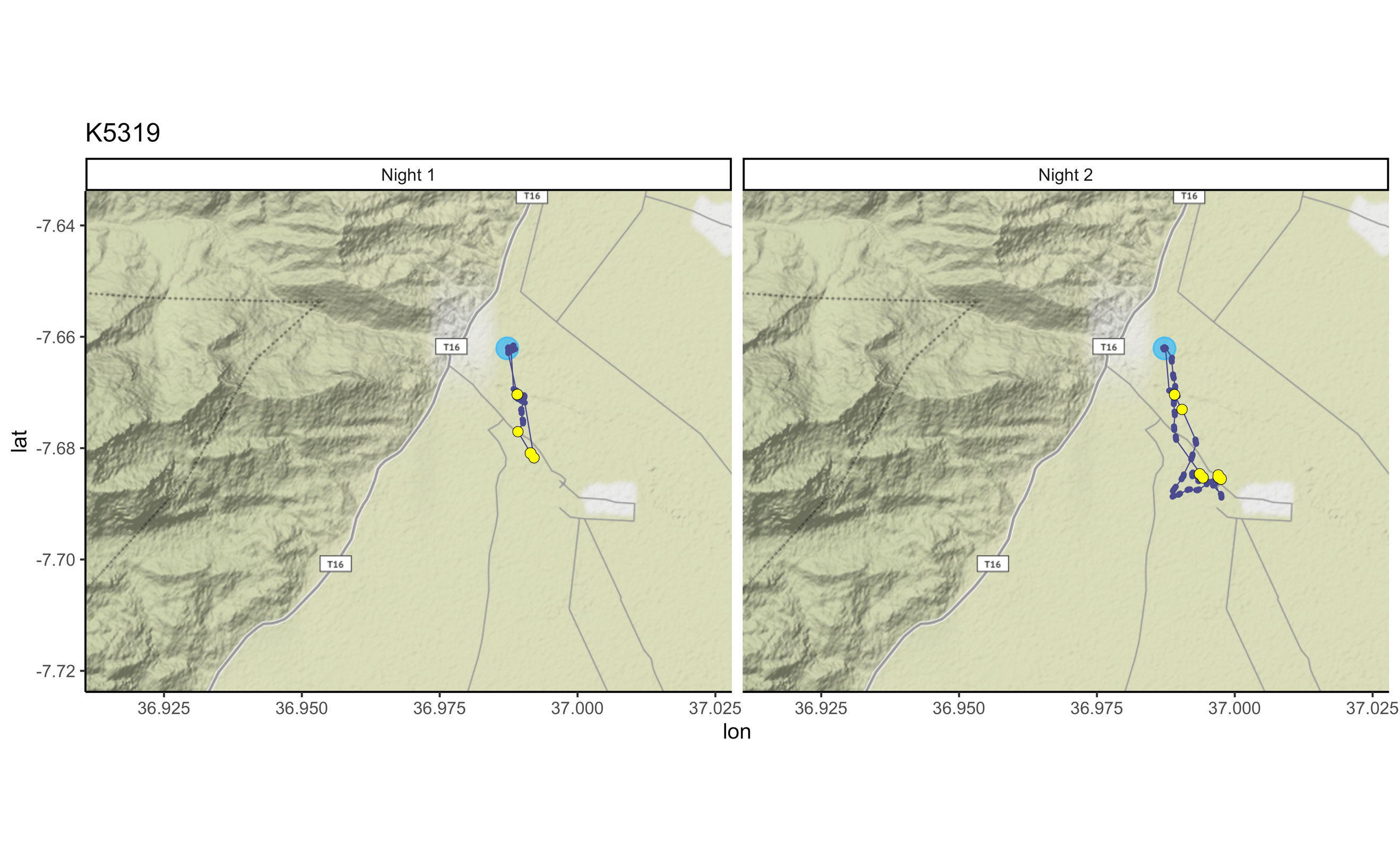

Supplement: Supplementary file 7 — Additional file 7 Figure S7. Maps of GPS tracks of bats tagged with e-obs loggers in Kilombero. Each map is followed by a map with a nightly breakdown of GPS tracks. The tracks are colored by individual bats, with the larger blue circle corresponding to colony location, the larger red circle corresponding to new day roosts (if any), and foraging/feeding roost areas depicted with yellow circles. [file 42522_2020_20_MOESM7_ESM.docx]
