# Supplementary material for: Fruit bats in flight: a look into the movements of the ecologically important Eidolon helvum in Tanzania
Source: One Health Outlook. 2020 Aug 5;2:16. doi: 10.1186/s42522-020-00020-9 (PMC7402849; doi:10.1186/s42522-020-00020-9)

**Additional File 8**

**Pictures of camera traps and collected images.**

­­
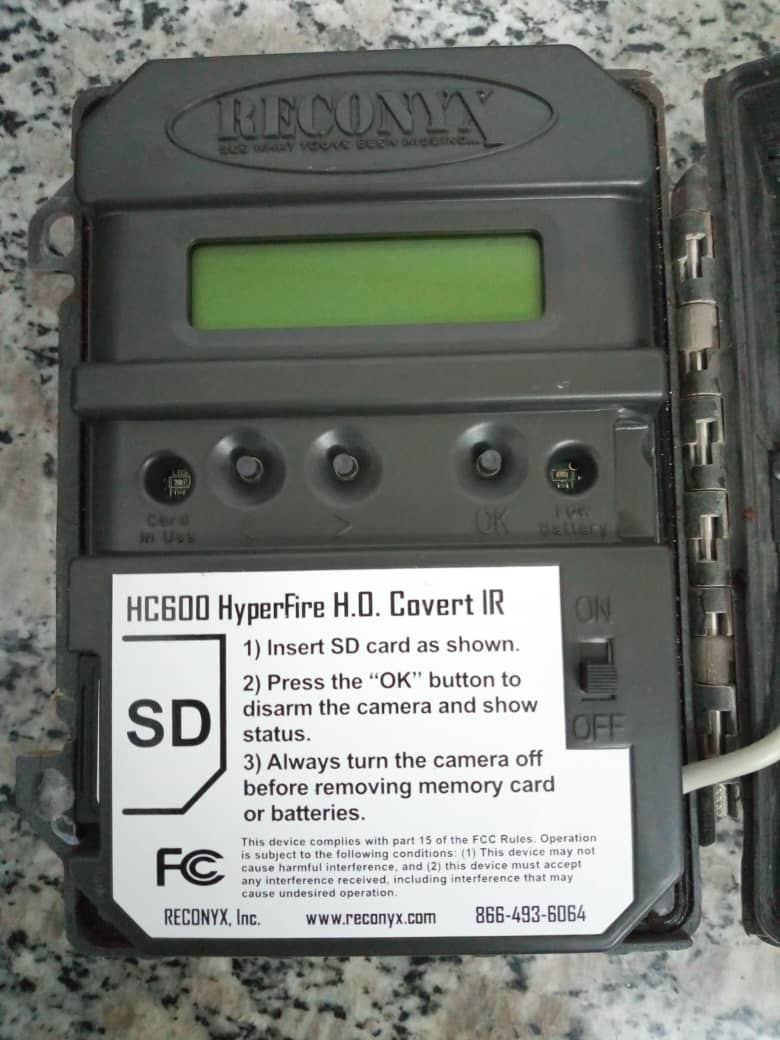

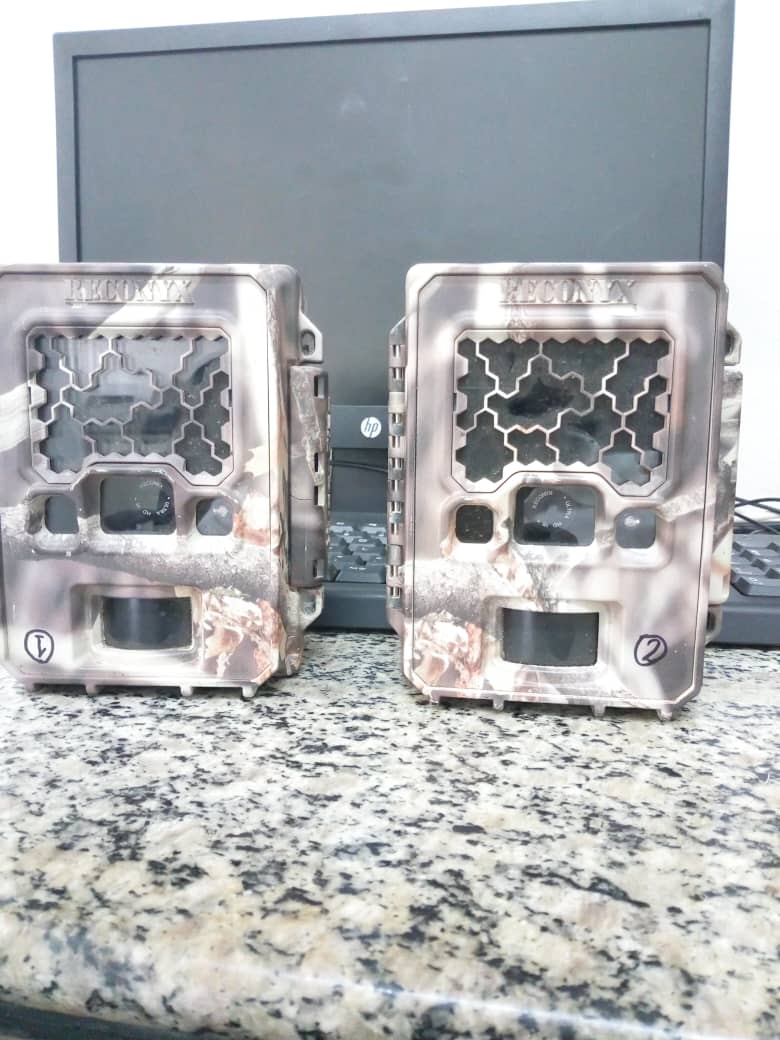


Reconyx^TM^ Hyperfire HC600 camera traps


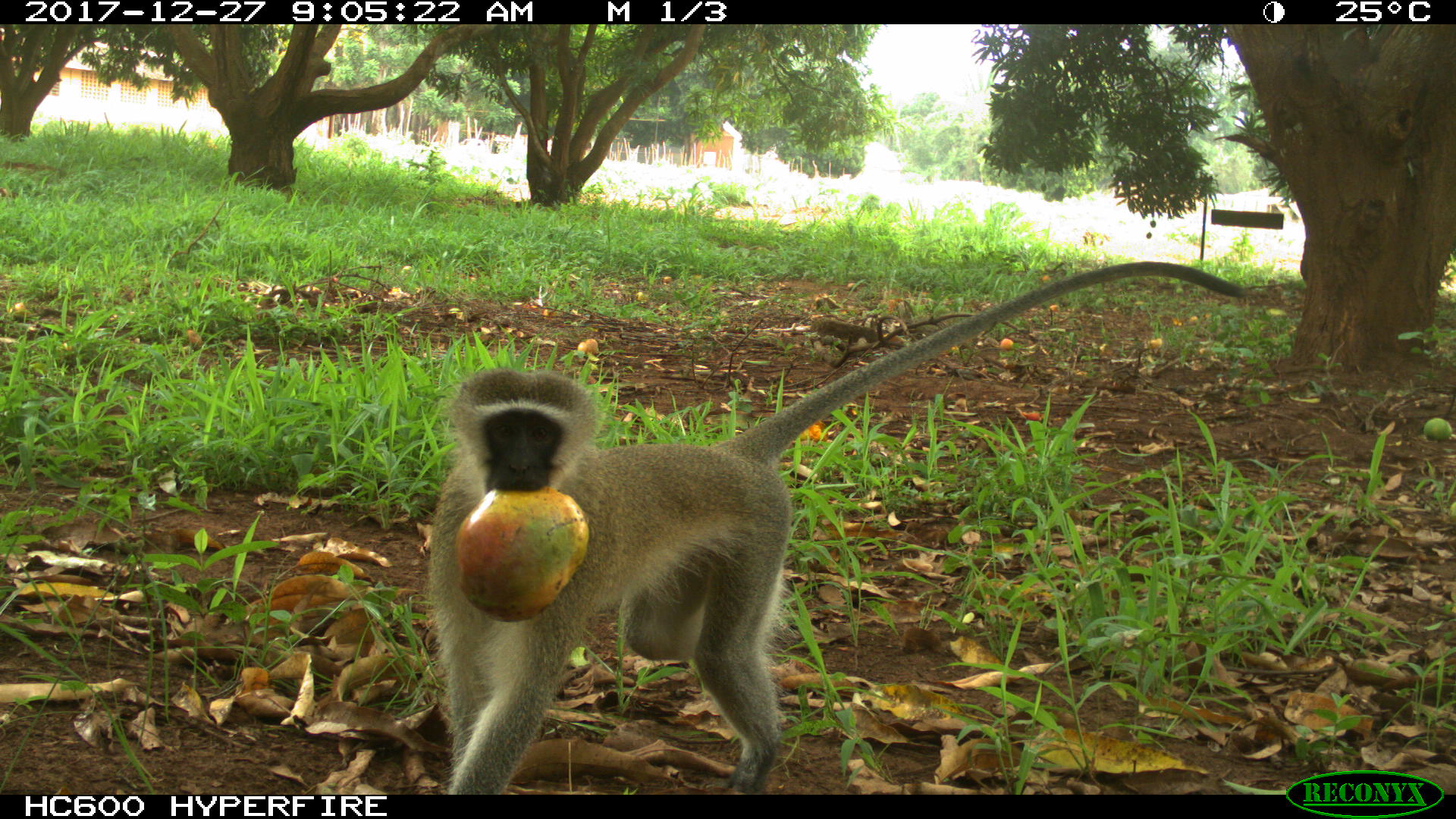
**Vervet Monkey (*Cercopithecus aethiops*)**
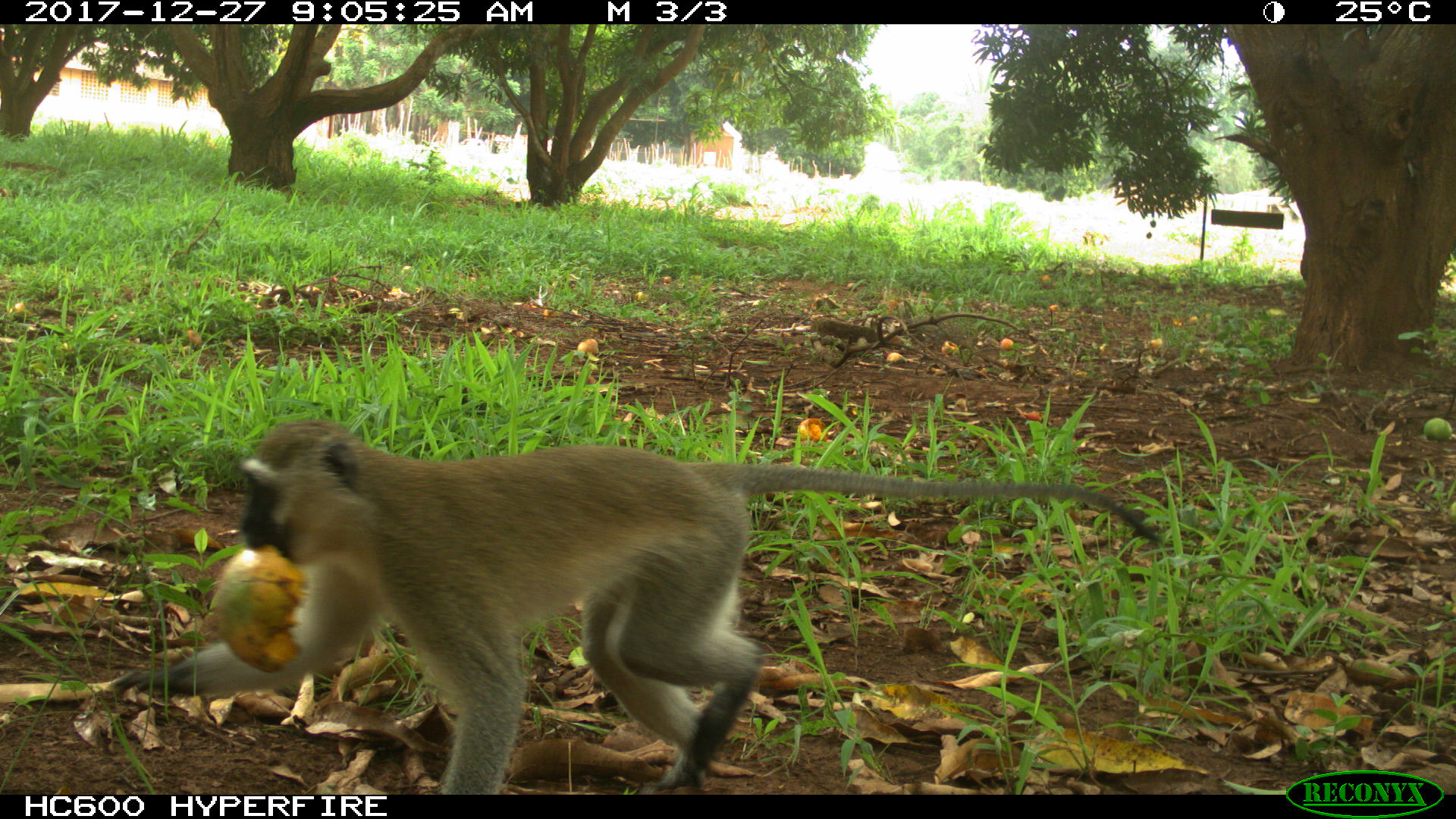

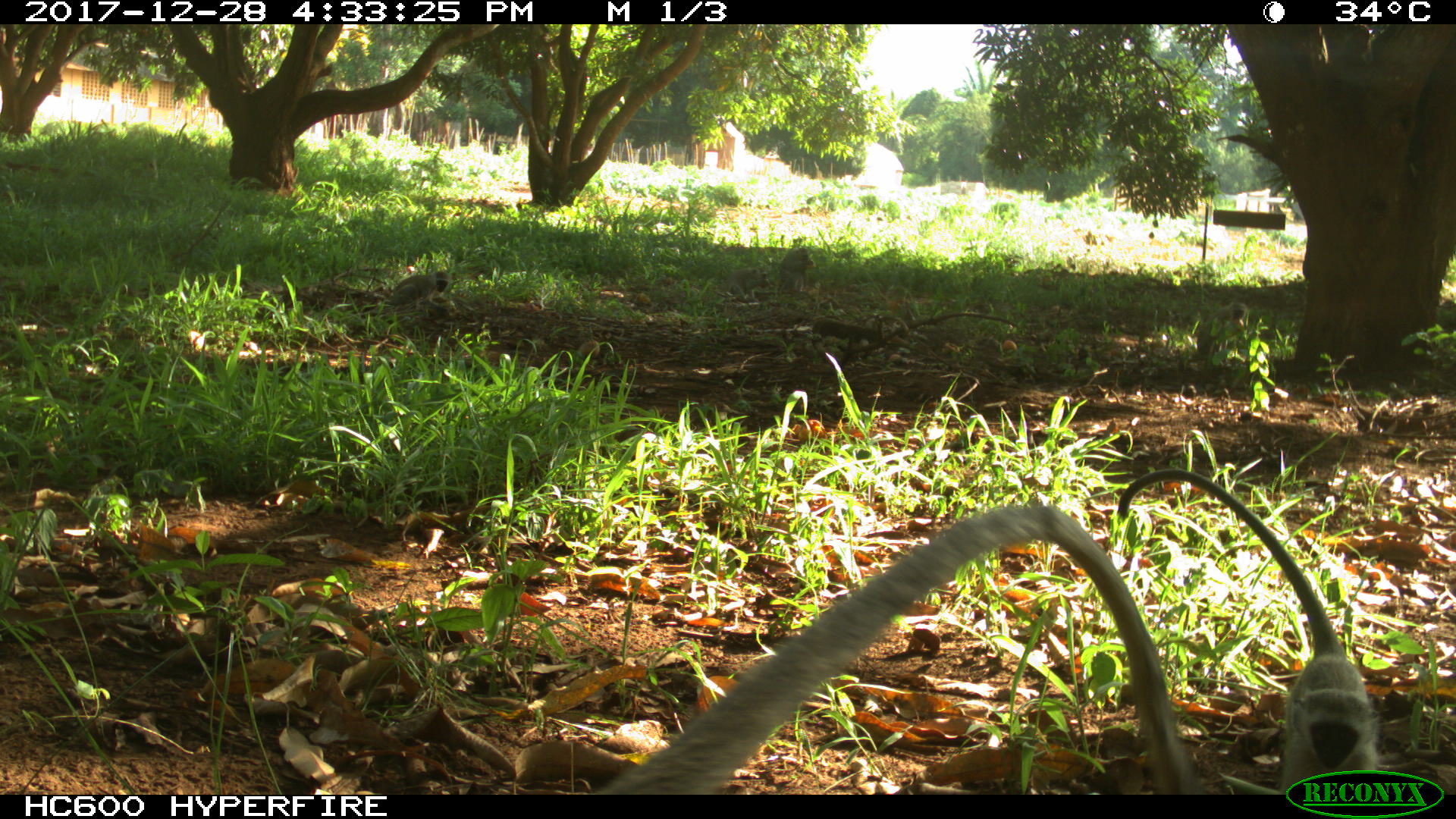

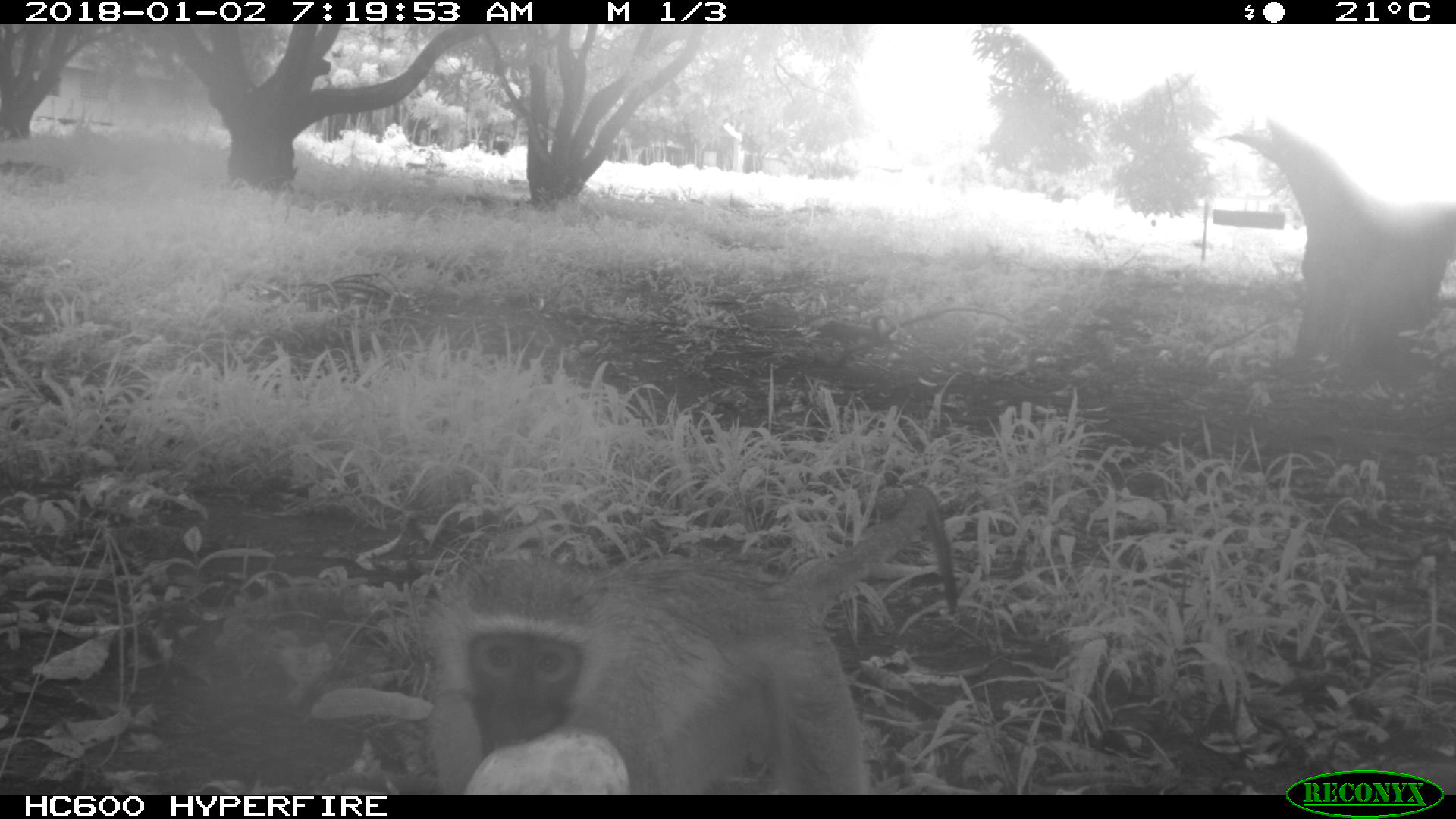


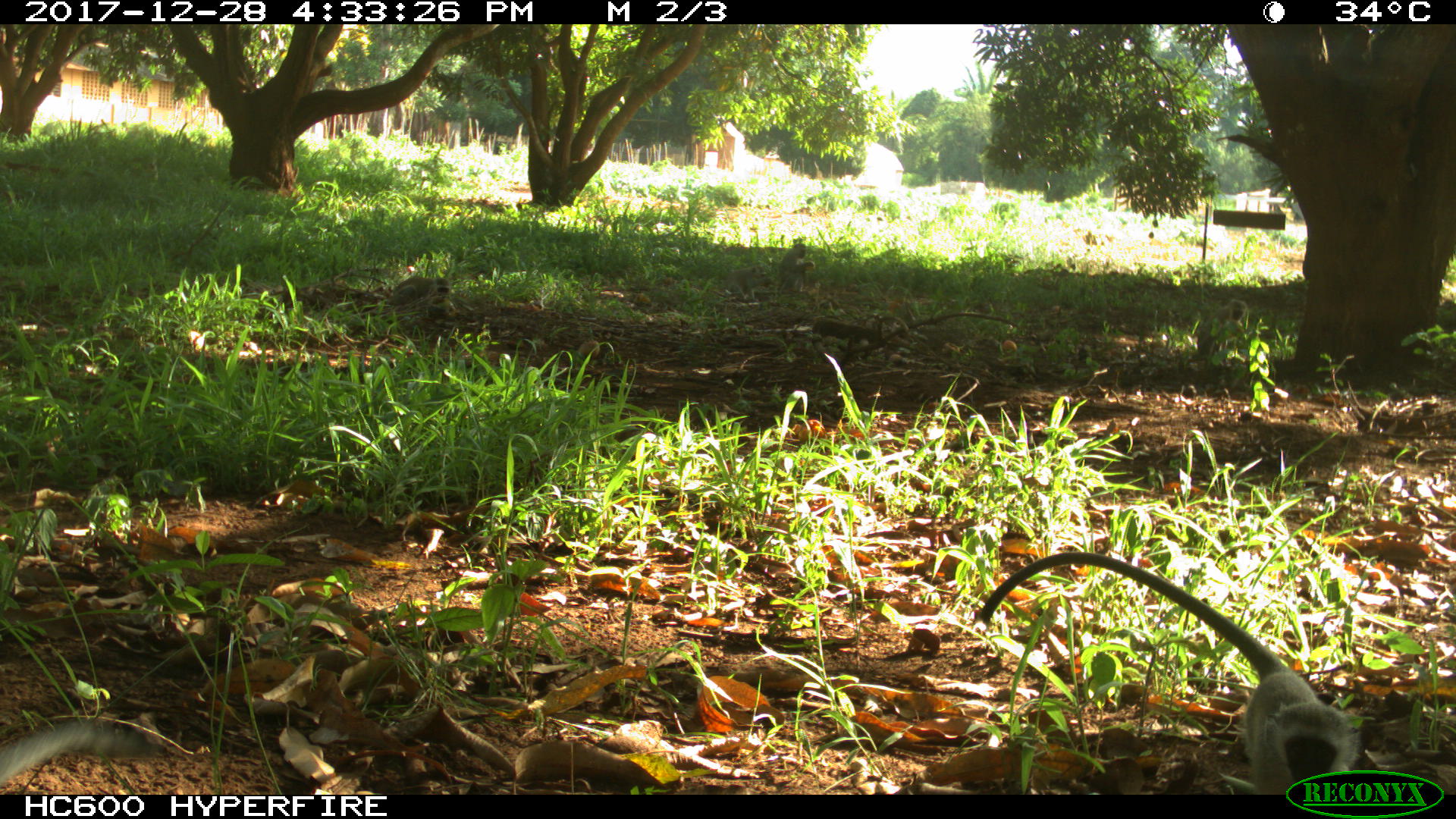

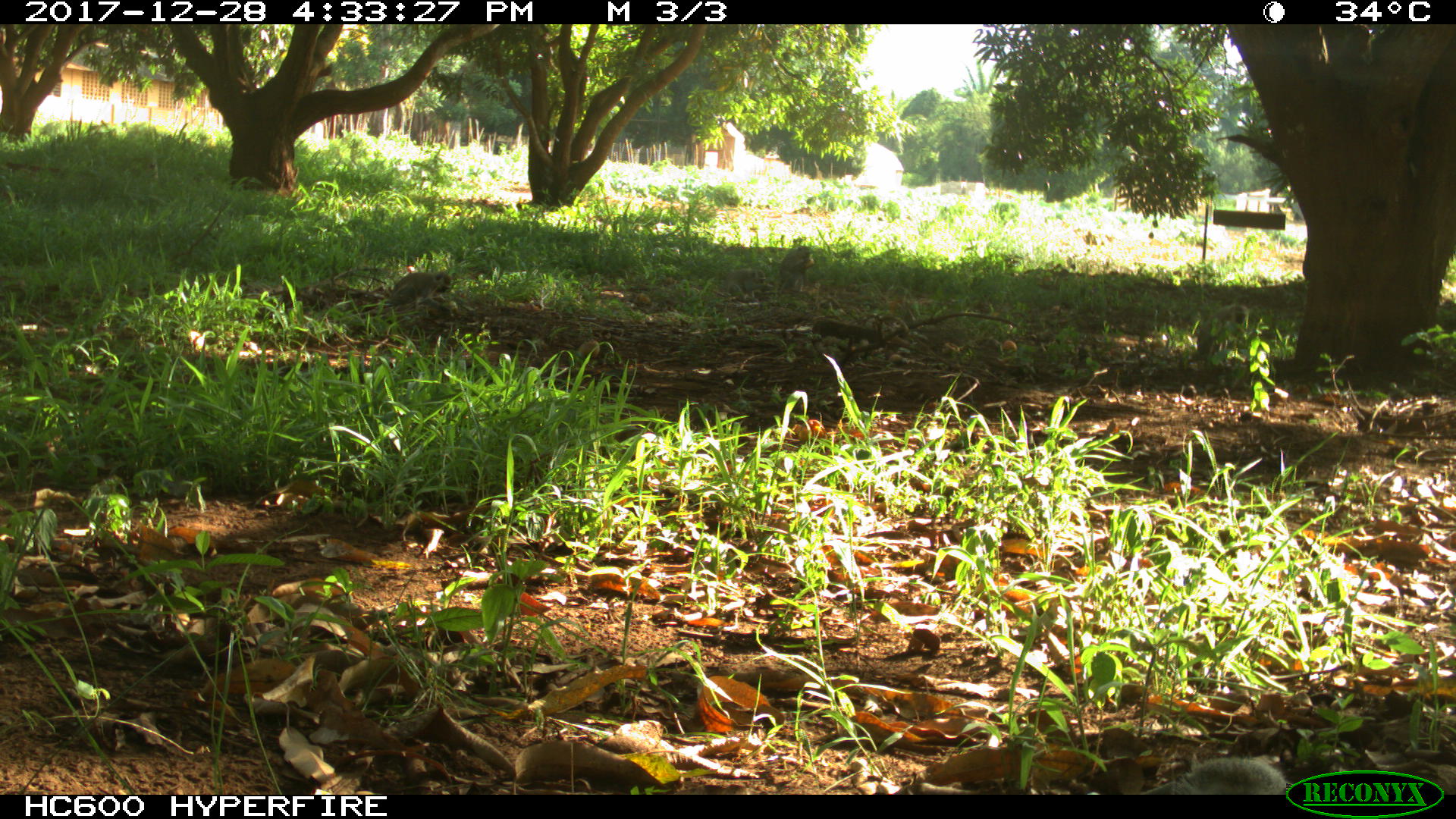

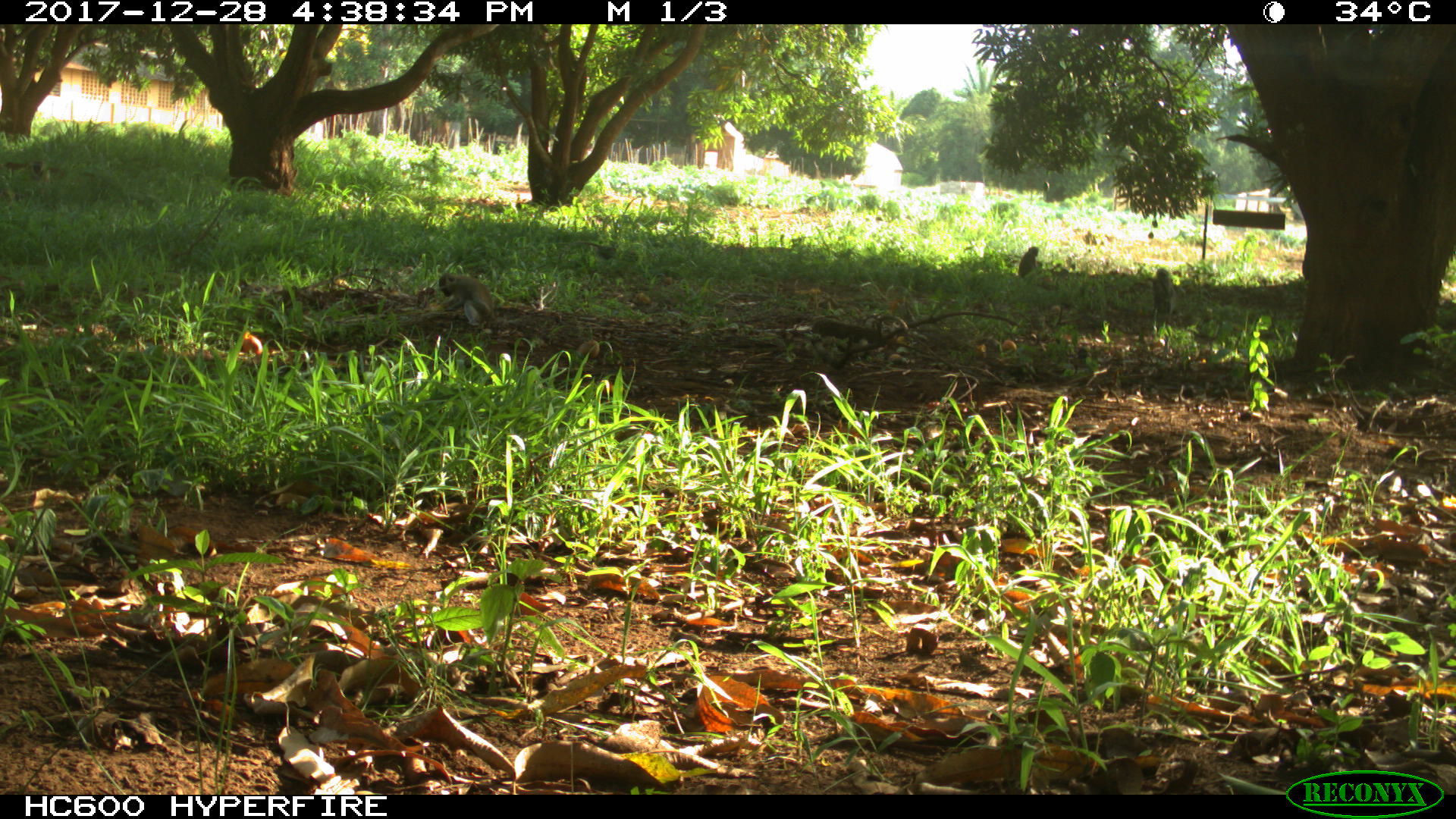

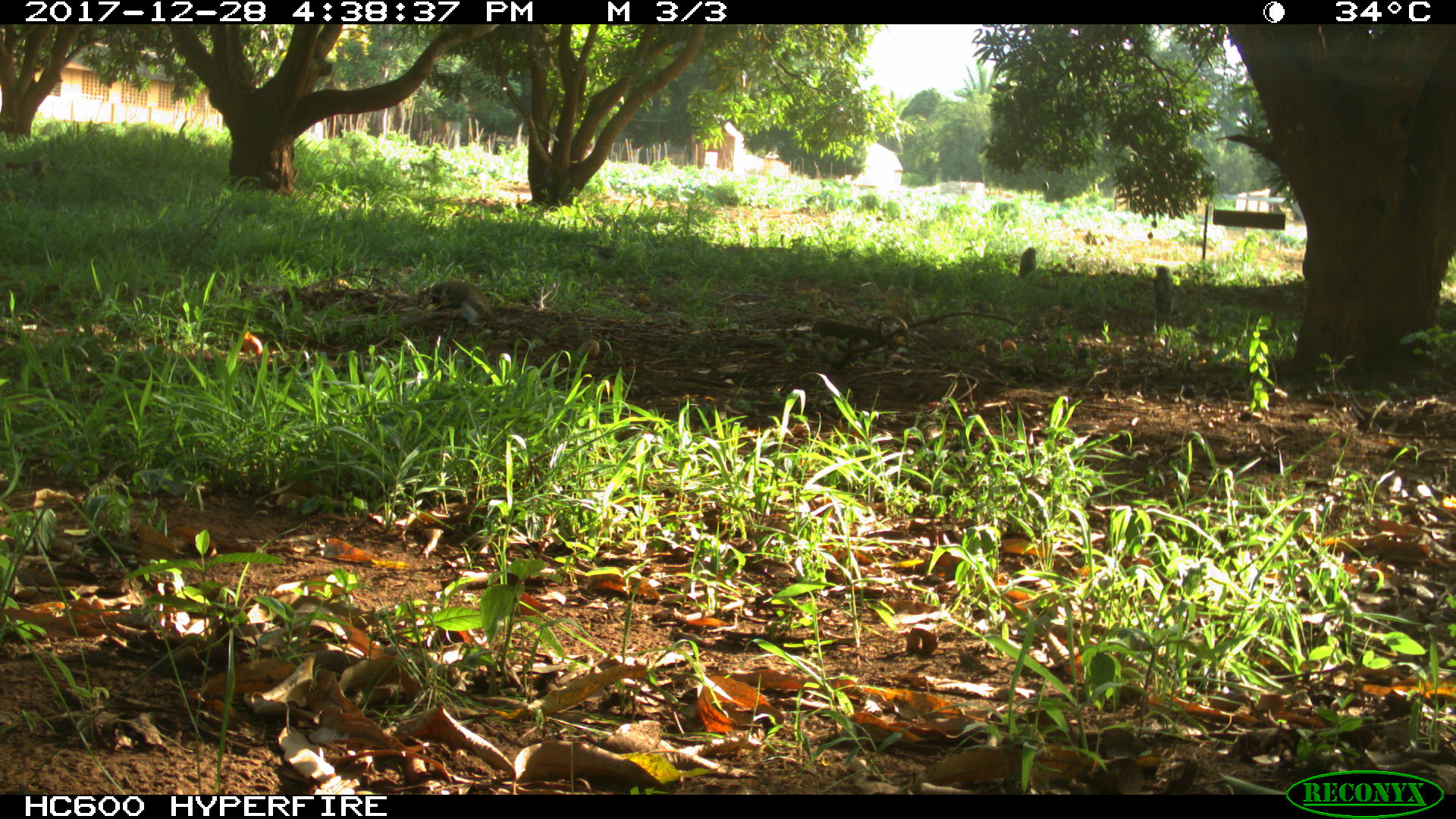

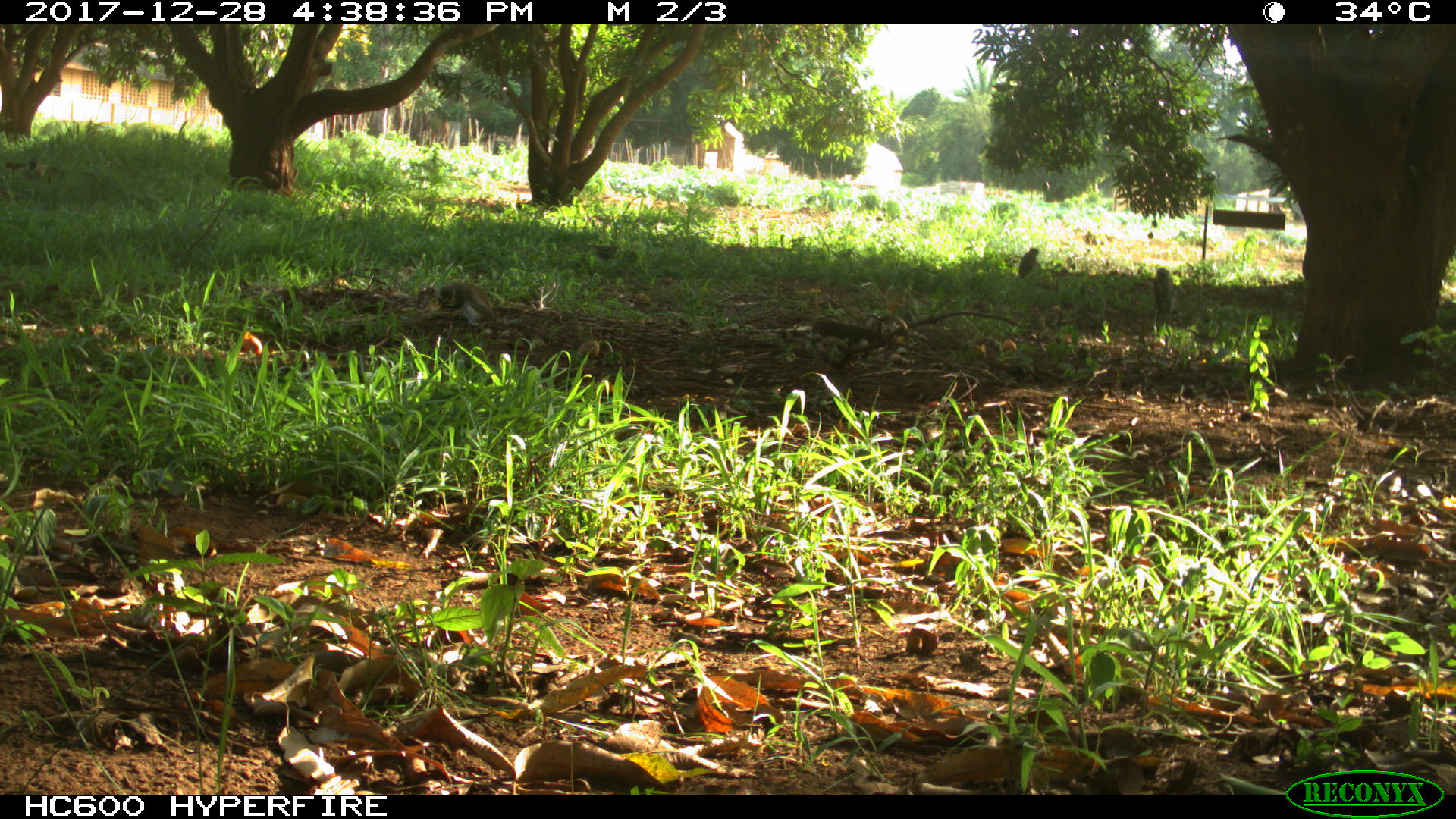

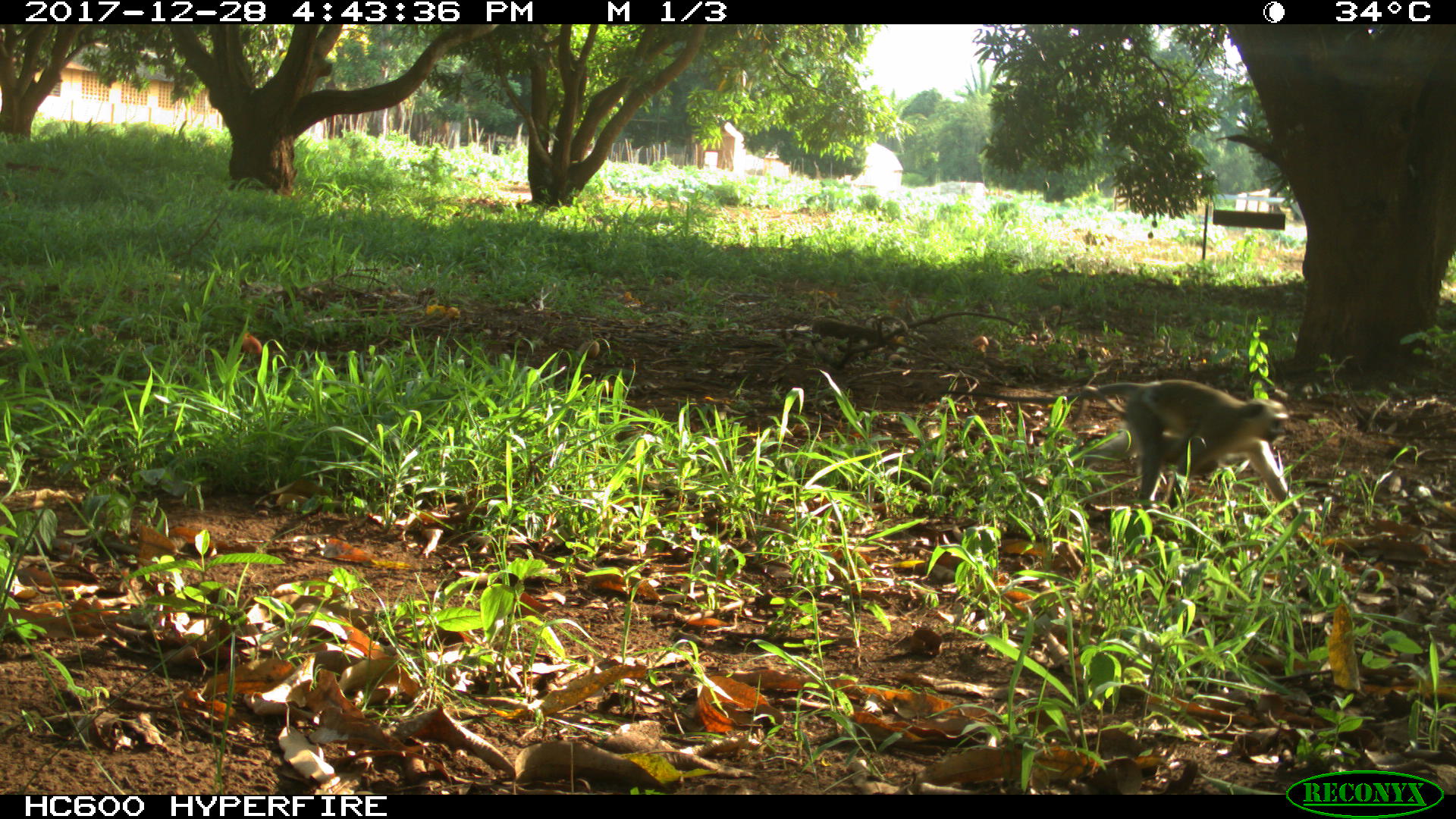

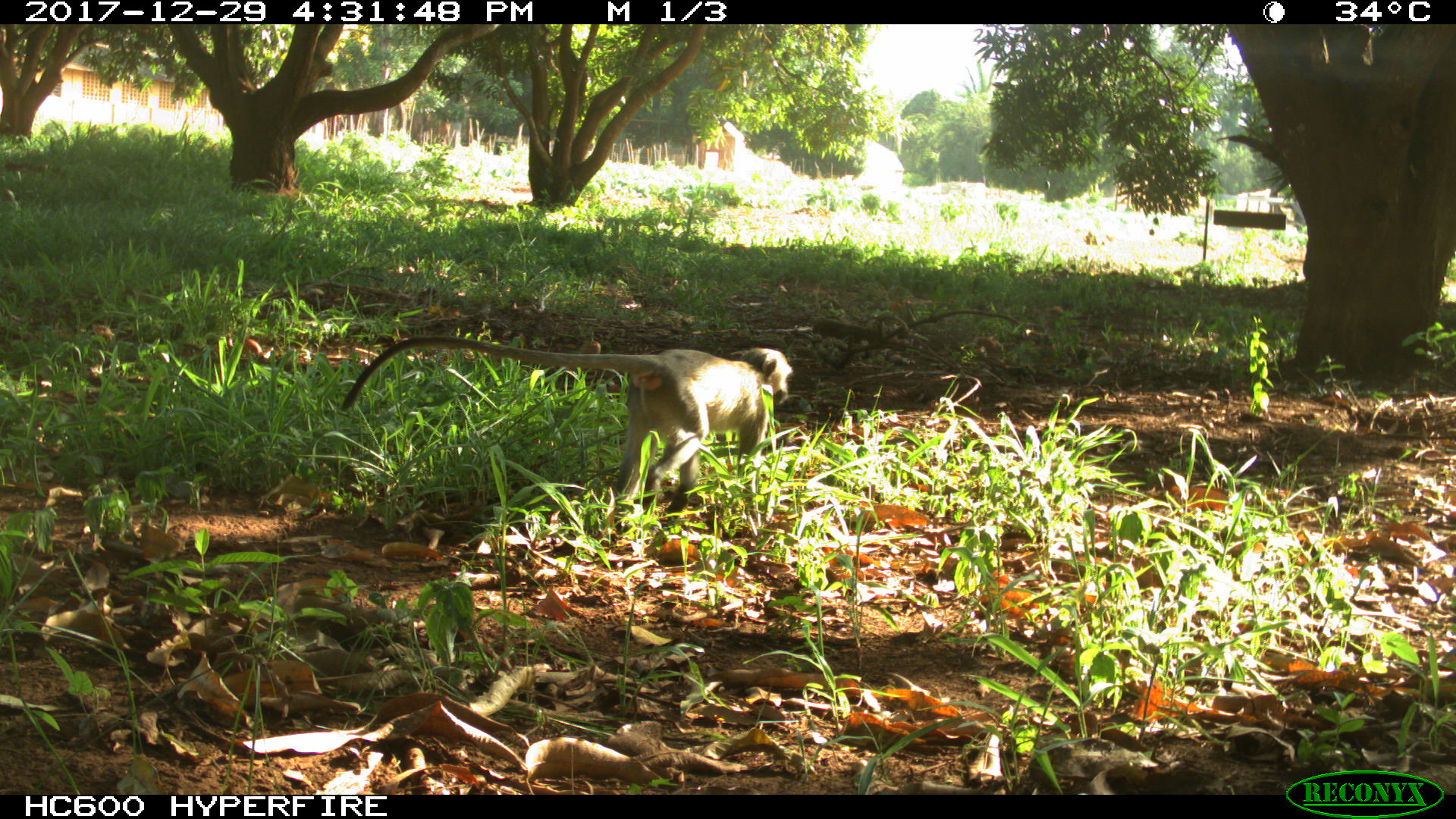

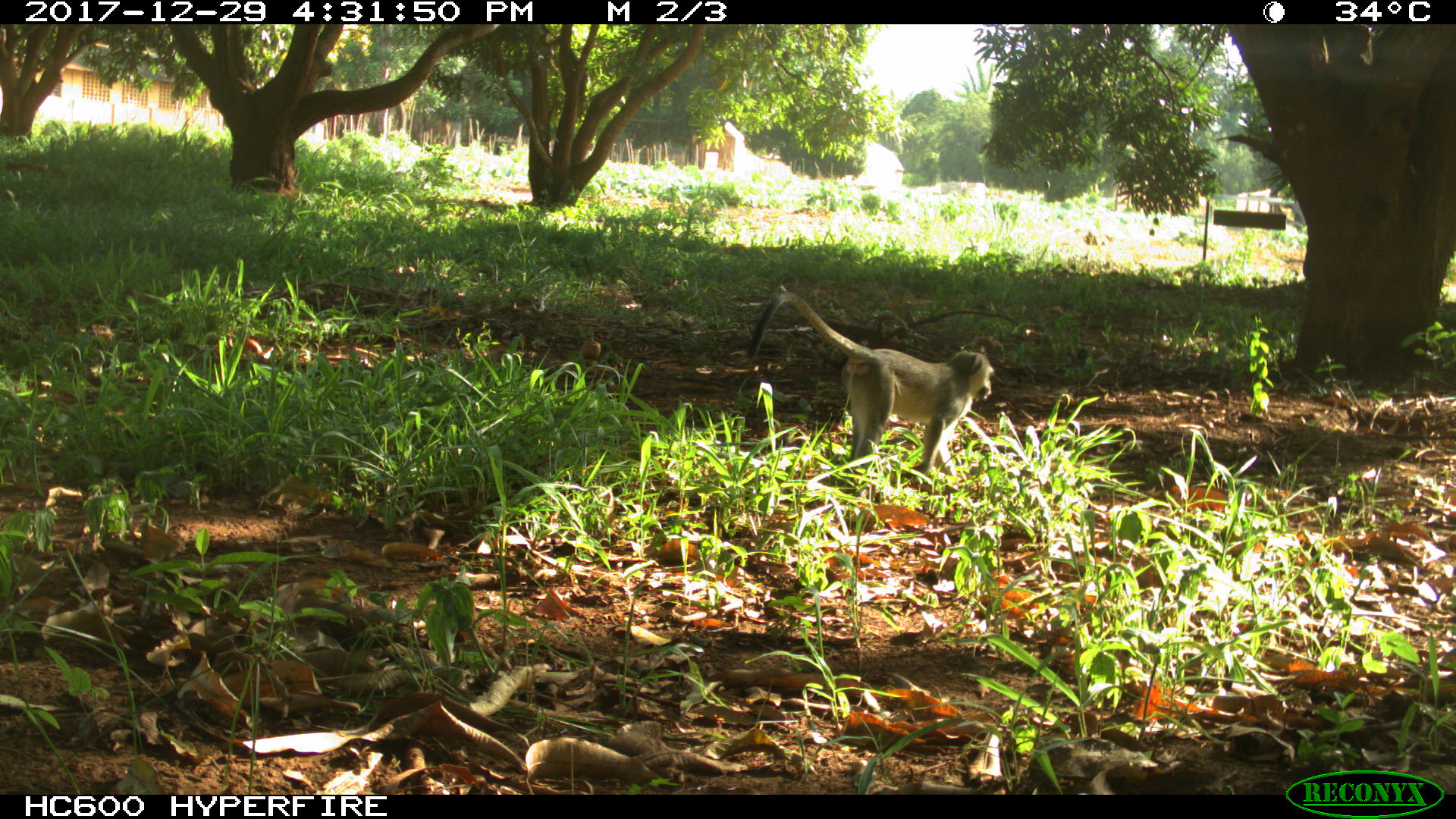


**Domestic cat (*Felis catus*)**


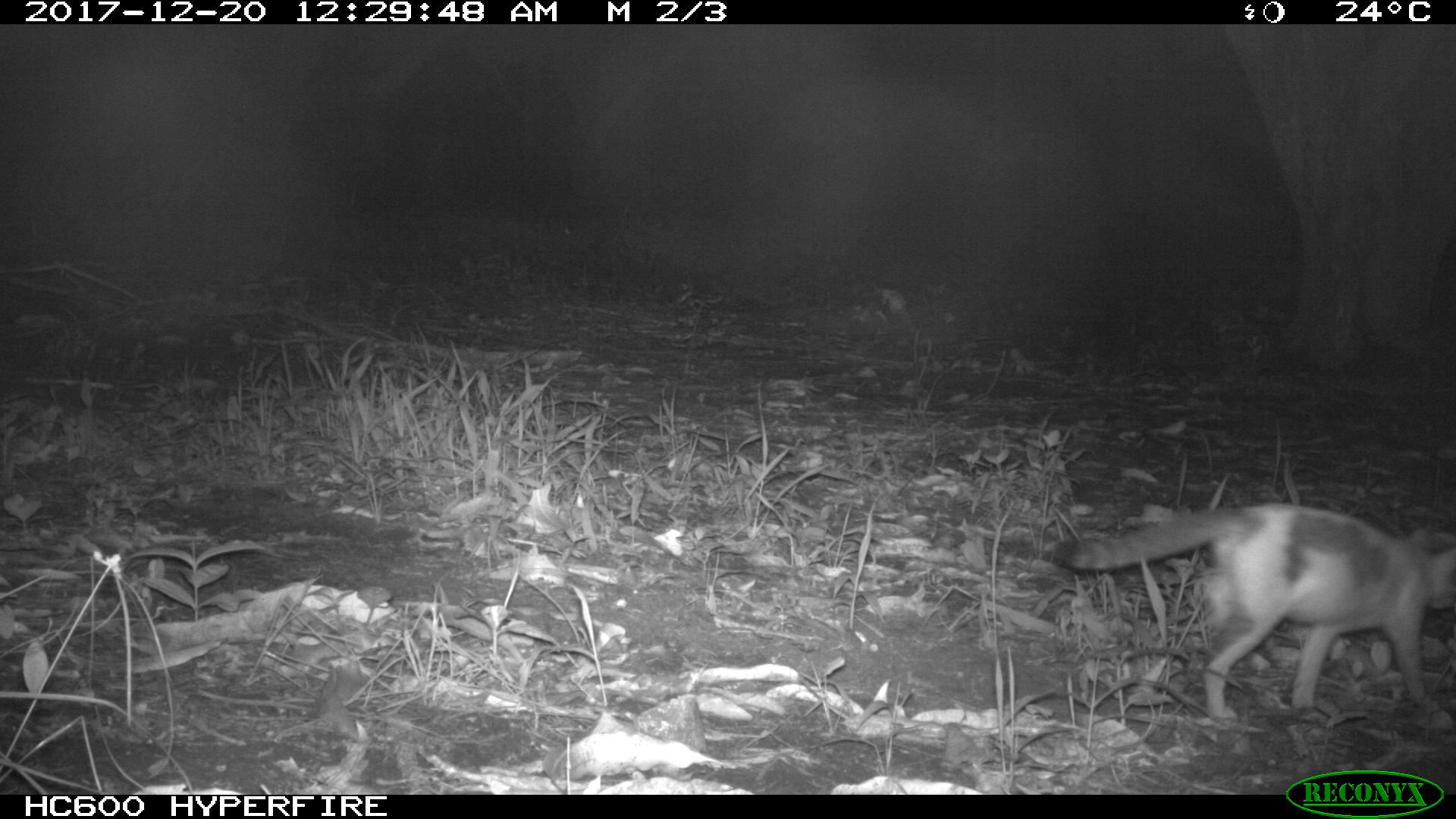


**Black rat *(Rattus rattus)***


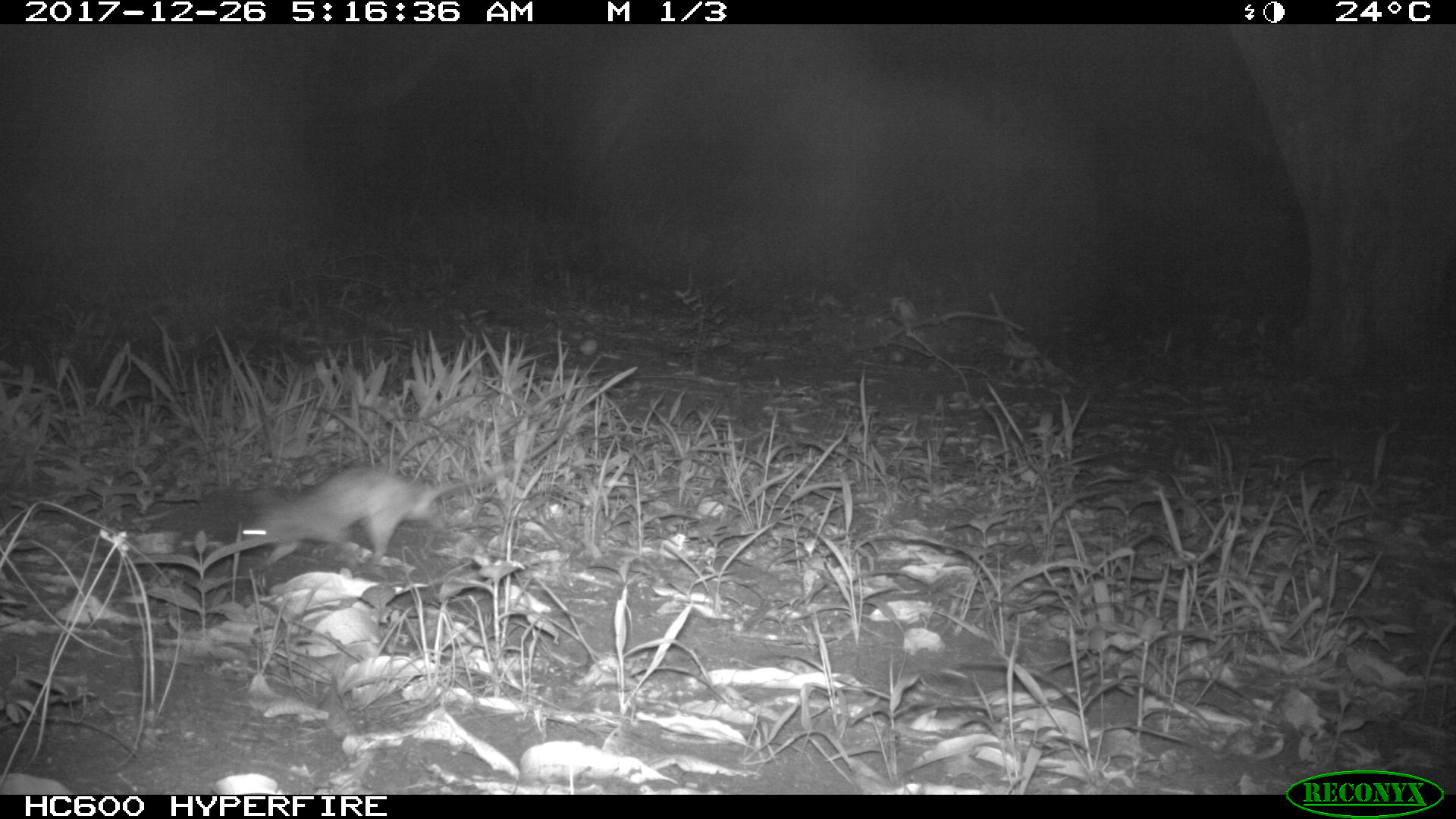


**Common dwarf Mongoose (*Helogale parvula*)**


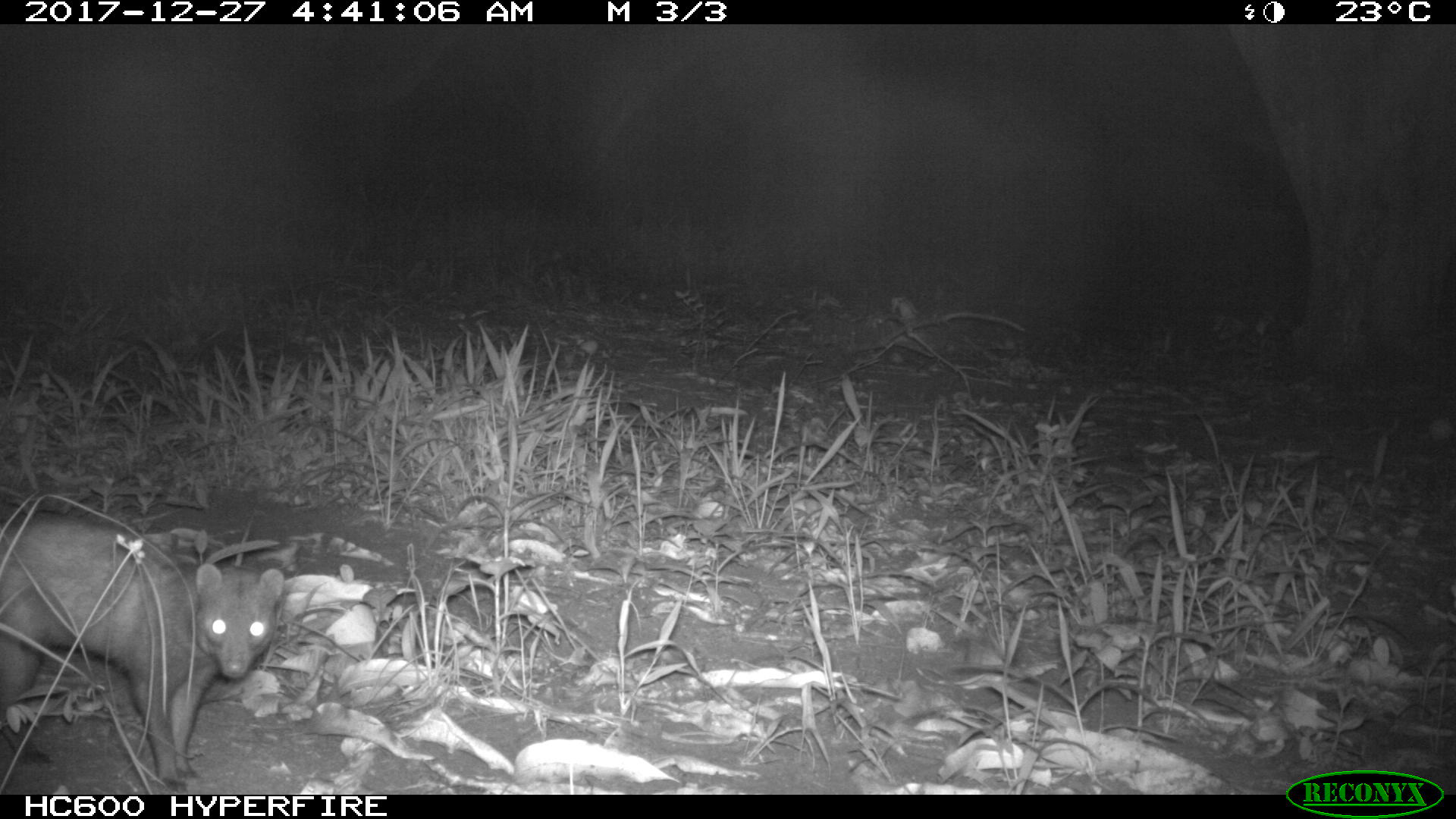

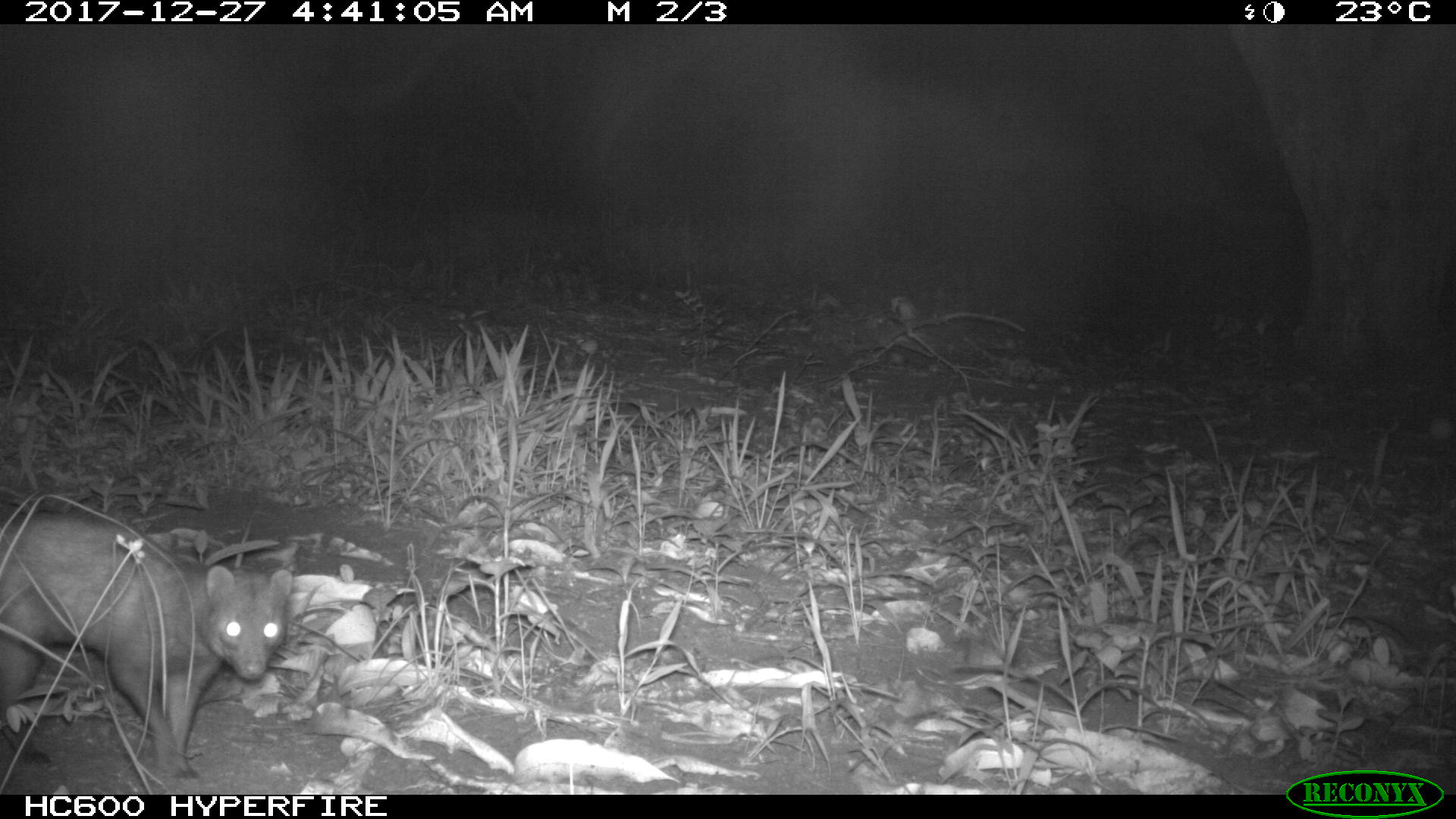


**Mongoose species (possibly dwarf mongoose)**


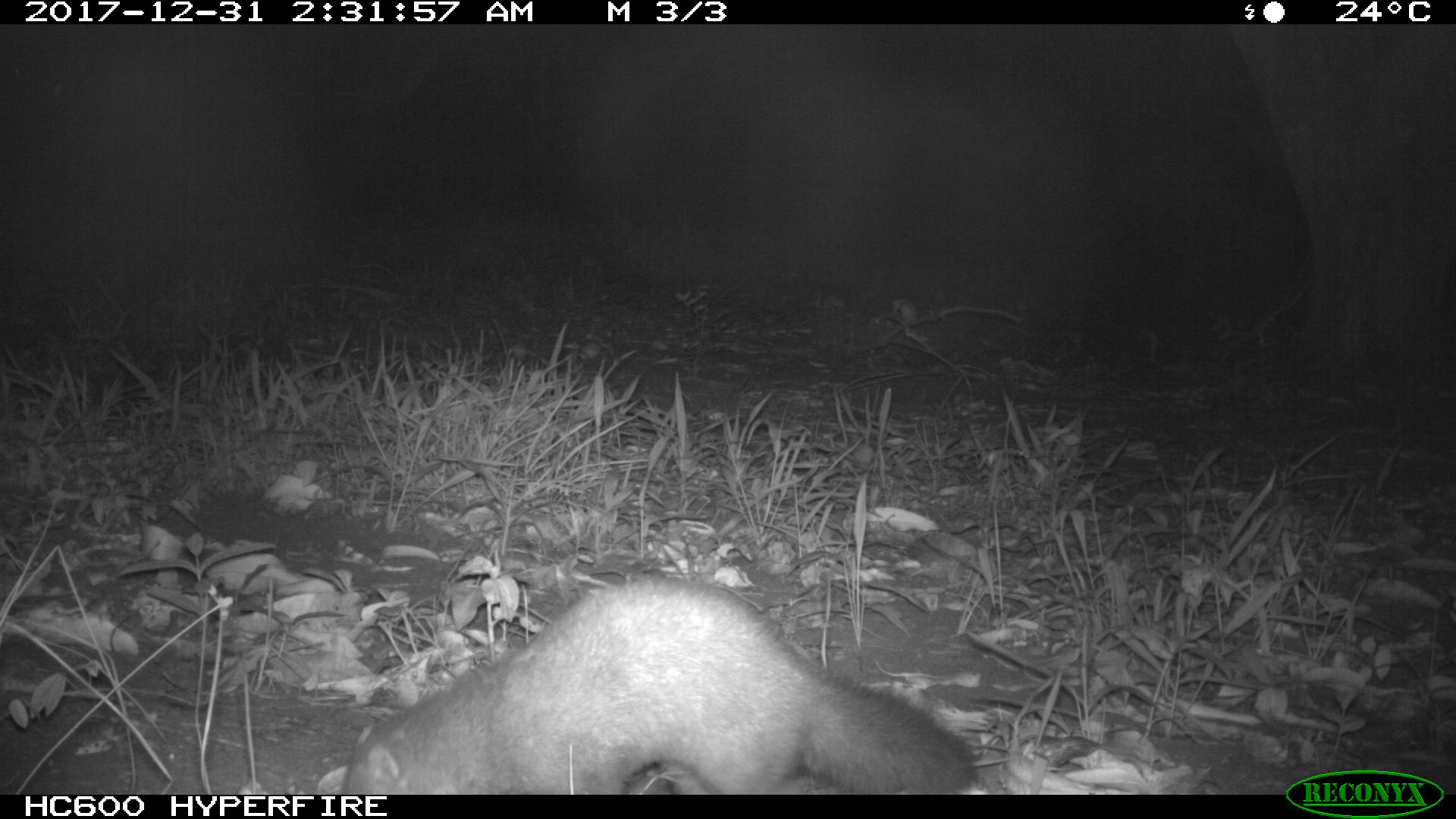

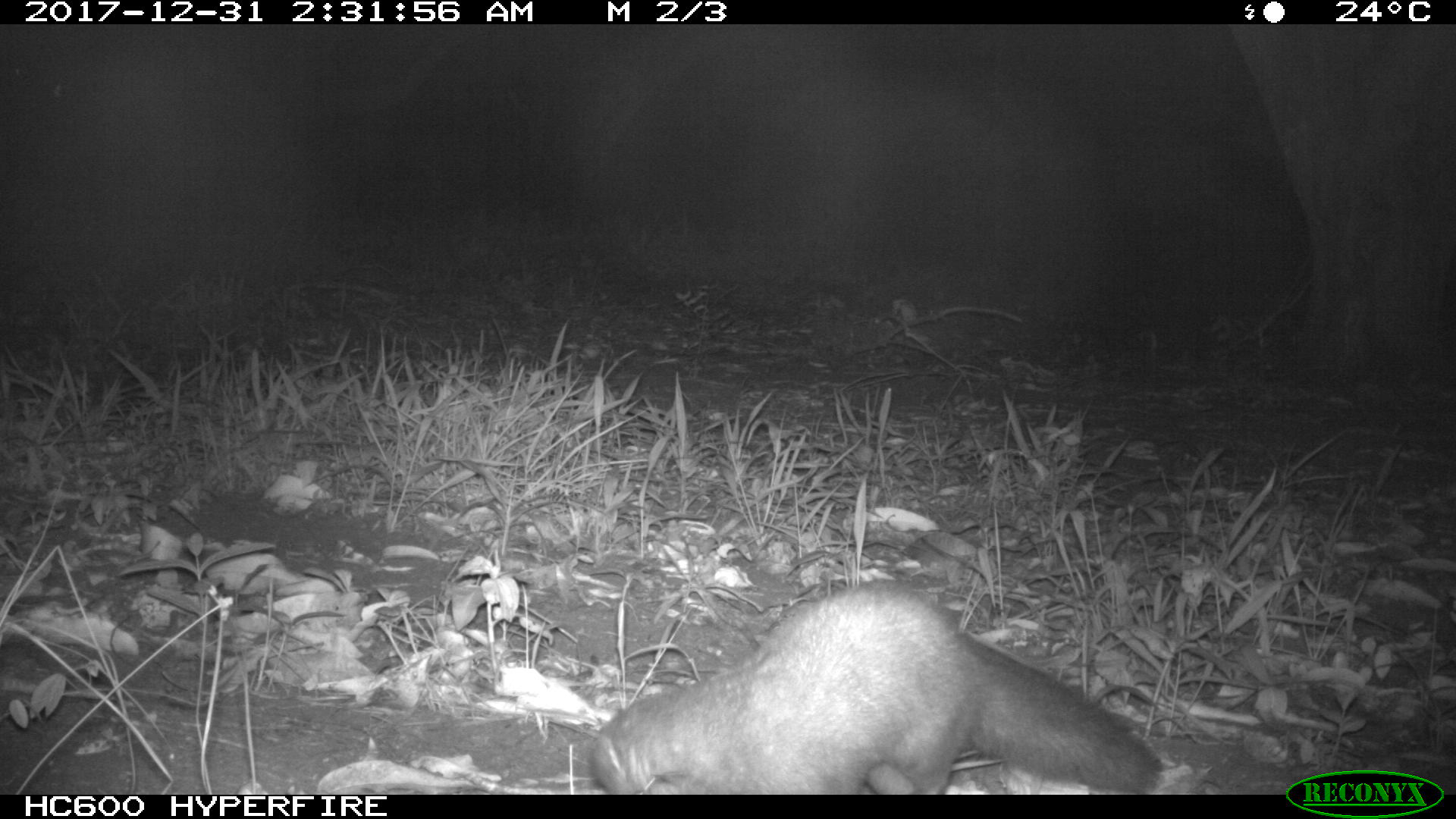


**Humans**

**
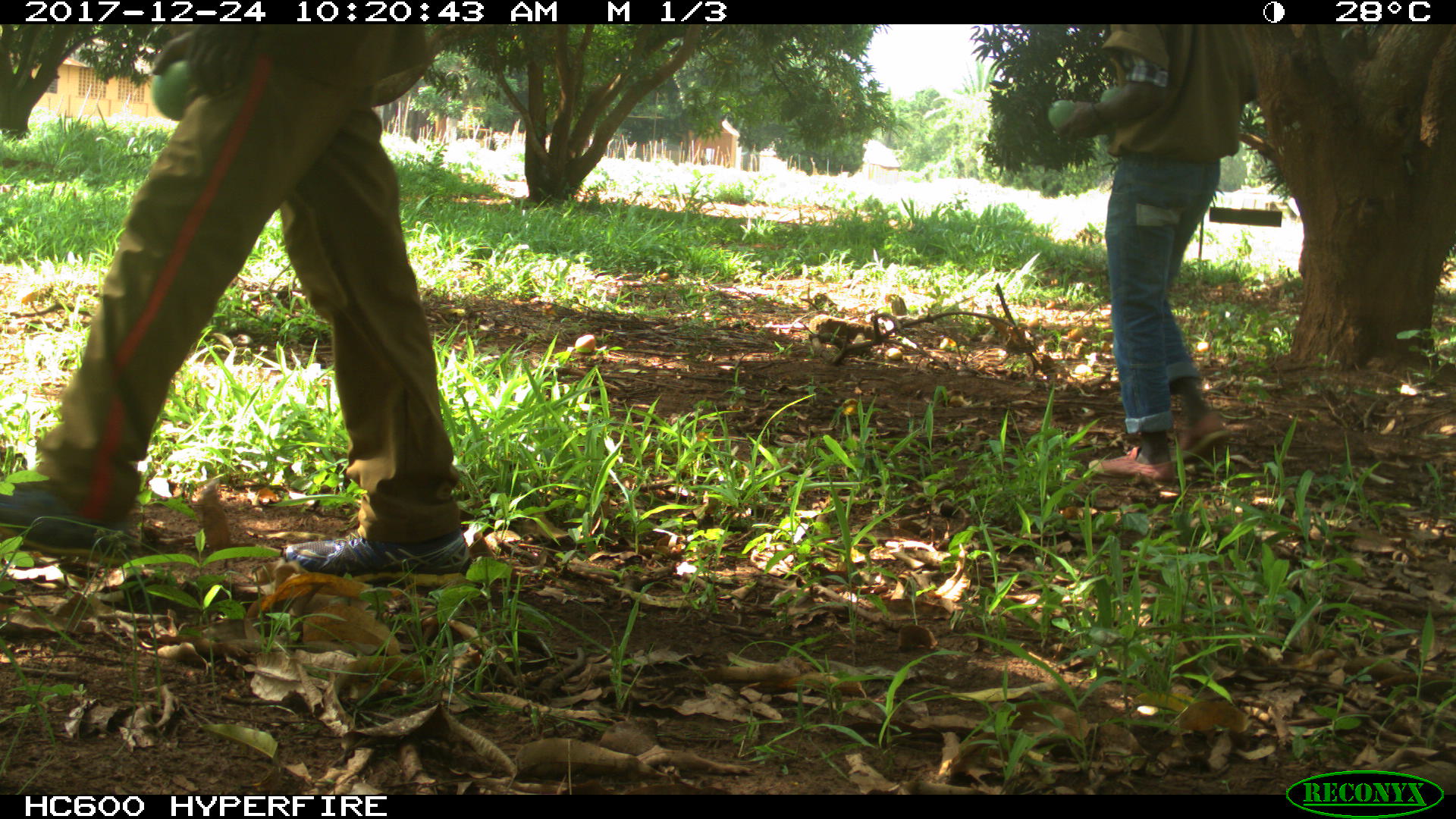
**
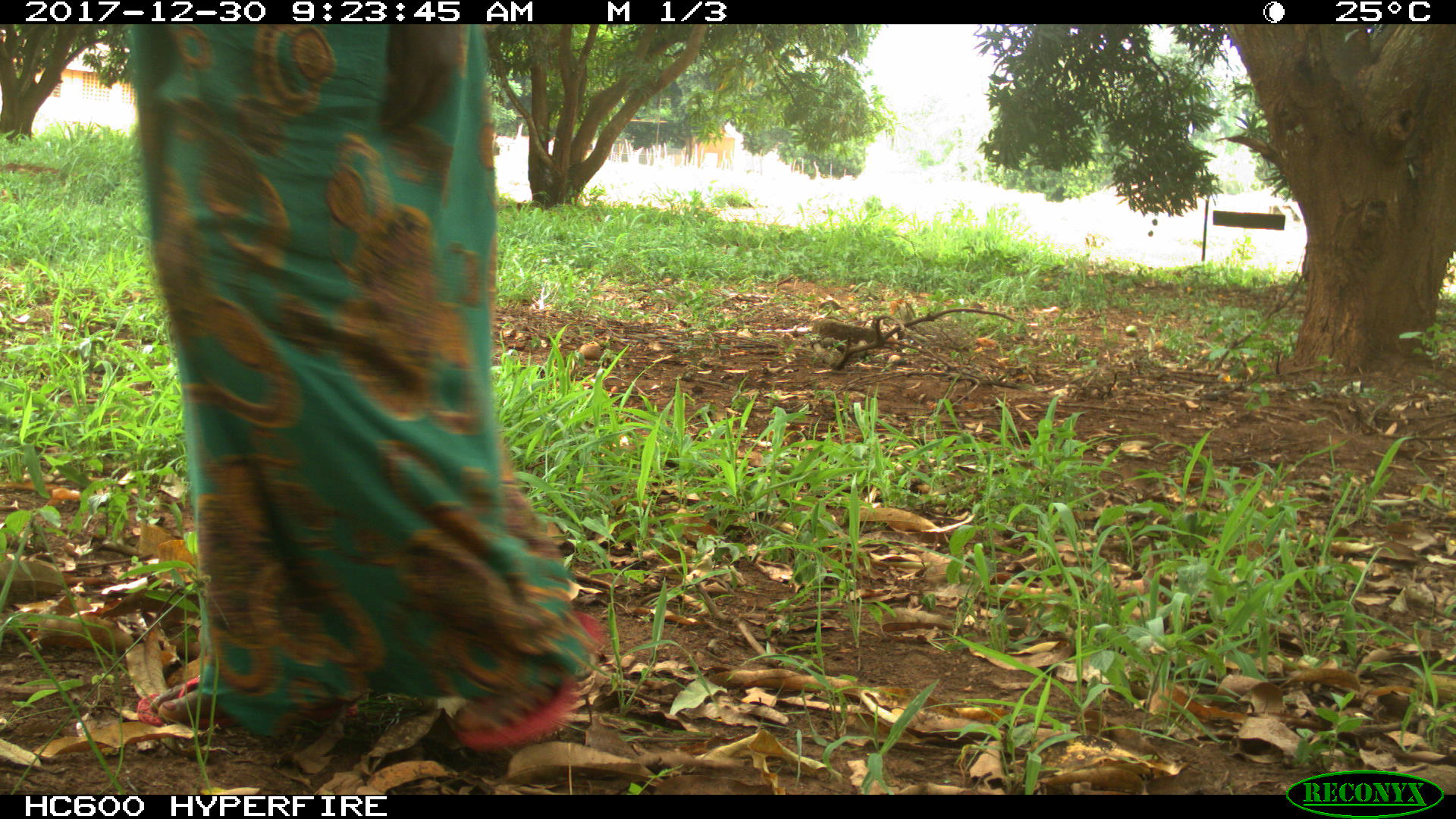

Supplement: Supplementary file 8 — Additional file 8. Pictures of camera traps and collected images. [file 42522_2020_20_MOESM8_ESM.docx]
